# Piperlongumine potentiates the effects of gemcitabine in *in vitro* and *in vivo* human pancreatic cancer models

**DOI:** 10.18632/oncotarget.23623

**Published:** 2017-12-23

**Authors:** Jiyan Mohammad, Harsharan Dhillon, Shireen Chikara, Sujan Mamidi, Avinash Sreedasyam, Kishore Chittem, Megan Orr, John C. Wilkinson, Katie M. Reindl

**Affiliations:** ^1^ Department of Biological Sciences, North Dakota State University, Fargo, ND 51808, USA; ^2^ Genome Sequencing Center, HudsonAlpha Institute for Biotechnology, Huntsville, AL 35806, USA; ^3^ Department of Plant Pathology, North Dakota State University, Fargo, ND 51808, USA; ^4^ Department of Statistics, North Dakota State University, Fargo, ND 51808, USA; ^5^ Department of Chemistry and Biochemistry, North Dakota State University, Fargo, ND 51808, USA

**Keywords:** apoptosis, cell cycle regulation, complementary and alternative therapy, reactive oxygen species, RNA-Seq

## Abstract

Pancreatic ductal adenocarcinoma (PDAC) is one of the deadliest cancers due to a late diagnosis and poor response to available treatments. There is a need to identify complementary treatment strategies that will enhance the efficacy and reduce the toxicity of currently used therapeutic approaches. We investigated the ability of a known ROS inducer, piperlongumine (PL), to complement the modest anti-cancer effects of the approved chemotherapeutic agent gemcitabine (GEM) in PDAC cells *in vitro* and *in vivo*. PDAC cells treated with PL + GEM showed reduced cell viability, clonogenic survival, and growth on Matrigel compared to control and individually-treated cells. Nude mice bearing orthotopically implanted MIA PaCa-2 cells treated with both PL (5 mg/kg) and GEM (25 mg/kg) had significantly lower tumor weight and volume compared to control and single agent-treated mice. RNA sequencing (RNA-Seq) revealed that PL + GEM resulted in significant changes in p53-responsive genes that play a role in cell death, cell cycle, oxidative stress, and DNA repair pathways. Cell culture assays confirmed PL + GEM results in elevated ROS levels, arrests the cell cycle in the G0/G1 phase, and induces PDAC cell death. We propose a mechanism for the complementary anti-tumor effects of PL and GEM in PDAC cells through elevation of ROS and transcription of cell cycle arrest and cell death-associated genes. Collectively, our results suggest that PL has potential to be combined with GEM to more effectively treat PDAC.

## INTRODUCTION

Pancreatic ductal adenocarcinoma (PDAC) is the third most common cause of cancer death in the United States with a 5-year relative survival rate of only 8% [1]. Though surgery is the most effective treatment for PDAC, the majority of pancreatic tumors are not surgically resectable. A standard treatment approach for non-resectable pancreatic tumors is the use of chemotherapeutic agents that are toxic to cancer cells as well as healthy cells.

Gemcitabine (GEM), a cytidine analog that inhibits DNA synthesis and DNA repair, is currently a drug of choice for treating PDAC [2]. However, treatment with GEM leads to only a modest improvement in overall survival of PDAC patients [3]. To increase GEM's efficacy, adjuvant therapies such as 5-fluorouracil, nab-paclitaxel, or cisplatin are often used [4]. These agents target other mechanisms used by cancer cells to grow and divide. However, these approaches have had limited success, and only extend life span by a few months with additional toxicity [5–7]. Therefore, there is a need for complementary approaches that effectively inhibit tumor progression without introducing more toxicity to healthy cells.

Piperlongumine (PL) is a bioactive agent derived from the fruits and roots of the long pepper plant. PL has been shown to kill numerous cancer cell types without affecting normal cells [8, 9]. We have previously shown that PL inhibits PDAC cell proliferation *in vitro* and *in vivo* by enhancing ROS and DNA damage [10]. Further, we have identified that oxidative stress and endoplasmic reticulum stress-associated genes are significantly altered in PDAC cells upon PL treatment [11]. We postulate that using PL in combination with GEM will enhance PDAC cell death by raising ROS to a level that induces cell death. The rationale for this therapeutic approach is that highly metabolic tumor cells have heightened basal oxidative stress and are susceptible to cell death when additional oxidative stress is induced [12]. Here, we demonstrate the ability of PL to enhance the effects of GEM for PDAC treatment. Further, we identified the underlying mechanisms for the enhanced anti-tumor effects of PL + GEM in PDAC cells which include elevation of ROS and differential expression genes including cell cycle and apoptosis-associated genes.

## RESULTS

### PL enhances the effects of GEM on PDAC cell viability *in vitro*

**Figure 1:**
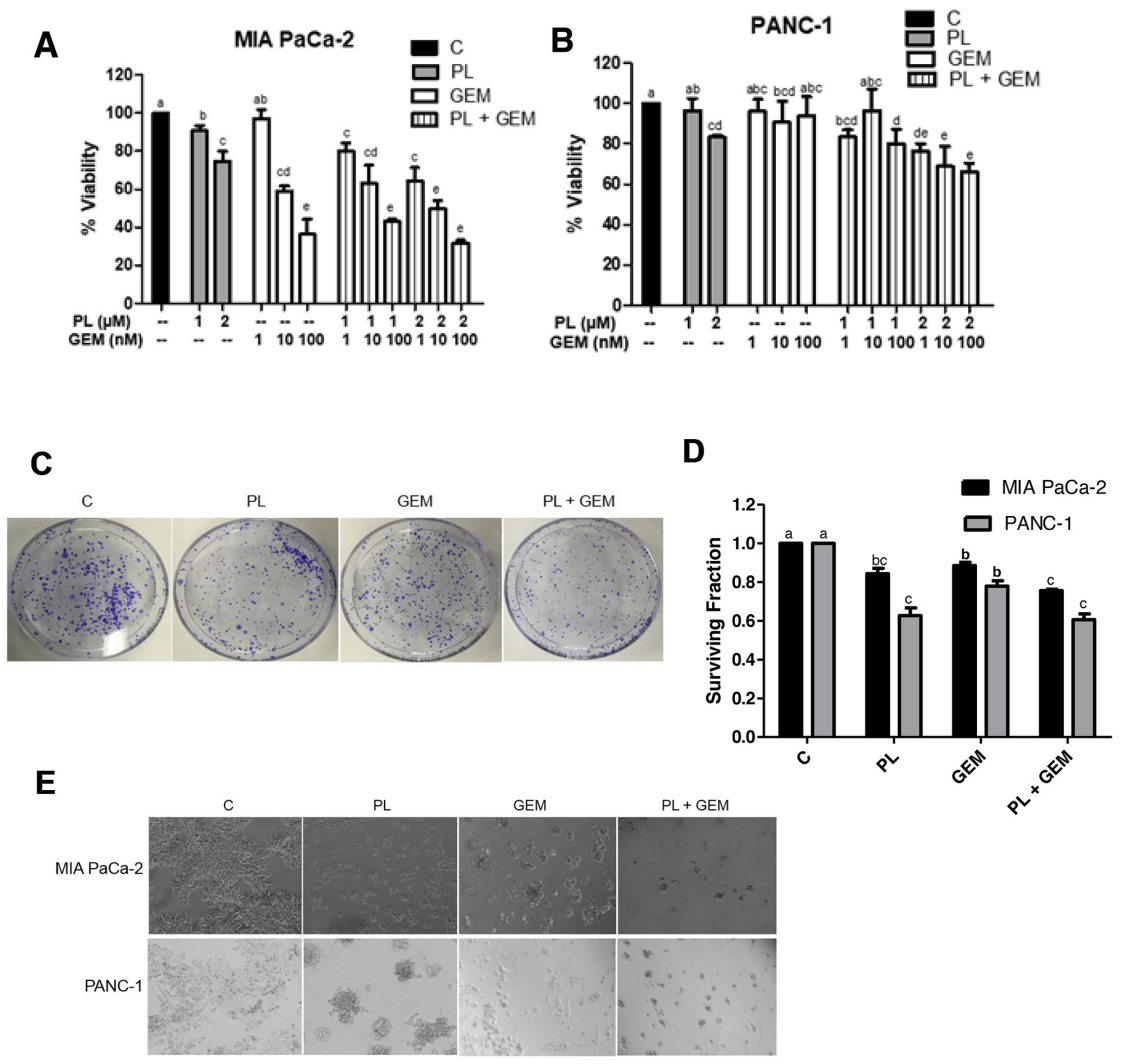
Effect of GEM, PL, and their combination on *in vitro* cell viability, clonogenic survival, and growth on Matrigel Cell viability percentages were determined using an MTT assay for **(A)** MIA PaCa-2 and **(B)** PANC-1 cells treated with vehicle control (C), PL (1 or 2 μM), GEM (1-100 nM), or their combinations for 72 h. The data shown in the bar graphs represent the average percent viability relative to the vehicle-treated control ± SE for three independent experiments for both cell lines. Clonogenic survival assays were performed for MIA PaCa-2 and PANC-1 cells treated with C, PL (1 μM), GEM (1 nM), or PL (1 μM) + GEM (1 nM) for 10 days. **(C)** Results from a typical clonogenic survival experiment are shown for the MIA PaCa-2 cell line. **(D)** The number of colonies formed relative to the number of cells seeded (surviving fraction) was determined for MIA PaCa-2 and PANC-1 cells treated with PL, GEM, or their combination relative to the vehicle-treated controls. The data shown in the bar graph represent the average surviving fraction relative to vehicle-treated controls ± SE for three independent experiments for each cell line. **(E)** MIA PaCa-2 and PANC-1 cells were grown on Matrigel and treated with C, PL (1 μM), GEM (1 μM), or PL (1 μM) + GEM (1 μM) for 4 days. The experiment was performed three times and the images show one representative experiment for each cell line. Treatments with bars that do not share a letter have differences that are statistically significant at P ≤ 0.05.

The combination of GEM with additional agents has shown modest survival benefits for PDAC patients compared to single-agent gemcitabine [5–7]. To determine if PL could enhance the effects of GEM on the viability of PDAC cells, we conducted MTT assays in both MIA PaCa-2 and PANC-1 cells. These two cell lines are both derived from poorly differentiated primary tumors and exhibit KRAS and TP53 mutations, and homozygous deletion of p16 [17]. However, PANC-1 cells are much more resistant to GEM than MIA PaCa-2 cells and represent a nice model to identify agents that might potentiate the effects of GEM [18]. The concentrations of PL and GEM were optimized for the MTT and other cell-based experiments (data not shown). Cells were then treated with PL (1 or 2 μM), GEM (1, 10, or 100 nM), or their combinations for 72 h. Cell viability was significantly reduced in PL + GEM-treated MIA PaCa-2 and PANC-1 cells compared to vehicle-treated controls and single agent-treated cells (Figure 1A and 1B). Based on Jin's formula [19], 1μM PL + 100 nM GEM, 2 μM + 10 μM GEM, and 2 μM PL + 100 nM GEM had additive effects for MIA PaCa-2 cells. However, 1 μM PL + 1 nM GEM and 2 μM PL + 1 nM GEM had synergistic effects for these same cells. In PANC-1 cells, PL + GEM had synergistic effects for all combinations except 1 μM PL + 10 nM GEM which had an antagonistic effect for both cell lines. Similar to findings in literature, we noted MIA PaCa-2 cells were more sensitive than PANC-1 cells to GEM; therefore, the ability of PL to enhance GEM's antiproliferative effects were more apparent in PANC-1 cells than MIA PaCa-2 in this particular assay (Figure 1A and 1B). In general, the combination of PL + GEM showed a modest, but statistically significant, inhibition of PDAC cell viability than either treatment alone during this short-term assay.

### PL in combination with GEM reduces PDAC cell clonogenic survival

A longer-term assay (clonogenic survival) was employed to determine the ability of PL in combination with GEM to influence PDAC cell survival. MIA PaCa-2 and PANC-1 cells were treated with GEM (1 nM), PL (1 μM), or GEM (1 nM) + PL (1 μM), for 10 days after which the number of colonies formed was counted (Figure 1C). PL in combination with GEM significantly reduced the number of colonies (surviving fraction) compared to control and GEM for both cell lines (Figure 1D). In addition to a reduction in the number of colonies, the colony size appears to be smaller in the PL + GEM treatment (Figure 1C), suggesting that the combination also prevents clonogenic expansion of existing tumor cells.

### PL in combination with GEM reduces PDAC cell growth on Matrigel

To evaluate the effect of PL in combination with GEM in a more physiologically relevant culture system, we used a Matrigel growth assay. Matrigel is an extracellular matrix consisting of collagen, laminin, and proteoglycans that is extracted from a mouse sarcoma and used to mimic the extracellular environment a tumor cell encounters. PDAC cells, MIA PaCa-2 and PANC-1, were treated with PL (1 μM) or GEM (1 μM) alone or PL(1 μM) + GEM (1 μM), for 4 days. GEM alone was much less effective at reducing PDAC cell growth on Matrigel (μM range; Figure 1E) compared to traditional 2-D cultures (nM range; Figure 1A-1C), whereas PL showed a similar capacity to inhibit PDAC cell growth in the various growth environments. It was interesting to note a remarkable enhancement of the combination treatment in the growth-stressed Matrigel culture model compared to traditional culture model where the images clearly show that PL + GEM reduced both MIA PaCa-2 and PANC-1 cell growth as compared to control or single-agent treatments (Figure 1E). Therefore, the combination of PL + GEM allowed for significantly improved growth inhibition over single-agent GEM in the Matrigel assay. This is likely because PL works equally well in both growth environments, whereas GEM does not.

### PL enhances the *in vivo* therapeutic effects of GEM in an orthotopic mouse model

**Figure 2:**
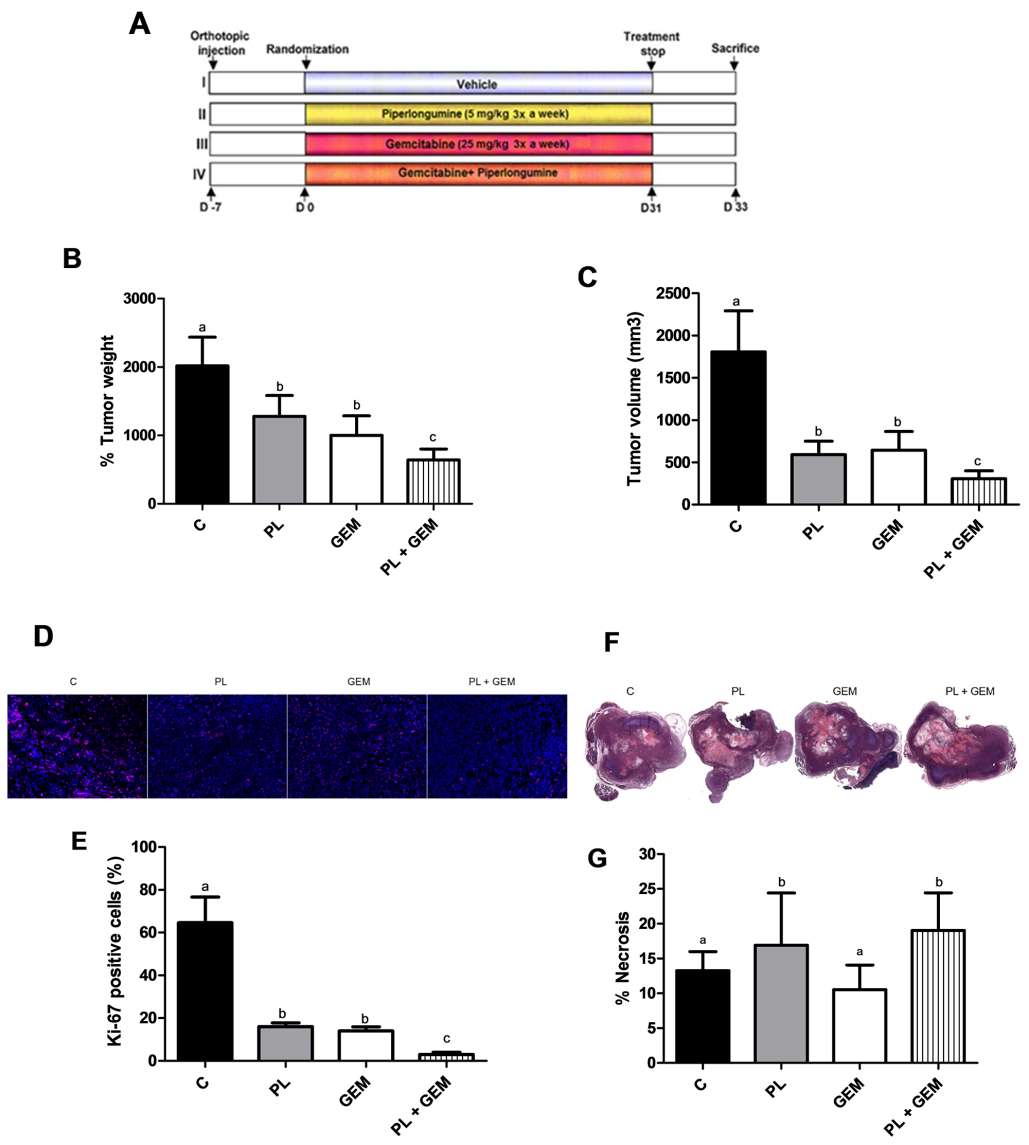
Effects of PL, GEM, and their combination on tumor growth, tumor cell proliferation, and tumor necrosis in mice implanted with MIA PaCa-2 cells **(A)** Schematic showing the study design. MIA PaCa-2 cells were injected into the pancreata of nude mice (n=12/treatment group). One week later, mice were randomized into one of four treatment groups (C, PL, GEM, or PL + GEM). Control mice received i.p. injections of the vehicle, PL mice received i.p. injections of 5 mg/kg PL, GEM mice received i.p. injections of 25 mg/kg GEM, and dual therapy mice received i.p. injections of 5 mg/kg PL and 25 mg/kg GEM three times a week for 31 days. **(B)** Tumor weight and **(C)**, tumor volume [V= (width)^2^ x length/2] were determined at the end of the study for each treatment group. Each value in the graph is the mean ± SE from 12 mice. **(D)** Tumor tissue sections from C, PL, GEM, or PL + GEM-treated mice were subjected to immunohistochemistry for Ki-67 staining. One representative image is shown for each treatment group. **(E)** The percentage of Ki-67 positive cells was determined for each treatment group by counting the number of Ki-67 stained cells compared to DAPI-stained cells. Each value in graph is the mean ± SE from 4-5 mice. **(F)** H&E staining of tumors was performed to identify viable pancreatic tissue compared to necrotic tissue. One representative image for each treatment group is shown. **(G)** The percentage of necrotic tissue was determined by subtracting the necrotic area from the total tissue section. Each value in graph is the mean ± SE from 4-5 mice. Treatments with bars that do not share a letter have differences that are statistically significant at P ≤ 0.05.

The promising results obtained from the Matrigel *in vitro* model evaluating PL + GEM prompted us to further explore this drug combination in an orthotopic animal model of PDAC. We evaluated the therapeutic advantage of combining PL + GEM in nude mice bearing orthotopically implanted MIA PaCa-2 cells. Doses of 5 mg/kg of PL and 25 mg/kg GEM were selected for intraperitoneal administration three times a week (Figure 2A) based on previously published reports [23–25]. The treatment efficacy was determined by considering the mean pancreatic tumor weight and volume immediately following euthanization. Administration of PL caused a 37% and 67% reduction in tumor weight and volume, respectively, compared to the control treatment (Figure 2B and 2C). GEM alone caused a 50% reduction in tumor weight and a 64% reduction in tumor volume compared to the control treatment. The combination of GEM and PL showed a significant decrease (p<0.01) in tumor weight (68%) and volume (83%) relative to the control, as well as to PL alone and GEM alone-treated mice. No macroscopic evidence of spreading to other visceral organs was evident for any experimental groups (not shown). Together, these results confirm the chemosensitizing effects of PL in an *in vivo* orthotopic PDAC model.

### Tumor cell proliferation is reduced by PL + GEM treatment of pancreatic tumors

Expression of the cell proliferation marker Ki-67 was investigated by immunohistochemistry in pancreatic tumors harvested from mice treated with the vehicle control, PL, GEM, or PL + GEM. Tumors obtained from PL and GEM alone-treated mice exhibited significantly fewer Ki-67-positive cells (16% and 14%, respectively) compared to control-treated mice which showed 65% Ki-67-positive cells (Figure 2D and 2E). Mice treated with PL + GEM displayed an even greater reduction in proliferating cells (3%) relative to control-treated mice (Figure 2D and 2E). These data provide additional evidence that PL + GEM prevents PDAC cells from proliferating *in vivo*.

### Necrosis is enhanced by PL + GEM treatment of pancreatic tumors

Hematoxylin and eosin staining was performed to determine the effect of PL, GEM, or their combination on pancreatic tumor necrosis (Figure 2F). Tumors obtained from PL-treated mice showed 17% necrotic area compared to control-treated mice (13%); whereas, GEM-treated mice showed 11% necrotic area (Figure 2G). Mice treated with PL + GEM had significantly greater amounts (19%) of necrotic tissue compared to control and GEM-treated mice. This result suggests that PL + GEM causes tumor cell death that would contribute to reduced tumor burden in mice.

### The combination of GEM and PL up-regulates p53 target genes and in pancreatic tumors

**Figure 3:**
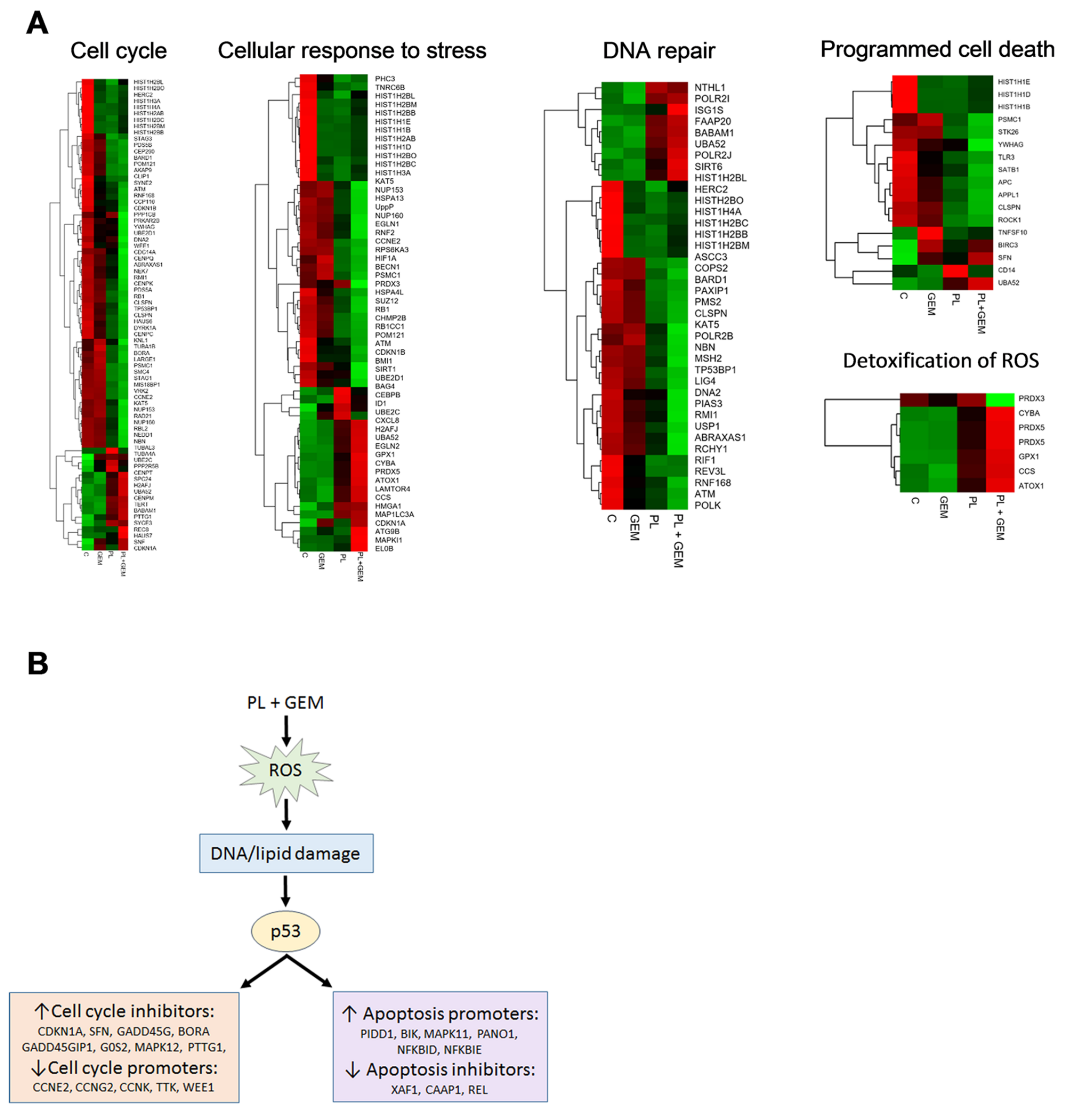
Effect of PL, GEM, and their combination on gene expression in orthotopic pancreas tumors and a proposed mechanism for their complementary anti-tumor effects RNA-Seq was performed on MIA PaCa-2 xenograft tumors obtained from C, GEM, PL, and PL + GEM-treated mice. **(A)** Heat maps for differentially expressed genes from five distinct pathways (cell cycle, cellular response to stress, DNA repair, programmed cell death, and detoxification of ROS) are shown for the four treatment groups (C, GEM, PL, and GEM + PL; n=3 each). The color gradient represents low (green) to high (red) levels of expression. **(B)** Proposed mechanism for the complementary anti-tumor effects of PL + GEM based on RNA-Seq data. The gene expression data indicate that PL + GEM alters the expression of a host of p53-responsive genes. PL + GEM increased a variety of cell cycle inhibitors and decreased cell cycle promoters. Further, PL + GEM increased apoptosis promoters and decreased apoptosis inhibitors.

To identify potential mechanisms associated with the complementary effects of PL + GEM, RNA-Seq was performed on tumors obtained from these mice. The transcriptome of control animals was compared to PL, GEM, and PL + GEM-treated mice to identify differentially expressed genes using a log2 fold-change cut off of ≤ −1 and ≥ 1. A total of 1,755 genes were differentially expressed in pancreatic tissues obtained from C vs. PL + GEM-treated mice compared to 25 and 715 genes for C vs. GEM or PL alone-treated mice, respectively. Out of 1,755 genes in the PL + GEM-treated tumors, 734 genes were upregulated and 1,021 genes were downregulated. Reactome software was used to identify the cellular and molecular processes involved in the antitumor effects of PL + GEM. The combination treatment modulated expression of genes involved in regulating cell death, cellular response to stress, the cell cycle, and DNA repair pathways (Figure 3A).

Ingenuity Pathway Analysis Software was used to identify the molecular pathways involved in the antitumor effects of PL + GEM. Seventy-five genes were up-regulated or down-regulated by PL + GEM treatment that were consistent with activation of p53 (activation z-score was 2.7). These included up-regulation of cell cycle-inhibitory genes (CDKN1A, SFN, GADD45G, GADD45GIP1, G0S2, MAPK12, PTTG1, and BORA) and down-regulation of cell cycle-promoting genes (CCNE2, CCNG2, CCNK, TTK, and WEE1). Further, apoptosis-promoting genes (PIDD1, BIK, MAPK11, PANO1, NFKBID, and NFKBIE) were up-regulated while apoptosis-inhibiting genes (XAF1, CAAP1, and REL) were down-regulated in PL + GEM-treated tumors (Figure 3B).

PL and GEM both induce oxidative stress; therefore, it was not surprising to observe over-expression of several oxidative stress response genes in the tumors of PL + GEM-treated mice. Detoxification enzymes, including GPX1, GPX4 CYBA, PRDX5, CCS, and ATOX1 were up-regulated in the combination treatment compared to controls (Figure 3B). Further, eleven different subunits of NADH:ubiquinone oxidoreductase (complex I), a major source of ROS in mitochondria, were significantly elevated in PL + GEM-treated tumors (Supplementary Data). Together, the RNA-Seq results lend support to the hypothesis that PL + GEM elevates ROS levels in tumor cells which leads to DNA damage and transcription of cell cycle arrest and apoptosis-associated genes (Figure 3B).

### The combination of PL and GEM increases ROS levels in PDAC cells compared to GEM alone

**Figure 4:**
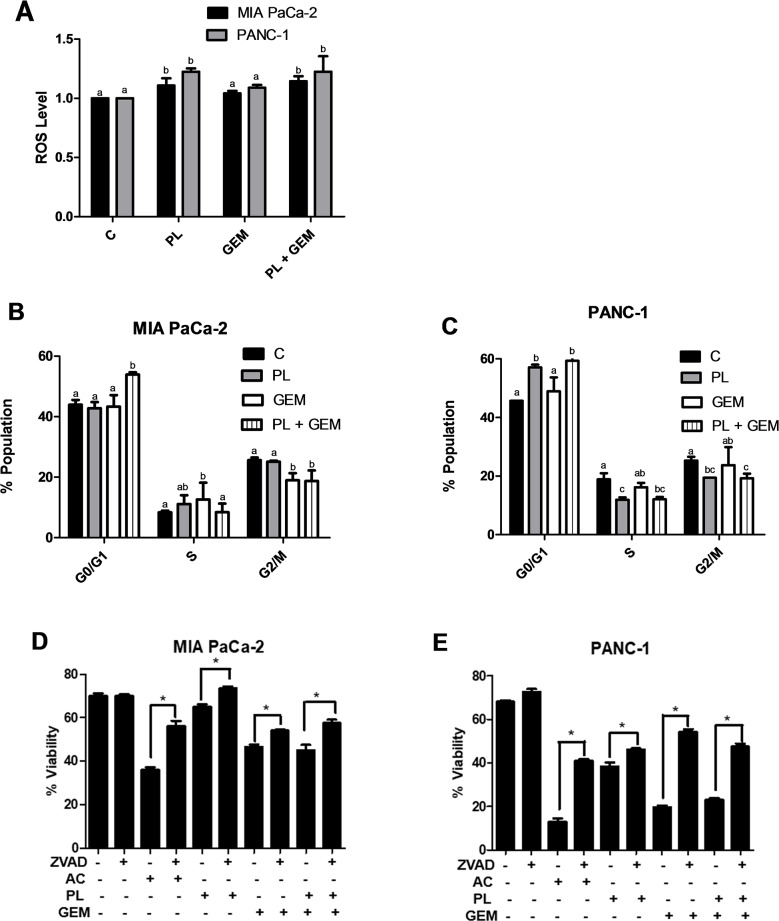
Effect of PL, GEM, and their combination on ROS levels, cell cycle profile, and viability **(A)** ROS levels were determined using DCFDA for MIA PaCa-2 and PANC-1 cells treated with vehicle control (C), PL (10 μM), GEM (10 μM), or PL (10 μM) + GEM (10 μM) for 1 h. Cell cycle profiling was determined using propidium iodide for **(B)**, MIA PaCa-2 and **(C)**, PANC-1 cells treated with vehicle control (C), PL (5 μM), GEM (100 nM), or PL (5 μM) + GEM (100 nM) for 24 h. The data shown in the bar graphs represent the average percent population in the given cell cycle phases ± SE for each treatment group. The experiment was conducted at least three times for each cell line. Treatments with bars that do not share a letter have differences that are statistically significant at P ≤ 0.05. A cell viability assay was performed for **(D)**, MIA PaCa-2 and **(E)**, PANC-1 cells treated with vehicle control (C), ZVAD (30 μM), actinomycin (AC; 1 μg/mL), PL (4 μM), GEM (0.5 μM), or their combinations for 48 h. ^*^ indicates a significant change in viability for a treatment group with respect to that treatment group combined with the caspase inhibitor ZVAD.

PL and GEM are both known to elevate ROS levels in cancer cells [9, 26], which are already under metabolic stress. Our RNA-Seq results directed us to evaluate if PL and GEM in combination could further enhance ROS levels. We postulated that the growth-inhibitory effects of combining these two agents could be a result of elevating ROS levels to a potentially lethal or near-lethal degree. To determine if PL + GEM could enhance ROS levels beyond either agent alone, we conducted an ROS assay in PDAC cells. PANC-1 cells were treated with PL (10 μM), GEM (10 μM), or PL + GEM (10 μM each) for 1 hr. The cells were stained with the fluorescent dye DCFDA to detect ROS, and fluorescence was determined using flow cytometry. PL + GEM significantly increased ROS levels compared to control and GEM, but not PL alone (Figure 4A). These results suggested the possibility that the combination of PL + GEM decreases PDAC cell growth by elevating ROS levels.

### PL + GEM induces arrest at G_0_/G_1_ phase of the cell cycle

Increased ROS levels can activate transcription factors and trigger changes in expression of cell cycle regulatory proteins that inhibit progression of cells through the cell cycle. Our RNA sequencing data revealed PL + GEM decreased expression of CCNE1 (cyclin E1) and increased expression of CDKN1A (p21), critical genes that help regulate the cell cycle. Therefore, we performed cell cycle profiling experiments to identify the effects of PL + GEM on PDAC cells. MIA PaCa-2 and PANC-1 cells were treated with PL (5 μM), GEM (100 nM), or PL (5 μM) + GEM (100 nM) for 24 h, after which cell cycle profiling was determined using PI staining and flow cytometry. Figure 4B and 4C show PL + GEM caused more cell cycle arrest at the G_0_/G_1_ phase in both PDAC cell lines compared to the control and individual treatments. A concomitant decrease in the G_2_/M population of cells was observed for PL + GEM-treated PDAC cells relative to control and individual treatment. These results suggest that arrest of the cell cycle in the G_0_/G_1_phase is a likely mechanism by which PL + GEM prevents PDAC cell proliferation.

### GEM, in combination with PL, reduces cell viability

Similarly, elevated ROS levels can trigger cell death by pushing cells over a toxicity threshold. Our RNA-Seq data (elevated caspase-9 and reduced NF-ĸB expression) showed that GEM in combination with PL affected the expression of apoptosis-related genes in a manner suggesting increased apoptosis. Therefore, we performed a viability assay for MIA PaCa-2 and PANC-1 cells treated with actinomycin D (positive control), PL (4 μM), GEM (0.5 μM), and PL (4 μM) + GEM (0.5 μM) with or without Z-VAD (caspase inhibitor) for 48 h. We found that PL + GEM caused a 44% and 25% decrease in PDAC cell viability compared to control-treated PANC-1 and MIA PaCa-2 cells, respectively (Figure 4D and 4E). Additionally, the caspase inhibitor Z-VAD blocked about 50% and 30% of PANC-1 and MIA PaCa-2 cell death, respectively. This result suggests that GEM in combination with PL caused apoptosis in a caspase-dependent manner for both PDAC cell lines. Interestingly, PANC-1 cells undergo more caspase-dependent cell death in response to PL + GEM than MIA PaCa-2 cells, which suggests that ROS-induced cell death mechanisms may differ based on PDAC subtype [27, 28].

## DISCUSSION

Effective treatment options are not currently available for advanced stage PDAC. GEM along with other adjuvants are used in combination to treat PDAC. However, these approaches have had limited success and only extend life span by months with added toxicity [5–7]. In this study, we determined that a small molecule obtained from a natural product enhances the effects of GEM in preclinical models of PDAC. We found that GEM in combination with PL significantly impaired stress-induced growth of PDAC cells *in vitro* and reduced tumor growth and weight compared to vehicle-control and to single-agent treatment in an orthotopic pancreatic mouse model. We propose that PL complements GEM's effect by increasing ROS levels, inducing DNA damage, and leading to cell cycle arrest and apoptosis.

Several combination therapies have been evaluated in clinical trials and are currently used for PDAC treatment, such as GEM with erlotinib, Cetuximab, or nab-paclitaxel; however, the outcomes have been disappointing [19, 21, 22, 23, 24]. In addition to dismal outcomes of chemotherapeutics, chemo-resistance and unwanted side effects in healthy tissues remain a problem. Recently, FOLFIRINOX, a cocktail of fluorouracil, leucovorin, irinotecan and oxaliplatin, has been widely used as a first line treatment for PDAC. While the overall survival for patients treated with FOLFIRINOX was 11.1 months compared to 6.8 months for the GEM-treated group, FOLFIRINOX causes significant side effects and is poorly tolerated [29].

Preclinical data suggest that ROS inducers are a promising therapeutic for treating various cancers, including PDAC [12, 30, 31]. We have previously shown that PL inhibits PANC-1 tumor progression in a subcutaneous model by inducing oxidative stress, DNA damage, and cell death [10]. Additionally, we have identified 683 gene transcripts significantly modulated by PL treatment in MIA PaCa-2 cells which included up-regulation of ER-stress and oxidative stress-associated genes [11]. The results from the experiments presented here build upon our previous observations, and provide a rationale for the use of a ROS-inducer such as PL in KRAS-mutant tumors like PDAC.

Previous studies have shown that combining GEM with an ROS inducer enhances PDAC cell death *in vitro* and *in vivo* [32, 33]. Here we show that the ROS inducer, PL, enhanced the therapeutic efficacy of GEM for two different PDAC cell lines (PANC-1 and MIA PaCa-2) that respond quite differently to GEM [18]. The three *in vitro* assays we used (MTT, clonogenic survival, and Matrigel growth) provided interesting results for the two PDAC cell lines which have similar genetic backgrounds (*KRAS*, *TP53*, *CDKN2A* mutant and *SMAD4* wild type) [20], but unique surface markers and metabolic profiles [21, 22]. MIA PaCa-2 cells are more glycolysic and mesencymal-like whereas PANC-1 cells are more lipogenic and epithelial-like. MIA PaCa-2 cells were more sensitive to GEM in the MTT assay than PANC-1, but not the clonogenic survival or Matrigel assays. When PANC-1 cells were seeded at low density or under the growth-stressed conditions of Matrigel, they showed similar sensitivities as MIA PaCa-2 to PL, GEM, and PL + GEM. The reasons for this might be due to differences in cell adhesion protein expression that would more greatly impact the growth of PANC-1 cells in low-density contexts than MIA PaCa-2, and the cells’ abilities to grow on different substrates [22] such as Matrigel.

In support of our *in vitro* results, we further showed that a PL (5 mg/kg, 3x weekly, i.p., 31 days) plus GEM (25 mg/kg, 3x weekly, i.p., 31 days) significantly reduced tumor size compared to control-treated or single agent-treated mice. Recently, PL + GEM was shown to induce apoptosis and inhibit BxPC-3 (KRAS wildtype, BRAF mutant) pancreatic tumor growth in a subcutaneous model [34]. This group found that PL (10 mg/kg, once daily, i.p, 24 days) enhanced the therapeutic efficacy of GEM (100 mg/kg, twice weekly, i.p, 24 days) for PDAC, lending additional support for the use of PL in combination with GEM to treat PDAC.

The anticancer mechanisms of PL have been evaluated in several different cancers, but are not well studied in PDAC. Previous reports have shown PL causes cell cycle arrest in the G_2_/M-phase via induction of GADD45A in gastric cancer cells and G_0_/G_1_-phase arrest via elevation of CDKN1A expression in oral squamous cancer cells [35, 36]. Our RNA sequencing data showed PL + GEM enhanced expression of p53-responsive genes including the cell cycle-associated genes CDKN1A, SFN, and GADD45G, and reduced expression of CCNE2, CCNG2, and CCNK in pancreatic tumors. Furthermore, our cell cycle profiling confirmed that cells treated with PL + GEM caused cell cycle arrest at the G_0_/G_1_ phase. In addition to causing cell cycle arrest, PL has been shown to induce cancer cell death through a variety of mechanisms including decreased DNA binding activity of the pro-survival transcription factor NF-κB in non-small cell lung cancer [37], activated MEK/ERK signaling in colon cancer cells [38, 39], elevated HO-1 via Nrf-2 signaling in breast cancer [40], and modulated JNK and PARP pathways in head and neck cancer [41]. Similarly, our RNA sequencing data showed PL decreased the expression of the NF-ĸB subunit transcript REL and increased the expression of the NF-ĸB inhibitors NFKBID and NFKBIE which could activate caspase-9 and cause apoptosis. Finally, we showed that PL + GEM caused cell death in PANC-1 cells and, to a lesser extent, MIA PaCa-2 cells through a caspase-dependent mechanism. Collectively, our results suggest that PL + GEM enhances ROS levels, which ultimately leads to cell cycle arrest and apoptosis.

Further evaluation of PL alone and in combination with GEM or other chemotherapies is needed to better understand the potential use of PL in a clinical setting. A limitation of the current study was the use of only one concentration of PL and GEM along with a single dosing schedule in the animal experiments. A more comprehensive study evaluating different doses and treatment schedules is needed. Further, it is not clear what effects PL + GEM might have on normal pancreatic cells. Given the selective toxicity of PL for cancer cells, it is possible that PL + GEM will not introduce additional toxicity to normal tissues, and this should be investigated. Further, it will be important to evaluate PL + GEM in the context of other animal models of PDAC including transgenic (immunocompetent) or patient derived xenografts which retain tumor architecture. Altogether, these results support PL's use as a promising complementary therapy to the anti-tumor effects of GEM in PDAC.

## MATERIALS AND METHODS

### Reagents

Piperlongumine (PL) was purchased from Indofine Chemical Company. Gemcitabine hydrochloride (GEM) was purchased from Sigma-Aldrich. PL and GEM were dissolved in 100% DMSO at stock concentrations of 100 mM and diluted in medium to working concentrations. Matrigel matrix was obtained from Corning. DCFDA was purchased from Life Technologies. Ki-67 antibody was purchased from Vector Labs. CF633-conjugated goat anti-mouse IgG secondary antibody was obtained from Biotium.

### Cell culture

PDAC cell lines (MIA PaCa-2 and PANC-1) were purchased from the American Type Culture Collection and grown at 37°C with 5% CO_2_. MIA PaCa-2 cells were cultured in DMEM high-glucose medium (Thermo Fisher Scientific) supplemented with 10% fetal bovine serum (Atlanta Biologicals) and 2.5% horse serum (Corning). PANC-1 cells were cultured in DMEM high-glucose medium supplemented with 10% fetal bovine serum. The cell lines were sub-cultured by enzymatic digestion with 0.25% trypsin/1 mM EDTA solution (Thermo Fisher Scientific) when they reached approximately 70% confluency.

### Cell viability assay

MIA PaCa-2 and PANC-1 cells (1,500 cells/well) were seeded in 96-well plates, and 48 h or 24 h later, respectively, were treated with different concentrations of PL (1 or 2 μM), GEM (1, 10, or 100 nM) or their combinations for 72 h. Drug concentrations were optimized for each cell-based assay. A cell viability assay was performed by adding 10 μl of 3-(4,5-dimethylthiazol-2-yl)-2,5-diphenyltetrazolium bromide (MTT) reagent to each well and incubating the plates for 2 h at 37°C. The MTT reagent was removed, DMSO (100 μL/well) was added to solubilize the crystals, and absorbance was measured at 570 nm. The data represent the average ± standard deviation for three independent experiments.

### Evaluation of synergistic effects by Jin's formula

The synergistic effects of combined PL and GEM were analyzed by Jin's formula. The formula is Q = (Ea+b)/((Ea + Eb) − (Ea x Eb)), where Ea+b, Ea, and Eb are the average inhibitory effects of PL + GEM, PL alone, and GEM alone, respectively. In this method, Q < 0.85 indicates antagonism, 0.85 < Q < 1.15 indicates additive effects, and Q > 1.15 indicates synergism. The Ea+b, Ea, and Eb values were obtained from MTT assays.

### Clonogenic survival assay

MIA PaCa-2 and PANC-1 cells (500 cells/dish) were seeded into 60 mm dishes. The next day, the cells were treated with PL (1 μM), GEM (1 nM), or PL (1 μM) + GEM (1 nM), for 10 days. After 10 days, the cells were fixed (methanol:acetic acid, 3:1) for 15 min. The fixing reagent was removed and the colonies were stained with 0.5% crystal violet (in methanol) for 15 min. Finally, the cells were washed with water, and colonies were counted manually to determine the surviving fraction. Representative images of the cell culture dishes show surviving colonies for each treatment group. Data shown represent the average ± standard deviation for the surviving cell fraction for three independent experiments for each cell line.

### Matrigel cell growth assay

Matrigel was thawed on ice overnight at 4°C. Matrigel (50 μL/well) was placed on the bottom of a 48-well plate and incubated at 37°C for 30 min. After incubation, MIA PaCa-2 or PANC-1 cells (4,000 cells/well) were seeded on top of the Matrigel layer. After 48 h, cells were treated with PL (1 μM), GEM (1 μM), or (1 μM) + GEM (1 μM). Images were taken 4 days after treatment. The experiment was repeated at least three times, and results shown represent one typical experiment for each cell line.

### Orthotopic mouse model of PDAC

Six- to 8-week-old female athymic nude mice (Nu/Nu) were purchased from Charles River Laboratories (Wilmington, MA). The mice were maintained in sterile conditions using the Innovive IVC System (Innovive), following a protocol approved by North Dakota State University's Institutional Animal Care and Use Committee. The mice were acclimated for 1 week before experimental manipulation. MIA PaCa-2 cells were harvested and resuspended in PBS. Mice were anesthetized with a ketamine-xylazine solution, a small left abdominal flank incision was made, and MIA PaCa-2 cells (~8 × 10^5^ in 30 μL) were injected into the pancreas using a 27-gauge needle. The abdomen was closed using a 2-layer suture technique involving chromic catgut and ethilon sutures. Two weeks after cancer cell implantation, the mice were randomized into 4 groups and treated i.p. 3x/week as follows: (i) untreated control (DMSO 5% v/v), (ii) PL (5 mg/kg body weight), (iii) GEM (25 mg/kg), and (iv) PL + GEM (5 mg/kg and 25 mg/kg, respectively). PL was dissolved in DMSO at a stock concentration of 9.9 mg/ml and further diluted in PBS before administering to the mice. The final concentration of DMSO in the PL working solution was 5% v/v. GEM was dissolved in sterile saline at a stock concentration of 50 g/L. Each treatment group contained 12 animals. After 4 weeks of treatment, the animals were euthanized by an overdose of ketamine-xylazine solution followed by cervical dislocation. The primary tumors in the pancreata were excised and measured for tumor weight and volume [V= (width)^2^ x length/2]. The tumor weight and volume were compared between groups using an unpaired Student's *t*-test. Tumors from half of the mice for each treatment were paraformaldehyde-fixed and paraffin-embedded for immunohistochemistry. The other half of the mouse tumors were snap-frozen in liquid nitrogen and stored at −80°C.

### Immunohistochemistry and tissue histology

Tumor tissues from control, PL, GEM, and PL + GEM-treated mice were collected and fixed for 24 h in formaldehyde. Paraffin-embedded 5 μm thick sections of tumor tissues were prepared. Sections were deparaffinized with Histo-Clear and ethanol, followed by antigen retrieval in 10 mM sodium citrate buffer (0.05% Tween 20, pH 6.0) using an autoclave method. The sections were blocked for 20 min in blocking buffer (10% normal goat serum in TBST) and incubated with Ki-67 (1:100) or overnight at 4°C. The next day, sections were incubated with CF633-conjugated goat anti-mouse secondary antibody (1:250) for 1 h at room temperature. For H&E staining, tissue slides were rehydrated, stained with hematoxylin for 5 min, washed with distilled water, soaked in 95% ethanol for 30 sec, stained with eosin for 1 min, dehydrated with 100% ethanol for 1 min, and washed in xylene. After mounting a coverslip using Hardset Mounting Medium with DAPI (Vector Labs, Burlingame, CA), slides were visualized using a Zeiss inverted Axio Observer Z1 microscope. The percentage of Ki-67-positive cells was measured based on the number of pink-stained cells relative to the number of blue DAPI-stained nuclei. The percentage of necrotic cells was determined based on the area of light pink subtracted from total area on H & E-stained sections.

### RNA extraction

Total RNA was extracted from pancreatic tumors from each group using TRI Reagent (Life Technologies). Briefly, 5 mg of tissue was homogenized in 1 mL of TRI Reagent and incubated at room temperature for 15 min. Next, 50 μL of BCP was added. The tube was shaken vigorously for 30 s, incubated again at room temperature for 3 min, and then centrifuged at 12,000 x g for 15 min at 4°C. The supernatant was transferred to a fresh tube, and total RNA was precipitated by adding 1.5 volumes of 100% ethanol. The precipitated RNA sample was transferred to an RNeasy Mini spin column (Qiagen), and RNA purification was performed using the manufacturer's protocol.

### RNA-Sequencing

Three micrograms of total RNA from three mice in each group: control, PL, GEM, and PL + GEM were sent for sequencing at the University of Minnesota Genomics Center. Six barcoded libraries were created and sequenced on an Illumina HiSeq 2500. Single-end reads of 50 base pairs (bp) were obtained and mapped to the *Mus musculus* genome (Build: Mus_musculus.GRCm38) downloaded from Ensembl. The raw fastq data were subjected to quality trimming using Sickle, for a minimum length of 50. HISAT2 [13] was used to map the reads on the genome. SAMtools [14] and BAMtools [15] were used to convert and sort the BAM files. HTSeq [16] was used to count the reads.

### Cell cycle arrest assay

Briefly, PDAC cell lines (MIA PaCa-2 and PANC-1) were seeded in 6-well plates and incubated overnight. Cells were then synchronized overnight using serum free medium. Next, cells were treated with PL (5 μM), GEM (100 nM), or 5 μM PL + 100 nM GEM for 24 h. Cells were then harvested by trypsinization, washed, and re-suspended in 70% ethanol overnight at 4°C. Finally, cells were re-suspended in PBS containing 50 μg/mL propidium iodide (PI) and 1 μg/mL of RNase A. Flow cytometry was performed to determine the percentage of cells in each phase of the cell cycle.

### Viability assay

A viability assay was performed using PI staining followed by flow cytometry. Briefly, PDAC cell lines (MIA PaCa-2 and PANC-1) were seeded in 6-well plates and incubated overnight. Cells were then treated with and without Z-VAD (25 μM) for 30 min at 37°C. Next, cells were treated with actinomycin D (1 μg/ml), PL (4 μM), GEM (0.5 μM), or PL + GEM (4 μM + 0.5 μM, respectively) for 48 h. Cells were then harvested by trypsinization, washed, and re-suspended in PBS containing 1 μg/mL of PI. Flow cytometry was performed to determine the percentage of PI-positive cells as an indication of overall viability.

### Measurement of ROS levels by the 2,7-dichlorodihydrofluorescein diacetate (DCFDA) assay

MIA PaCa-2 and PANC-1 cells (5.0 × 10^5^ cells/ml) were suspended in culture medium and treated with PL (10 μM), GEM (10 μM), or their combinations (10 μM PL + 10 μM GEM) for 1 h. After treatment, cells were harvested by centrifugation and re-suspended in 20 μM DCFDA in PBS. The cells were incubated at 37°C for 30 min before flow cytometric analysis using an Accuri C6 Flow Cytometer. Three technical replicates were included for each experiment, and the experiments were performed in biological triplicates for each cell line.

### Statistical analyses

Statistical analyses were performed in SAS version 9.4 using the PROC MIXED procedure. Analysis of variance (ANOVA) was conducted for each analysis. All analyses included “treatment” as the main fixed effect in the ANOVA model. For experiments conducted over time, “time” was included in the model as a fixed effect. For analysis of data that involved experiments repeated independently, “experiment” was included in the model as a blocking effect. Finally, for the MTT assay fold change analysis, “plate” was treated as a random effect. Post-hoc *t*-tests were performed to compare each pair of treatments. Differences were considered statistically significant for P < 0.05.

## SUPPLEMENTARY MATERIALS TABLE





## Abbreviations

MTT: 3-(4, 5-dimethylthiazol-2-yl)-2, 5-diphenyltetra zolium bromide
GEM: gemcitabine
PDAC: pancreatic ductal adenocarcinoma
PL: piperlongumine
PI: propidium iodide
ROS: reactive oxygen species
RNA-Seq: RNA sequencing
