# Supplementary material for: Piperlongumine potentiates the effects of gemcitabine in in vitro and in vivo human pancreatic cancer models
Source: Oncotarget. 2017 Dec 23;9(12):10457–69. doi: 10.18632/oncotarget.23623 (PMC5828188; doi:10.18632/oncotarget.23623)
Supplement: Supplementary file 1 [file oncotarget-09-10457-s001.pdf]

Piperlongumine potentiates the effects of gemcitabine in *in vitro* and *in vivo* human pancreatic cancer models

SUPPLEMENTARY MATERIALS

Supplementary Table 1: Complete results of differentially expressed genes detected by RNA-Seq for PL + GEM-treated tumors compared to control.

See Supplementary File 1
