# Supplementary material for: Piperlongumine potentiates the effects of gemcitabine in in vitro and in vivo human pancreatic cancer models
Source: Oncotarget. 2017 Dec 23;9(12):10457–69. doi: 10.18632/oncotarget.23623 (PMC5828188; doi:10.18632/oncotarget.23623)
Supplement: Supplementary file 2 [file oncotarget-09-10457-s002.docx]

| **Ensembl ID** | **Description** | **Log2FoldChange** | **P-value** |
| --- | --- | --- | --- |
| ENSG00000197565 | collagen type IV alpha 6 chain | 6.8811999 | 0.001021726 |
| ENSG00000153292 | adhesion G protein-coupled receptor F1 | 6.707976949 | 0.000410611 |
| ENSG00000171495 | maestro heat like repeat family member 2B | 6.360869942 | 0.004953408 |
| ENSG00000120949 | TNF receptor superfamily member 8 | 5.780287701 | 1.85E-05 |
| ENSG00000149968 | matrix metallopeptidase 3 | 5.517778693 | 0.029381777 |
| ENSG00000254647 | insulin | 5.353773539 | 0.004080413 |
| ENSG00000091128 | laminin subunit beta 4 | 5.170630935 | 0.001213264 |
| ENSG00000198417 | metallothionein 1F | 4.90969975 | 0.000377751 |
| ENSG00000261371 | platelet and endothelial cell adhesion molecule 1 | 4.88750207 | 0.004713698 |
| ENSG00000172551 | mucin like 1 | 4.684445478 | 0.030695945 |
| ENSG00000145681 | hyaluronan and proteoglycan link protein 1 | 4.548190741 | 0.024877238 |
| ENSG00000197253 | tryptase beta 2 (gene/pseudogene) | 4.414509317 | 0.005480724 |
| ENSG00000167656 | lymphocyte antigen 6 complex, locus D | 4.406895027 | 0.001459452 |
| ENSG00000182557 | sphingolipid transporter 3 (putative) | 3.974760122 | 0.00084206 |
| ENSG00000133083 | doublecortin like kinase 1 | 3.860213704 | 0.016413228 |
| ENSG00000166670 | matrix metallopeptidase 10 | 3.796622909 | 0.007095944 |
| ENSG00000074771 | NADPH oxidase 3 | 3.709531528 | 0.017694427 |
| ENSG00000146530 | von Willebrand factor D and EGF domains | 3.617940266 | 0.015624215 |
| ENSG00000177675 | CD163 molecule like 1 | 3.602885112 | 0.000289891 |
| ENSG00000160932 | lymphocyte antigen 6 complex, locus E | 3.400651044 | 0.000959812 |
| ENSG00000182836 | phosphatidylinositol specific phospholipase C X domain containing 3 | 3.39436717 | 0.002002489 |
| ENSG00000163121 | neuralized E3 ubiquitin protein ligase 3 | 3.240505748 | 0.007687924 |
| ENSG00000168269 | forkhead box I1 | 3.203275198 | 0.006892187 |
| ENSG00000277117 |  | 3.111787534 | 0.000405237 |
| ENSG00000112299 | vanin 1 | 3.108028316 | 0.018523539 |
| ENSG00000172824 | carboxylesterase 4A | 3.101567375 | 0.008134192 |
| ENSG00000181577 | chromosome 6 open reading frame 223 | 3.085127131 | 0.014017181 |
| ENSG00000205809 | killer cell lectin like receptor C2 | 3.082465916 | 0.01031829 |
| ENSG00000102962 | C-C motif chemokine ligand 22 | 3.065251481 | 0.002981462 |
| ENSG00000133055 | myosin binding protein H | 3.065094899 | 0.013540255 |
| ENSG00000136352 | NK2 homeobox 1 | 3.030067229 | 0.012817588 |
| ENSG00000126583 | protein kinase C gamma | 2.856883479 | 4.89E-05 |
| ENSG00000128422 | keratin 17 | 2.84238443 | 0.0060147 |
| ENSG00000100055 | cytohesin 4 | 2.811897156 | 0.000541834 |
| ENSG00000197467 | collagen type XIII alpha 1 chain | 2.745606549 | 0.002362449 |
| ENSG00000121101 | testis expressed 14, intercellular bridge forming factor | 2.732554556 | 0.027230098 |
| ENSG00000152766 | ankyrin repeat domain 22 | 2.722704289 | 1.02E-06 |
| ENSG00000154269 | ectonucleotide pyrophosphatase/phosphodiesterase 3 | 2.682736692 | 0.000432669 |
| ENSG00000108759 | keratin 32 | 2.674299358 | 4.68E-05 |
| ENSG00000163637 | prickle planar cell polarity protein 2 | 2.638902725 | 0.005667282 |
| ENSG00000000971 | complement factor H | 2.633678791 | 0.021251949 |
| ENSG00000125910 | sphingosine-1-phosphate receptor 4 | 2.632208863 | 0.024044752 |
| ENSG00000125850 | ovo like zinc finger 2 | 2.600341024 | 0.003132771 |
| ENSG00000057294 | plakophilin 2 | 2.579549401 | 0.025774784 |
| ENSG00000106852 | LIM homeobox 6 | 2.557087232 | 0.018668805 |
| ENSG00000137975 | chloride channel accessory 2 | 2.546301763 | 0.026630189 |
| ENSG00000183971 | neuropeptide W | 2.528704701 | 0.00011285 |
| ENSG00000171695 | LKAAEAR motif containing 1 | 2.518591361 | 0.004555785 |
| ENSG00000105427 | cornifelin | 2.517690308 | 2.45E-05 |
| ENSG00000196748 | colipase like 2 | 2.515295311 | 0.000277979 |
| ENSG00000125895 | transmembrane protein 74B | 2.483137165 | 0.00013328 |
| ENSG00000138083 | SIX homeobox 3 | 2.48307536 | 0.008139633 |
| ENSG00000130222 | growth arrest and DNA damage inducible gamma | 2.477673699 | 4.21E-06 |
| ENSG00000100626 | polypeptide N-acetylgalactosaminyltransferase 16 | 2.474438032 | 0.001683141 |
| ENSG00000223572 | creatine kinase, mitochondrial 1A | 2.472749131 | 8.57E-05 |
| ENSG00000140986 | ribosomal protein L3 like | 2.472474873 | 0.004438386 |
| ENSG00000156113 | potassium calcium-activated channel subfamily M alpha 1 | 2.456532873 | 0.022578058 |
| ENSG00000138166 | dual specificity phosphatase 5 | 2.443923021 | 0.010052002 |
| ENSG00000225950 | neurotrophin 4 | 2.430245319 | 7.11E-05 |
| ENSG00000134668 | SPOC domain containing 1 | 2.424214888 | 0.003932957 |
| ENSG00000084636 | collagen type XVI alpha 1 chain | 2.358662093 | 0.000208601 |
| ENSG00000118523 | connective tissue growth factor | 2.351710419 | 3.89E-10 |
| ENSG00000164741 | DLC1 Rho GTPase activating protein | 2.340399802 | 0.004405594 |
| ENSG00000181652 | autophagy related 9B | 2.338327249 | 0.001779703 |
| ENSG00000059377 | thromboxane A synthase 1 | 2.334073726 | 0.000202404 |
| ENSG00000196337 | chorionic gonadotropin beta subunit 7 | 2.331312939 | 0.001728338 |
| ENSG00000160318 | claudin domain containing 2 | 2.330730799 | 1.30E-05 |
| ENSG00000183615 | family with sequence similarity 167 member B | 2.314010994 | 3.38E-06 |
| ENSG00000237289 | creatine kinase, mitochondrial 1B | 2.282697747 | 0.002481369 |
| ENSG00000175877 | Williams-Beuren syndrome chromosome region 28 | 2.277099316 | 0.004949262 |
| ENSG00000153789 | family with sequence similarity 92 member B | 2.270842531 | 0.023233947 |
| ENSG00000149418 | suppression of tumorigenicity 14 | 2.257271088 | 0.004108722 |
| ENSG00000188064 | Wnt family member 7B | 2.252733302 | 0.002537367 |
| ENSG00000220201 | zinc finger, GATA-like protein 1 | 2.245691765 | 0.001000464 |
| ENSG00000123689 | G0/G1 switch 2 | 2.234309142 | 0.026684095 |
| ENSG00000184560 | chromosome 17 open reading frame 74 | 2.234059443 | 0.014845255 |
| ENSG00000135447 | protein phosphatase 1 regulatory inhibitor subunit 1A | 2.229653022 | 0.02142632 |
| ENSG00000130487 | kelch domain containing 7B 25145] | 2.220170348 | 0.031607767 |
| ENSG00000141753 | insulin like growth factor binding protein 4 | 2.201388999 | 0.004274552 |
| ENSG00000145920 | complexin 2 | 2.18116277 | 0.009943281 |
| ENSG00000100968 | nuclear factor of activated T-cells 4 | 2.179866996 | 0.02295982 |
| ENSG00000100060 | MFNG O-fucosylpeptide 3-beta-N-acetylglucosaminyltransferase | 2.163516488 | 1.23E-05 |
| ENSG00000153162 | bone morphogenetic protein 6 | 2.16264299 | 0.023789631 |
| ENSG00000149043 | synaptotagmin 8 | 2.157884232 | 7.10E-08 |
| ENSG00000168071 | coiled-coil domain containing 88B | 2.157316004 | 1.44E-09 |
| ENSG00000188290 | hes family bHLH transcription factor 4 | 2.147925163 | 0.002809703 |
| ENSG00000172638 | EGF containing fibulin like extracellular matrix protein 2 | 2.146917285 | 1.58E-09 |
| ENSG00000140678 | integrin subunit alpha X | 2.128808131 | 0.017217782 |
| ENSG00000100234 | TIMP metallopeptidase inhibitor 3 | 2.118806889 | 2.30E-05 |
| ENSG00000146966 | DENN domain containing 2A | 2.084933028 | 0.029939439 |
| ENSG00000197594 | ectonucleotide pyrophosphatase/phosphodiesterase 1 | 2.08155983 | 0.001371592 |
| ENSG00000182931 | WAP four-disulfide core domain 10B | 2.07345549 | 0.003335419 |
| ENSG00000163545 | NUAK family kinase 2 | 2.068917298 | 0.003982857 |
| ENSG00000112769 | laminin subunit alpha 4 | 2.060990815 | 0.023755479 |
| ENSG00000100290 | BCL2 interacting killer | 2.05269359 | 1.78E-08 |
| ENSG00000269881 |  | 2.050249573 | 0.016860503 |
| ENSG00000130208 | apolipoprotein C1 | 2.047472099 | 1.95E-15 |
| ENSG00000160282 | formimidoyltransferase cyclodeaminase | 2.045188175 | 0.009170458 |
| ENSG00000135114 | 2'-5'-oligoadenylate synthetase like | 2.042728024 | 7.30E-08 |
| ENSG00000113070 | heparin binding EGF like growth factor | 2.026314263 | 0.00996553 |
| ENSG00000177989 | outer dense fiber of sperm tails 3B | 2.023107404 | 4.49E-06 |
| ENSG00000105246 | Epstein-Barr virus induced 3 | 2.021091806 | 0.033863616 |
| ENSG00000124762 | cyclin dependent kinase inhibitor 1A | 2.011410491 | 0.027025357 |
| ENSG00000122861 | plasminogen activator, urokinase | 2.007850673 | 1.53E-05 |
| ENSG00000187186 |  | 1.992650511 | 0.01604535 |
| ENSG00000104808 | dihydrodiol dehydrogenase | 1.991420578 | 0.00313494 |
| ENSG00000175793 | stratifin | 1.974918219 | 0.006196995 |
| ENSG00000169403 | platelet activating factor receptor | 1.973957224 | 0.016216854 |
| ENSG00000167772 | angiopoietin like 4 | 1.957443475 | 0.000297202 |
| ENSG00000261857 | melanoma inhibitory activity | 1.933975045 | 0.016910413 |
| ENSG00000106003 | LFNG O-fucosylpeptide 3-beta-N-acetylglucosaminyltransferase | 1.926022364 | 5.76E-07 |
| ENSG00000185499 | mucin 1, cell surface associated | 1.915641136 | 5.17E-05 |
| ENSG00000103355 | protease, serine 33 | 1.909508064 | 0.018606554 |
| ENSG00000221887 | histocompatibility minor serpin domain containing | 1.902846151 | 0.018018345 |
| ENSG00000023171 | GRAM domain containing 1B | 1.897562892 | 0.002298869 |
| ENSG00000162552 | Wnt family member 4 | 1.895448889 | 0.005476784 |
| ENSG00000177098 | sodium voltage-gated channel beta subunit 4 | 1.892782966 | 0.009573576 |
| ENSG00000121966 | C-X-C motif chemokine receptor 4 | 1.892438375 | 0.005707459 |
| ENSG00000178821 | transmembrane protein 52 | 1.885277619 | 7.17E-06 |
| ENSG00000130598 | troponin I2, fast skeletal type | 1.865902191 | 0.00011457 |
| ENSG00000198598 | matrix metallopeptidase 17 | 1.865562668 | 0.00349203 |
| ENSG00000184925 | lipocalin 12 | 1.863912648 | 0.002656272 |
| ENSG00000131650 | kringle containing transmembrane protein 2 | 1.858214841 | 2.96E-07 |
| ENSG00000130751 | neuronal PAS domain protein 1 | 1.851265848 | 1.19E-05 |
| ENSG00000018280 | solute carrier family 11 member 1 | 1.850288431 | 0.010300031 |
| ENSG00000123342 | matrix metallopeptidase 19 | 1.846364917 | 0.002596607 |
| ENSG00000101443 | WAP four-disulfide core domain 2 | 1.840812998 | 0.003879673 |
| ENSG00000126561 | signal transducer and activator of transcription 5A | 1.839083801 | 0.035311463 |
| ENSG00000158458 | neuregulin 2 | 1.83411744 | 0.030952319 |
| ENSG00000088836 | solute carrier family 4 member 11 | 1.827522237 | 7.57E-08 |
| ENSG00000149294 | neural cell adhesion molecule 1 | 1.822394484 | 0.005362642 |
| ENSG00000078081 | lysosomal associated membrane protein 3 | 1.817977357 | 0.022478769 |
| ENSG00000222047 | chromosome 10 open reading frame 55 | 1.814062442 | 0.000531433 |
| ENSG00000164362 | telomerase reverse transcriptase | 1.809892191 | 0.014320547 |
| ENSG00000087085 | acetylcholinesterase (Cartwright blood group) | 1.806384609 | 0.00678416 |
| ENSG00000185664 | premelanosome protein | 1.799929195 | 0.013593002 |
| ENSG00000205277 | mucin 12, cell surface associated | 1.798008208 | 3.70E-05 |
| ENSG00000132481 | tripartite motif containing 47 | 1.795135567 | 0.000433193 |
| ENSG00000125505 | membrane bound O-acyltransferase domain containing 7 | 1.79444132 | 0.001100131 |
| ENSG00000116667 | chromosome 1 open reading frame 21 | 1.790157217 | 0.001081772 |
| ENSG00000205639 | major facilitator superfamily domain containing 2B | 1.789313417 | 0.00015041 |
| ENSG00000130203 | apolipoprotein E | 1.777436954 | 0.00242972 |
| ENSG00000101331 | CCM2 like scaffolding protein | 1.771033827 | 0.009723688 |
| ENSG00000167549 | coronin 6 | 1.766247921 | 0.002522708 |
| ENSG00000164379 | forkhead box Q1 | 1.754873267 | 0.014404096 |
| ENSG00000104522 | tissue specific transplantation antigen P35B | 1.750446684 | 0.01954151 |
| ENSG00000260428 | scleraxis bHLH transcription factor | 1.745581628 | 0.00448811 |
| ENSG00000126460 | proline rich and Gla domain 2 | 1.742942775 | 0.004581922 |
| ENSG00000243449 | chromosome 4 open reading frame 48 | 1.741729069 | 1.39E-05 |
| ENSG00000166289 | pleckstrin homology and FYVE domain containing 1 | 1.736110136 | 2.86E-07 |
| ENSG00000176170 | sphingosine kinase 1 | 1.727372788 | 0.002310264 |
| ENSG00000213397 | HAUS augmin like complex subunit 7 | 1.722290853 | 0.000245835 |
| ENSG00000178038 | ALS2 C-terminal like | 1.703959246 | 0.005044052 |
| ENSG00000106089 | syntaxin 1A | 1.700807094 | 0.000167721 |
| ENSG00000167945 | proline rich 25 | 1.700529219 | 0.009248478 |
| ENSG00000102032 | renin binding protein | 1.700421962 | 0.002955429 |
| ENSG00000169583 | chloride intracellular channel 3 | 1.700389068 | 0.030547029 |
| ENSG00000143590 | ephrin A3 | 1.695085145 | 1.95E-06 |
| ENSG00000099812 | mitotic spindle positioning | 1.68695515 | 1.05E-05 |
| ENSG00000128564 | VGF nerve growth factor inducible | 1.679279885 | 0.00355397 |
| ENSG00000157343 | armadillo repeat containing 12 | 1.674832907 | 0.004841563 |
| ENSG00000068831 | RAS guanyl releasing protein 2 | 1.673936016 | 0.031335697 |
| ENSG00000127129 | endothelin 2 | 1.670121066 | 0.00411118 |
| ENSG00000165171 | Williams Beuren syndrome chromosome region 27 | 1.670057683 | 1.40E-06 |
| ENSG00000230601 |  | 1.669220601 | 0.001706925 |
| ENSG00000212123 | proline rich 22 | 1.668222722 | 0.002412372 |
| ENSG00000130649 | cytochrome P450 family 2 subfamily E member 1 | 1.6672403 | 0.030601666 |
| ENSG00000278817 |  | 1.659057419 | 0.014259786 |
| ENSG00000163462 | tripartite motif containing 46 | 1.646544501 | 0.006636295 |
| ENSG00000174521 | tetratricopeptide repeat domain 9B | 1.643773718 | 0.006684387 |
| ENSG00000233493 | transmembrane protein 238 | 1.642771061 | 4.10E-07 |
| ENSG00000155367 | protein phosphatase, Mg2+/Mn2+ dependent 1J | 1.637083892 | 0.013616861 |
| ENSG00000140481 | coiled-coil domain containing 33 | 1.634001132 | 9.08E-06 |
| ENSG00000196739 | collagen type XXVII alpha 1 chain | 1.628697633 | 0.000781072 |
| ENSG00000167644 | chromosome 19 open reading frame 33 | 1.627790181 | 0.000616561 |
| ENSG00000142694 | eva-1 homolog B | 1.61958698 | 0.000203485 |
| ENSG00000173267 | synuclein gamma | 1.614138632 | 0.031169981 |
| ENSG00000274897 | proapoptotic nucleolar protein 1 | 1.607859006 | 0.000635245 |
| ENSG00000197696 | neuromedin B | 1.605570705 | 1.03E-05 |
| ENSG00000185338 | suppressor of cytokine signaling 1 | 1.603243704 | 0.001213651 |
| ENSG00000001617 | semaphorin 3F | 1.599804219 | 0.000193805 |
| ENSG00000148926 | adrenomedullin | 1.598257137 | 0.016544694 |
| ENSG00000187800 | platelet endothelial aggregation receptor 1 | 1.598129486 | 0.009984417 |
| ENSG00000239282 | GATS protein like 3 | 1.596415901 | 0.004219912 |
| ENSG00000124614 | ribosomal protein S10 | 1.588145472 | 5.99E-05 |
| ENSG00000142684 | zinc finger protein 593 | 1.586291558 | 0.00191743 |
| ENSG00000106070 | growth factor receptor bound protein 10 | 1.582710899 | 0.004361474 |
| ENSG00000217442 | synaptonemal complex central element protein 3 | 1.577433823 | 0.019717681 |
| ENSG00000158747 | neuroblastoma 1, DAN family BMP antagonist | 1.572735586 | 3.44E-05 |
| ENSG00000100097 | galectin 1 | 1.562273583 | 1.30E-05 |
| ENSG00000111879 | family with sequence similarity 184 member A | 1.562212383 | 0.017213463 |
| ENSG00000204397 | caspase recruitment domain family member 16 | 1.555254274 | 0.000618194 |
| ENSG00000173338 | potassium two pore domain channel subfamily K member 7 | 1.554167566 | 0.030335425 |
| ENSG00000013588 | G protein-coupled receptor class C group 5 member A | 1.55095187 | 0.000504645 |
| ENSG00000142959 | bestrophin 4 | 1.548849767 | 0.016250279 |
| ENSG00000228049 | RNA polymerase II subunit J2 | 1.539629805 | 0.022466349 |
| ENSG00000167397 | vitamin K epoxide reductase complex subunit 1 | 1.539361016 | 1.35E-05 |
| ENSG00000142408 | calcium voltage-gated channel auxiliary subunit gamma 8 | 1.537511078 | 0.010960275 |
| ENSG00000111424 | vitamin D (1,25- dihydroxyvitamin D3) receptor | 1.53713443 | 0.01104731 |
| ENSG00000159753 | capping protein regulator and myosin 1 linker 2 | 1.533189378 | 0.002076859 |
| ENSG00000079156 | oxysterol binding protein like 6 | 1.53179903 | 0.017531173 |
| ENSG00000165644 | catechol-O-methyltransferase domain containing 1 | 1.530986058 | 1.21E-09 |
| ENSG00000170545 | small cell adhesion glycoprotein | 1.530059309 | 5.74E-06 |
| ENSG00000166592 | RRAD, Ras related glycolysis inhibitor and calcium channel regulator | 1.529457233 | 0.012659241 |
| ENSG00000176125 | UFM1 specific peptidase 1 (inactive) | 1.522576852 | 1.63E-05 |
| ENSG00000198353 | homeobox C4 | 1.522015632 | 0.015472073 |
| ENSG00000100593 | isthmin 2 | 1.521593173 | 0.003343001 |
| ENSG00000184292 | tumor-associated calcium signal transducer 2 | 1.51985322 | 0.012779398 |
| ENSG00000140511 | hyaluronan and proteoglycan link protein 3 | 1.519331506 | 0.006258232 |
| ENSG00000189410 | SH2 domain containing 5 | 1.518302951 | 0.00588941 |
| ENSG00000149564 | endothelial cell adhesion molecule | 1.517367765 | 0.001283109 |
| ENSG00000131477 | receptor activity modifying protein 2 | 1.517184431 | 0.002692117 |
| ENSG00000112320 | sine oculis binding protein homolog | 1.513701461 | 0.003169682 |
| ENSG00000176919 | complement C8 gamma chain | 1.513607344 | 0.017908812 |
| ENSG00000213398 | lecithin-cholesterol acyltransferase | 1.509739476 | 0.001346187 |
| ENSG00000131187 | coagulation factor XII | 1.509566865 | 9.45E-07 |
| ENSG00000074317 | synuclein beta | 1.507850393 | 0.019725923 |
| ENSG00000205436 | exocyst complex component 3 like 4 | 1.507677624 | 0.015252646 |
| ENSG00000212864 | ring finger protein 208 | 1.507356388 | 7.89E-05 |
| ENSG00000182580 | EPH receptor B3 | 1.507178136 | 0.011991472 |
| ENSG00000142765 | synaptotagmin like 1 | 1.500907054 | 4.66E-08 |
| ENSG00000006210 | C-X3-C motif chemokine ligand 1 | 1.500321875 | 0.000595843 |
| ENSG00000189280 | gap junction protein beta 5 | 1.499790115 | 0.006350649 |
| ENSG00000149489 | retinal outer segment membrane protein 1 | 1.497846778 | 1.15E-06 |
| ENSG00000128340 | ras-related C3 botulinum toxin substrate 2 (rho family, small GTP binding protein Rac2) | 1.495348883 | 0.034576116 |
| ENSG00000132563 | receptor accessory protein 2 | 1.495051512 | 5.03E-06 |
| ENSG00000259305 | ZHX1-C8orf76 readthrough | 1.494979966 | 0.024944607 |
| ENSG00000262165 |  | 1.490049758 | 0.001763326 |
| ENSG00000248712 | coiled-coil domain containing 153 | 1.489929834 | 0.001360444 |
| ENSG00000185958 | family with sequence similarity 186 member A | 1.486974362 | 0.030558281 |
| ENSG00000126749 | EMG1, N1-specific pseudouridine methyltransferase | 1.483006988 | 0.000146091 |
| ENSG00000100429 | histone deacetylase 10 | 1.480131728 | 0.004960602 |
| ENSG00000114378 | hyaluronoglucosaminidase 1 | 1.479453753 | 0.005759964 |
| ENSG00000243279 | PRA1 domain family member 2 | 1.476926922 | 8.94E-07 |
| ENSG00000137101 | CD72 molecule | 1.476601081 | 0.014419901 |
| ENSG00000102109 | proprotein convertase subtilisin/kexin type 1 inhibitor | 1.47555603 | 1.70E-06 |
| ENSG00000065618 | collagen type XVII alpha 1 chain | 1.472494046 | 0.00037331 |
| ENSG00000258881 |  | 1.471998201 | 9.17E-05 |
| ENSG00000087076 | hydroxysteroid 17-beta dehydrogenase 14 | 1.467881211 | 0.007070955 |
| ENSG00000183828 | nudix hydrolase 14 | 1.465904457 | 8.68E-06 |
| ENSG00000105479 | coiled-coil domain containing 114 | 1.463559537 | 0.00498607 |
| ENSG00000115457 | insulin like growth factor binding protein 2 | 1.456526571 | 0.005919668 |
| ENSG00000141505 | asialoglycoprotein receptor 1 | 1.455285653 | 0.004031967 |
| ENSG00000214026 | mitochondrial ribosomal protein L23 | 1.453526209 | 0.001469725 |
| ENSG00000064886 | chitinase 3 like 2 | 1.452369225 | 0.001548382 |
| ENSG00000187608 | ISG15 ubiquitin-like modifier | 1.448300231 | 1.90E-06 |
| ENSG00000104953 | transducin like enhancer of split 6 | 1.446166045 | 0.003757801 |
| ENSG00000187642 | PPARGC1 and ESRR induced regulator, muscle 1 | 1.444147293 | 0.003375569 |
| ENSG00000162366 | PDZK1 interacting protein 1 | 1.442429365 | 0.000377009 |
| ENSG00000178773 | copine 7 | 1.442037288 | 0.002196637 |
| ENSG00000115461 | insulin like growth factor binding protein 5 | 1.441799666 | 0.016173251 |
| ENSG00000182853 | vitelline membrane outer layer 1 homolog | 1.441536045 | 0.00010565 |
| ENSG00000145824 | C-X-C motif chemokine ligand 14 | 1.440334298 | 0.004900437 |
| ENSG00000105404 | Rab acceptor 1 | 1.438752489 | 1.42E-06 |
| ENSG00000165757 | KIAA1462 | 1.430402581 | 0.003251592 |
| ENSG00000135929 | cytochrome P450 family 27 subfamily A member 1 | 1.429226935 | 0.016802502 |
| ENSG00000167779 | insulin like growth factor binding protein 6 | 1.428221959 | 0.001203301 |
| ENSG00000103187 | coactosin like F-actin binding protein 1 | 1.421561171 | 1.07E-05 |
| ENSG00000241563 | cortistatin | 1.418817392 | 0.028199814 |
| ENSG00000196923 | PDZ and LIM domain 7 | 1.417600081 | 5.57E-05 |
| ENSG00000114115 | retinol binding protein 1 | 1.414198821 | 0.033325204 |
| ENSG00000104140 | ras homolog family member V | 1.41396544 | 0.001303196 |
| ENSG00000215788 | TNF receptor superfamily member 25 | 1.413370904 | 0.002816375 |
| ENSG00000161888 | SPC24, NDC80 kinetochore complex component | 1.412978264 | 0.000541443 |
| ENSG00000106268 | nudix hydrolase 1 | 1.410807008 | 0.000187576 |
| ENSG00000173838 | membrane associated ring-CH-type finger 10 | 1.409078967 | 0.014798779 |
| ENSG00000126709 | interferon alpha inducible protein 6 | 1.408631935 | 1.70E-06 |
| ENSG00000166823 | mesoderm posterior bHLH transcription factor 1 | 1.4070953 | 0.000344238 |
| ENSG00000110375 | uroplakin 2 | 1.402983578 | 0.026845269 |
| ENSG00000112561 | transcription factor EB | 1.402089168 | 0.000608373 |
| ENSG00000257704 | InaF motif containing 1 | 1.384914726 | 0.000812088 |
| ENSG00000125995 | reactive oxygen species modulator 1 | 1.382450858 | 4.21E-05 |
| ENSG00000049089 | collagen type IX alpha 2 chain | 1.380597656 | 0.001088161 |
| ENSG00000146232 | NFKB inhibitor epsilon | 1.380360279 | 3.97E-06 |
| ENSG00000101115 | spalt like transcription factor 4 | 1.374121771 | 0.008922887 |
| ENSG00000009950 | MLX interacting protein like | 1.372717521 | 0.004843578 |
| ENSG00000214274 | angiogenin | 1.372705974 | 0.006787886 |
| ENSG00000088726 | transmembrane protein 40 | 1.371206217 | 1.64E-06 |
| ENSG00000111674 | enolase 2 | 1.369566823 | 0.002136011 |
| ENSG00000088002 | sulfotransferase family 2B member 1 | 1.369486094 | 0.020055849 |
| ENSG00000149591 | transgelin | 1.366795744 | 0.020208515 |
| ENSG00000172922 | ribonuclease H2 subunit C | 1.366074358 | 5.19E-07 |
| ENSG00000184492 | forkhead box D4-like 1 | 1.365579589 | 0.020655982 |
| ENSG00000100473 | cochlin | 1.364691028 | 0.004543463 |
| ENSG00000174886 | NADH:ubiquinone oxidoreductase subunit A11 | 1.361645824 | 0.000690141 |
| ENSG00000160221 | chromosome 21 open reading frame 33 | 1.359722327 | 0.001692492 |
| ENSG00000181404 | WAS protein family homolog 1 | 1.359220433 | 0.007642152 |
| ENSG00000186010 | NADH:ubiquinone oxidoreductase subunit A13 | 1.357962216 | 0.000747621 |
| ENSG00000129749 | cholinergic receptor nicotinic alpha 10 subunit | 1.357513722 | 0.006915215 |
| ENSG00000205544 | transmembrane protein 256 | 1.3565685 | 0.000928118 |
| ENSG00000137098 | sperm associated antigen 8 | 1.355264064 | 0.03476771 |
| ENSG00000180089 | transmembrane protein 86B | 1.355140528 | 0.011681185 |
| ENSG00000175197 | DNA damage inducible transcript 3 | 1.353667316 | 0.000178456 |
| ENSG00000105393 | BRISC and BRCA1 A complex member 1 | 1.352004718 | 0.000251409 |
| ENSG00000100908 | ER membrane protein complex subunit 9 | 1.35174903 | 3.37E-06 |
| ENSG00000117472 | tetraspanin 1 | 1.350087744 | 0.001249698 |
| ENSG00000061656 | sperm associated antigen 4 | 1.34609921 | 0.00154464 |
| ENSG00000139880 | cadherin 24 | 1.345757582 | 5.11E-06 |
| ENSG00000182154 | mitochondrial ribosomal protein L41 | 1.341436403 | 4.95E-05 |
| ENSG00000039523 | family with sequence similarity 65 member A | 1.337356055 | 0.000397095 |
| ENSG00000183048 | solute carrier family 25 member 10 | 1.336700232 | 0.00020147 |
| ENSG00000228300 | chromosome 19 open reading frame 24 | 1.336688787 | 1.51E-05 |
| ENSG00000198242 | ribosomal protein L23a | 1.334294968 | 0.000464625 |
| ENSG00000142102 | protein-glucosylgalactosylhydroxylysine glucosidase | 1.332632663 | 0.008999199 |
| ENSG00000180389 | ATP synthase, H+ transporting, mitochondrial F1 complex, epsilon subunit pseudogene 2 | 1.33183588 | 0.013065047 |
| ENSG00000171462 | delta like non-canonical Notch ligand 2 | 1.328534051 | 0.000757626 |
| ENSG00000269313 | MAGI family member, X-linked | 1.325401515 | 0.003108132 |
| ENSG00000168273 | small integral membrane protein 4 | 1.325196787 | 9.73E-05 |
| ENSG00000185519 | family with sequence similarity 131 member C | 1.324017403 | 0.008687145 |
| ENSG00000179085 | dolichyl-phosphate mannosyltransferase subunit 3 | 1.323538585 | 8.96E-05 |
| ENSG00000130810 | peter pan homolog (Drosophila) | 1.322878731 | 0.000230021 |
| ENSG00000183873 | sodium voltage-gated channel alpha subunit 5 | 1.322494617 | 0.001676442 |
| ENSG00000165496 | ribosomal protein L10 like | 1.322124734 | 0.01202319 |
| ENSG00000088882 | carboxypeptidase X, M14 family member 1 | 1.322094802 | 0.025183285 |
| ENSG00000184986 | transmembrane protein 121 | 1.319130705 | 1.11E-05 |
| ENSG00000204922 | ubiquinol-cytochrome c reductase complex assembly factor 3 | 1.318967951 | 0.000342561 |
| ENSG00000100276 | RAS like family 10 member A | 1.317186514 | 0.004333791 |
| ENSG00000049249 | TNF receptor superfamily member 9 | 1.314280901 | 0.002030842 |
| ENSG00000179862 | Cbp/p300 interacting transactivator with Glu/Asp rich carboxy-terminal domain 4 | 1.310609082 | 0.004901836 |
| ENSG00000198832 | selenoprotein M | 1.308584175 | 6.38E-05 |
| ENSG00000101000 | protein C receptor | 1.307294227 | 9.77E-09 |
| ENSG00000109321 | amphiregulin | 1.307122868 | 0.000952735 |
| ENSG00000101187 | solute carrier organic anion transporter family member 4A1 | 1.303333384 | 0.003518731 |
| ENSG00000177700 | RNA polymerase II subunit L | 1.301832515 | 1.24E-05 |
| ENSG00000128228 | stromal cell derived factor 2 like 1 | 1.299742698 | 9.81E-07 |
| ENSG00000159713 | tubulin polymerization promoting protein family member 3 | 1.298446327 | 0.002421736 |
| ENSG00000167700 | major facilitator superfamily domain containing 3 | 1.298124232 | 2.13E-07 |
| ENSG00000106333 | procollagen C-endopeptidase enhancer | 1.298124102 | 0.000277685 |
| ENSG00000105696 | transmembrane protein 59 like | 1.298034989 | 0.026280635 |
| ENSG00000105668 | uroplakin 1A | 1.293490445 | 0.010001074 |
| ENSG00000107281 | neural proliferation, differentiation and control 1 | 1.293371456 | 4.39E-05 |
| ENSG00000249992 | transmembrane protein 158 (gene/pseudogene) | 1.292693443 | 0.005591493 |
| ENSG00000171060 | chromosome 8 open reading frame 74 | 1.291667008 | 0.016585813 |
| ENSG00000267281 |  | 1.291197653 | 0.024161307 |
| ENSG00000187840 | eukaryotic translation initiation factor 4E binding protein 1 | 1.290620186 | 8.44E-06 |
| ENSG00000152082 | mitotic spindle organizing protein 2B | 1.288535897 | 0.000236006 |
| ENSG00000172590 | mitochondrial ribosomal protein L52 | 1.287298303 | 3.57E-05 |
| ENSG00000179271 | GADD45G interacting protein 1 | 1.286635199 | 0.000238871 |
| ENSG00000141854 | MISP family member 3 | 1.285410635 | 0.007261473 |
| ENSG00000103024 | NME/NM23 nucleoside diphosphate kinase 3 | 1.284466764 | 0.000202957 |
| ENSG00000174276 | zinc finger HIT-type containing 2 | 1.282208356 | 6.29E-06 |
| ENSG00000101460 | microtubule associated protein 1 light chain 3 alpha | 1.280547273 | 3.61E-05 |
| ENSG00000246705 | H2A histone family member J | 1.279743362 | 2.17E-06 |
| ENSG00000196497 | importin 4 | 1.276652586 | 0.000286064 |
| ENSG00000105737 | glutamate ionotropic receptor kainate type subunit 5 | 1.275391921 | 0.015847366 |
| ENSG00000092621 | phosphoglycerate dehydrogenase | 1.275037951 | 0.001613794 |
| ENSG00000103253 | hydroxyacylglutathione hydrolase-like | 1.274465598 | 0.000179102 |
| ENSG00000244187 | transmembrane protein 141 | 1.272805971 | 2.17E-05 |
| ENSG00000100092 | SH3 domain binding protein 1 | 1.272423786 | 3.51E-06 |
| ENSG00000125148 | metallothionein 2A | 1.272373659 | 1.94E-05 |
| ENSG00000070404 | follistatin like 3 | 1.269507129 | 0.001743519 |
| ENSG00000224877 | NADH:ubiquinone oxidoreductase complex assembly factor 8 | 1.263431227 | 0.000386418 |
| ENSG00000178896 | exosome component 4 | 1.261651786 | 0.000144919 |
| ENSG00000172366 | MAPK regulated corepressor interacting protein 2 | 1.260658969 | 0.000129305 |
| ENSG00000104856 | RELB proto-oncogene, NF-kB subunit | 1.260319105 | 1.38E-08 |
| ENSG00000167985 | succinate dehydrogenase complex assembly factor 2 | 1.258489156 | 0.001955545 |
| ENSG00000262814 | mitochondrial ribosomal protein L12 | 1.258307182 | 0.000793195 |
| ENSG00000154978 | vesicular, overexpressed in cancer, prosurvival protein 1 | 1.258082948 | 0.000982984 |
| ENSG00000109255 | neuromedin U | 1.257679039 | 0.00015723 |
| ENSG00000204052 | leucine rich repeat containing 73 | 1.256344618 | 0.000151453 |
| ENSG00000005075 | RNA polymerase II subunit J | 1.256138809 | 0.000758971 |
| ENSG00000105639 | Janus kinase 3 | 1.252654923 | 4.61E-06 |
| ENSG00000164897 | transmembrane and ubiquitin like domain containing 1 | 1.25239351 | 2.14E-05 |
| ENSG00000162910 | mitochondrial ribosomal protein L55 | 1.252235557 | 1.58E-05 |
| ENSG00000117984 | cathepsin D | 1.251895505 | 0.005704328 |
| ENSG00000164967 | ribonuclease P/MRP subunit p25 like | 1.251765724 | 2.73E-07 |
| ENSG00000171222 | SCAN domain containing 1 | 1.251702125 | 0.000207314 |
| ENSG00000163132 | msh homeobox 1 | 1.251414503 | 0.000128373 |
| ENSG00000177595 | p53-induced death domain protein 1 | 1.251057949 | 0.000779677 |
| ENSG00000180767 | carbohydrate sulfotransferase 13 | 1.248298393 | 0.000259613 |
| ENSG00000186897 | complement C1q like 4 | 1.245108249 | 0.009673751 |
| ENSG00000185033 | semaphorin 4B | 1.244415417 | 5.87E-06 |
| ENSG00000071859 | family with sequence similarity 50 member A | 1.243620135 | 0.000137276 |
| ENSG00000145287 | placenta specific 8 | 1.243262009 | 0.00044835 |
| ENSG00000088826 | spermine oxidase | 1.239371658 | 0.002036794 |
| ENSG00000170190 | solute carrier family 16 member 5 | 1.239269583 | 2.62E-07 |
| ENSG00000188643 | S100 calcium binding protein A16 | 1.238813556 | 0.002718808 |
| ENSG00000104903 | LYL1, basic helix-loop-helix family member | 1.238109666 | 0.001592235 |
| ENSG00000006453 | BAI1 associated protein 2 like 1 | 1.237493887 | 1.20E-06 |
| ENSG00000090971 | N-acetyltransferase 14 (putative) | 1.235507158 | 1.45E-05 |
| ENSG00000223802 | ceramide synthase 1 | 1.232668488 | 0.002698239 |
| ENSG00000103363 | transcription elongation factor B subunit 2 | 1.232404231 | 0.000170768 |
| ENSG00000128342 | leukemia inhibitory factor | 1.232273023 | 0.012567508 |
| ENSG00000130489 | SCO2, cytochrome c oxidase assembly protein | 1.231625246 | 0.000131841 |
| ENSG00000276345 |  | 1.231084407 | 0.002152407 |
| ENSG00000197852 | family with sequence similarity 212 member B | 1.228647606 | 0.003324963 |
| ENSG00000072163 | LIM zinc finger domain containing 2 | 1.22833547 | 0.003271201 |
| ENSG00000235098 | ankyrin repeat domain 65 | 1.227564138 | 0.002762232 |
| ENSG00000165752 | serine/threonine kinase 32C | 1.226505285 | 0.00021055 |
| ENSG00000111664 | G protein subunit beta 3 | 1.22604835 | 0.027601756 |
| ENSG00000115641 | four and a half LIM domains 2 | 1.225291444 | 0.000395956 |
| ENSG00000167964 | RAB26, member RAS oncogene family | 1.221673215 | 0.0016715 |
| ENSG00000101210 | eukaryotic translation elongation factor 1 alpha 2 | 1.221514593 | 0.005582073 |
| ENSG00000124257 | neuralized E3 ubiquitin protein ligase 2 | 1.220791048 | 0.024633212 |
| ENSG00000205155 | presenilin enhancer gamma-secretase subunit | 1.220533441 | 0.00227762 |
| ENSG00000068078 | fibroblast growth factor receptor 3 | 1.218579915 | 0.002935028 |
| ENSG00000115268 | ribosomal protein S15 | 1.218355816 | 0.000127181 |
| ENSG00000129946 | SHC adaptor protein 2 | 1.216144713 | 0.019474155 |
| ENSG00000160256 | family with sequence similarity 207 member A | 1.216109634 | 4.17E-05 |
| ENSG00000130005 | guanidinoacetate N-methyltransferase | 1.214934998 | 0.000363205 |
| ENSG00000110811 | prolyl 3-hydroxylase 3 | 1.214035781 | 0.012110355 |
| ENSG00000196420 | S100 calcium binding protein A5 | 1.213180794 | 0.001298311 |
| ENSG00000169169 | carnitine palmitoyltransferase 1C | 1.212192889 | 0.019449789 |
| ENSG00000099377 | hydroxy-delta-5-steroid dehydrogenase, 3 beta- and steroid delta-isomerase 7 | 1.21075701 | 7.83E-06 |
| ENSG00000185347 | chromosome 14 open reading frame 80 | 1.210319481 | 1.51E-05 |
| ENSG00000155366 | ras homolog family member C | 1.210271132 | 0.000288827 |
| ENSG00000183248 | proline rich 36 | 1.20936808 | 0.000828043 |
| ENSG00000233927 | ribosomal protein S28 | 1.207954224 | 0.000593909 |
| ENSG00000148362 | chromosome 9 open reading frame 142 | 1.207742986 | 0.001368511 |
| ENSG00000149541 | beta-1,3-glucuronyltransferase 3 | 1.206914192 | 3.12E-05 |
| ENSG00000163584 | ribosomal protein L22 like 1 | 1.206363184 | 2.68E-07 |
| ENSG00000116691 | migration and invasion inhibitory protein | 1.204893418 | 0.00154239 |
| ENSG00000049656 | CLPTM1 like | 1.20432552 | 0.000275059 |
| ENSG00000175602 | coiled-coil domain containing 85B | 1.204205027 | 0.000359236 |
| ENSG00000189159 | hematological and neurological expressed 1 | 1.203634664 | 9.78E-06 |
| ENSG00000100918 | REC8 meiotic recombination protein | 1.202670412 | 0.010302423 |
| ENSG00000121900 | transmembrane protein 54 | 1.202319452 | 0.000153186 |
| ENSG00000089820 | Rho GTPase activating protein 4 | 1.201511274 | 0.006711357 |
| ENSG00000126432 | peroxiredoxin 5 | 1.200904586 | 5.47E-06 |
| ENSG00000247596 | twinfilin actin binding protein 2 | 1.200616321 | 3.77E-06 |
| ENSG00000160813 | protein phosphatase 1 regulatory subunit 35 | 1.200316686 | 0.000397814 |
| ENSG00000214756 | methyltransferase like 12 | 1.198527028 | 0.000445948 |
| ENSG00000163126 | ankyrin repeat domain 23 | 1.19821626 | 0.030145313 |
| ENSG00000166136 | NADH:ubiquinone oxidoreductase subunit B8 | 1.197275469 | 0.002903692 |
| ENSG00000108961 | RAN guanine nucleotide release factor | 1.19660233 | 0.004369317 |
| ENSG00000103145 | host cell factor C1 regulator 1 | 1.195758053 | 0.000509734 |
| ENSG00000183011 | N(alpha)-acetyltransferase 38, NatC auxiliary subunit | 1.195378556 | 0.001061866 |
| ENSG00000099385 | BCL tumor suppressor 7C | 1.192402529 | 0.000332638 |
| ENSG00000103512 | NODAL modulator 1 | 1.192008145 | 0.009802385 |
| ENSG00000133131 | MORC family CW-type zinc finger 4 | 1.191878102 | 0.01280638 |
| ENSG00000169738 | dicarbonyl and L-xylulose reductase | 1.190848507 | 0.002002988 |
| ENSG00000110888 | caprin family member 2 | 1.190297268 | 0.009762019 |
| ENSG00000225663 | MAPK regulated corepressor interacting protein 1 | 1.189686507 | 0.001292395 |
| ENSG00000140365 | COMM domain containing 4 | 1.185209872 | 0.000305473 |
| ENSG00000188186 | late endosomal/lysosomal adaptor, MAPK and MTOR activator 4 | 1.183483749 | 0.001379782 |
| ENSG00000177380 | PTPRF interacting protein alpha 3 | 1.182475405 | 0.000192232 |
| ENSG00000185043 | calcium and integrin binding 1 | 1.182140608 | 3.74E-05 |
| ENSG00000170955 | protein kinase C delta binding protein | 1.179749662 | 0.000723966 |
| ENSG00000168061 | SAC3 domain containing 1 | 1.179616847 | 0.000412095 |
| ENSG00000239672 | NME/NM23 nucleoside diphosphate kinase 1 | 1.179317374 | 0.000378178 |
| ENSG00000107872 | F-box and leucine rich repeat protein 15 | 1.177866857 | 0.000231906 |
| ENSG00000179588 | zinc finger protein, FOG family member 1 | 1.177192363 | 0.000259819 |
| ENSG00000175756 | aurora kinase A interacting protein 1 | 1.173969654 | 0.001245042 |
| ENSG00000205362 | metallothionein 1A | 1.172041605 | 0.001264275 |
| ENSG00000185201 | interferon induced transmembrane protein 2 | 1.171859708 | 5.88E-05 |
| ENSG00000102890 | engulfment and cell motility 3 | 1.17178034 | 0.011339215 |
| ENSG00000051523 | cytochrome b-245 alpha chain | 1.171629341 | 5.17E-05 |
| ENSG00000173992 | copper chaperone for superoxide dismutase | 1.171416826 | 0.000217428 |
| ENSG00000156873 | phosphorylase kinase catalytic subunit gamma 2 | 1.171401679 | 0.000597812 |
| ENSG00000161513 | ferredoxin reductase | 1.170476045 | 0.000111875 |
| ENSG00000157778 | proteasome assembly chaperone 3 | 1.169477762 | 0.000393488 |
| ENSG00000167799 | nudix hydrolase 8 | 1.168877966 | 0.000730326 |
| ENSG00000177600 | ribosomal protein lateral stalk subunit P2 | 1.167875502 | 0.000952402 |
| ENSG00000162572 | sodium channel epithelial 1 delta subunit | 1.167409323 | 0.035427864 |
| ENSG00000171163 | zinc finger protein 692 | 1.167229695 | 0.028507626 |
| ENSG00000105711 | sodium voltage-gated channel beta subunit 1 | 1.167196142 | 0.018214805 |
| ENSG00000261236 | block of proliferation 1 | 1.166764557 | 1.92E-05 |
| ENSG00000086504 | mitochondrial ribosomal protein L28 | 1.165755016 | 0.000180183 |
| ENSG00000142046 | transmembrane protein 91 | 1.165454806 | 0.02596916 |
| ENSG00000099625 | CACN beta subunit associated regulatory protein | 1.162758979 | 0.000323484 |
| ENSG00000204237 | oxidoreductase like domain containing 1 | 1.16269139 | 0.000179429 |
| ENSG00000130731 | methyltransferase like 26 | 1.162494997 | 0.001071451 |
| ENSG00000137133 | histidine triad nucleotide binding protein 2 | 1.161854681 | 0.001049445 |
| ENSG00000177556 | antioxidant 1 copper chaperone | 1.161784968 | 0.001748005 |
| ENSG00000167543 | tumor protein p53 inducible protein 13 | 1.161620457 | 2.52E-05 |
| ENSG00000186577 | chromosome 6 open reading frame 1 | 1.161487556 | 7.88E-05 |
| ENSG00000167646 | dynein axonemal assembly factor 3 | 1.161365963 | 0.019918371 |
| ENSG00000130332 | LSM7 homolog, U6 small nuclear RNA and mRNA degradation associated | 1.160189185 | 0.000687461 |
| ENSG00000233276 | glutathione peroxidase 1 | 1.159696306 | 0.000108321 |
| ENSG00000130748 | transmembrane protein 160 | 1.159324156 | 0.001559409 |
| ENSG00000184281 | tumor suppressing subtransferable candidate 4 | 1.157943349 | 1.62E-05 |
| ENSG00000105499 | phospholipase A2 group IVC | 1.157913726 | 0.001614318 |
| ENSG00000105583 | WD repeat domain 83 opposite strand | 1.156567666 | 0.001903995 |
| ENSG00000126759 | complement factor properdin | 1.15482492 | 0.031061851 |
| ENSG00000106733 | nicotinamide riboside kinase 1 | 1.154664276 | 0.019979592 |
| ENSG00000168255 | RNA polymerase II subunit J3 | 1.154293425 | 0.022723156 |
| ENSG00000229833 | PET100 homolog | 1.153435382 | 0.003110653 |
| ENSG00000178980 | selenoprotein W | 1.153112141 | 3.57E-06 |
| ENSG00000140264 | small EDRK-rich factor 2 | 1.152464417 | 0.000220071 |
| ENSG00000135441 | biogenesis of lysosomal organelles complex 1 subunit 1 | 1.1522316 | 0.001066126 |
| ENSG00000213977 | Tax1 binding protein 3 | 1.151059722 | 0.008872026 |
| ENSG00000164611 | pituitary tumor-transforming 1 | 1.150534998 | 9.66E-05 |
| ENSG00000173486 | FK506 binding protein 2 | 1.150532282 | 0.000310454 |
| ENSG00000167641 | protein phosphatase 1 regulatory inhibitor subunit 14A | 1.150333047 | 0.006646781 |
| ENSG00000100836 | poly(A) binding protein nuclear 1 | 1.149941928 | 0.000566964 |
| ENSG00000119705 | SRA stem-loop interacting RNA binding protein | 1.149782828 | 0.000981215 |
| ENSG00000105518 | transmembrane protein 205 | 1.149213928 | 0.000757939 |
| ENSG00000214530 | StAR related lipid transfer domain containing 10 | 1.149144598 | 7.04E-05 |
| ENSG00000188488 | serpin family A member 5 8723] | 1.148585792 | 0.006767526 |
| ENSG00000106992 | adenylate kinase 1 | 1.148139096 | 0.000205417 |
| ENSG00000162585 | Fanconi anemia core complex associated protein 20 | 1.146361674 | 0.000854769 |
| ENSG00000099330 | occludin/ELL domain containing 1 | 1.145817022 | 0.000556706 |
| ENSG00000073169 | selenoprotein O | 1.145491986 | 0.000712002 |
| ENSG00000102003 | synaptophysin | 1.14533761 | 0.000284941 |
| ENSG00000106211 | heat shock protein family B (small) member 1 | 1.144892153 | 0.000646302 |
| ENSG00000104368 | plasminogen activator, tissue type | 1.143556394 | 0.021837428 |
| ENSG00000127540 | ubiquinol-cytochrome c reductase, complex III subunit XI | 1.143029259 | 0.002070956 |
| ENSG00000198816 | zinc finger protein 358 | 1.142869197 | 0.003679132 |
| ENSG00000166337 | TATA-box binding protein associated factor 10 | 1.142273852 | 1.10E-08 |
| ENSG00000147123 | NADH:ubiquinone oxidoreductase subunit B11 | 1.141852483 | 0.001508777 |
| ENSG00000173465 | Sjogren syndrome/scleroderma autoantigen 1 | 1.140774077 | 1.97E-05 |
| ENSG00000099624 | ATP synthase, H+ transporting, mitochondrial F1 complex, delta subunit | 1.140179828 | 0.001400832 |
| ENSG00000175854 | SWI5 homologous recombination repair protein | 1.138740373 | 0.000516293 |
| ENSG00000141933 | tubulin polyglutamylase complex subunit 1 | 1.13824726 | 0.000631252 |
| ENSG00000185215 | TNF alpha induced protein 2 | 1.1377529 | 0.000620531 |
| ENSG00000064547 | lysophosphatidic acid receptor 2 | 1.137691464 | 0.031795169 |
| ENSG00000147155 | emopamil binding protein (sterol isomerase) | 1.137521239 | 0.000474776 |
| ENSG00000188130 | mitogen-activated protein kinase 12 | 1.136828433 | 6.05E-05 |
| ENSG00000213015 | zinc finger protein 580 | 1.136008407 | 0.000549326 |
| ENSG00000136908 | dolichyl-phosphate mannosyltransferase subunit 2, regulatory | 1.135364807 | 0.000473549 |
| ENSG00000154146 | neurogranin | 1.134933963 | 0.001794756 |
| ENSG00000135740 | solute carrier family 9 member A5 | 1.133495867 | 0.005716293 |
| ENSG00000162783 | immediate early response 5 | 1.131830043 | 0.00104758 |
| ENSG00000107223 | endothelial differentiation related factor 1 | 1.131788314 | 0.000576297 |
| ENSG00000242372 | eukaryotic translation initiation factor 6 | 1.131276945 | 4.46E-05 |
| ENSG00000178026 | leucine rich repeat containing 75B | 1.130762406 | 0.001426435 |
| ENSG00000160606 | TLC domain containing 1 | 1.129983705 | 1.80E-05 |
| ENSG00000196976 | L antigen family member 3 | 1.129498793 | 0.001156441 |
| ENSG00000169020 | ATP synthase, H+ transporting, mitochondrial Fo complex subunit E | 1.128018303 | 0.003317023 |
| ENSG00000067798 | neuron navigator 3 | 1.127217606 | 0.019499912 |
| ENSG00000189171 | S100 calcium binding protein A13 | 1.126814044 | 0.001502601 |
| ENSG00000173991 | titin-cap | 1.126619466 | 0.008020032 |
| ENSG00000167775 | CD320 molecule | 1.125655398 | 0.000387054 |
| ENSG00000147804 | solute carrier family 39 member 4 | 1.125337191 | 1.10E-05 |
| ENSG00000055118 | potassium voltage-gated channel subfamily H member 2 | 1.124342313 | 0.019174025 |
| ENSG00000111057 | keratin 18 | 1.124319538 | 0.001591746 |
| ENSG00000188092 | G protein-coupled receptor 89B | 1.123380204 | 0.002124902 |
| ENSG00000162066 | amidohydrolase domain containing 2 | 1.122408518 | 2.35E-05 |
| ENSG00000158769 | F11 receptor | 1.121525987 | 0.00734471 |
| ENSG00000188015 | S100 calcium binding protein A3 | 1.120973179 | 0.000417636 |
| ENSG00000163794 | urocortin | 1.120536597 | 0.034282372 |
| ENSG00000213741 | ribosomal protein S29 | 1.119979644 | 0.000161845 |
| ENSG00000163701 | interleukin 17 receptor E | 1.119433013 | 9.45E-05 |
| ENSG00000125144 | metallothionein 1G | 1.119313862 | 0.000227146 |
| ENSG00000131495 | NADH:ubiquinone oxidoreductase subunit A2 | 1.119023714 | 0.002109826 |
| ENSG00000173137 | aarF domain containing kinase 5 | 1.118619665 | 0.000969626 |
| ENSG00000103254 | family with sequence similarity 173 member A | 1.118247574 | 0.001334432 |
| ENSG00000125898 | family with sequence similarity 110 member A | 1.116200276 | 0.001358278 |
| ENSG00000110717 | NADH:ubiquinone oxidoreductase core subunit S8 | 1.116046009 | 0.000401393 |
| ENSG00000107485 | GATA binding protein 3 | 1.115310804 | 0.017550173 |
| ENSG00000130653 | patatin like phospholipase domain containing 7 | 1.114910089 | 0.011221524 |
| ENSG00000118292 | chromosome 1 open reading frame 54 | 1.113579986 | 0.02872213 |
| ENSG00000142544 | cytosolic thiouridylase subunit 1 | 1.112476536 | 0.000407409 |
| ENSG00000172428 | COP9 signalosome subunit 9 | 1.111974771 | 0.00129843 |
| ENSG00000244165 | purinergic receptor P2Y11 | 1.111375172 | 0.007696442 |
| ENSG00000178445 | glycine decarboxylase | 1.110668154 | 0.007092757 |
| ENSG00000169733 | RFNG O-fucosylpeptide 3-beta-N-acetylglucosaminyltransferase | 1.11029398 | 0.000146919 |
| ENSG00000162062 | chromosome 16 open reading frame | 1.108845662 | 0.000550945 |
| ENSG00000221821 | chromosome 6 open reading frame 226 | 1.108412057 | 0.001772868 |
| ENSG00000188878 | Fas binding factor 1 | 1.108314148 | 0.008831369 |
| ENSG00000166035 | lipase C, hepatic type | 1.108164948 | 0.013237817 |
| ENSG00000166165 | creatine kinase B | 1.107728677 | 0.004446726 |
| ENSG00000158106 | rhophilin Rho GTPase binding protein 1 | 1.107632127 | 0.031683983 |
| ENSG00000168528 | serine incorporator 2 | 1.106572531 | 6.44E-05 |
| ENSG00000241468 | ATP synthase, H+ transporting, mitochondrial Fo complex subunit F2 | 1.105200299 | 0.001298341 |
| ENSG00000136840 | ST6 N-acetylgalactosaminide alpha-2,6-sialyltransferase 4 | 1.103922273 | 8.59E-05 |
| ENSG00000164896 | Fas activated serine/threonine kinase | 1.102418538 | 0.00192968 |
| ENSG00000159199 | ATP synthase, H+ transporting, mitochondrial Fo complex subunit C1 (subunit 9) | 1.10239464 | 0.000949616 |
| ENSG00000142546 | nitric oxide synthase interacting protein | 1.102372957 | 0.001182584 |
| ENSG00000243725 | tetratricopeptide repeat domain 4 | 1.101575571 | 0.014678834 |
| ENSG00000242114 | mitochondrial fission process 1 | 1.100958114 | 0.002599835 |
| ENSG00000161509 | glutamate ionotropic receptor NMDA type subunit 2C | 1.100270381 | 0.002161641 |
| ENSG00000161265 | U2 small nuclear RNA auxiliary factor 1 like 4 | 1.099791418 | 0.030085171 |
| ENSG00000198003 | coiled-coil domain containing 151 | 1.098294345 | 0.012771525 |
| ENSG00000011332 | double PHD fingers 1 | 1.097666287 | 8.95E-05 |
| ENSG00000142227 | epithelial membrane protein 3 | 1.096727633 | 0.000526101 |
| ENSG00000006837 | cyclin dependent kinase like 3 | 1.095994572 | 0.030640894 |
| ENSG00000065054 | SLC9A3 regulator 2 | 1.09591452 | 5.00E-06 |
| ENSG00000135314 | KH homology domain containing 1 | 1.095129463 | 9.53E-05 |
| ENSG00000130775 | thymocyte selection associated family member 2 | 1.094600908 | 0.012647838 |
| ENSG00000149806 | FAU, ubiquitin like and ribosomal protein S30 fusion | 1.094322361 | 4.91E-05 |
| ENSG00000133315 | MACRO domain containing 1 | 1.094146234 | 0.000102612 |
| ENSG00000184967 | nucleolar complex associated 4 homolog | 1.093828643 | 0.00029545 |
| ENSG00000127824 | tubulin alpha 4a | 1.092480609 | 0.005736709 |
| ENSG00000164898 | formation of mitochondrial complex V assembly factor 1 homolog | 1.092460724 | 0.002987462 |
| ENSG00000205643 | cysteine rich DPF motif domain containing 1 | 1.090479706 | 0.001051701 |
| ENSG00000145494 | NADH:ubiquinone oxidoreductase subunit S6 | 1.089396103 | 0.004364735 |
| ENSG00000169242 | ephrin A1 | 1.089003915 | 7.63E-05 |
| ENSG00000241553 | actin related protein 2/3 complex subunit 4 | 1.088888383 | 0.000574909 |
| ENSG00000104979 | chromosome 19 open reading frame 53 | 1.088609302 | 0.001172859 |
| ENSG00000185386 | mitogen-activated protein kinase 11 | 1.088512666 | 0.000709258 |
| ENSG00000168701 | transmembrane protein 208 | 1.087072221 | 0.000719336 |
| ENSG00000184076 | ubiquinol-cytochrome c reductase, complex III subunit X | 1.087035124 | 0.001623883 |
| ENSG00000069482 | galanin and GMAP prepropeptide | 1.086134533 | 2.01E-08 |
| ENSG00000182749 | progestin and adipoQ receptor family member 7 | 1.085170757 | 6.65E-05 |
| ENSG00000197982 | chromosome 1 open reading frame 122 | 1.085084687 | 8.52E-05 |
| ENSG00000137309 | high mobility group AT-hook 1 | 1.084625748 | 0.000594775 |
| ENSG00000164008 | chromosome 1 open reading frame 50 | 1.083882416 | 0.023110428 |
| ENSG00000139410 | serine dehydratase like | 1.083308194 | 0.0040991 |
| ENSG00000172889 | EGF like domain multiple 7 | 1.082666375 | 0.001296759 |
| ENSG00000137218 | fibroblast growth factor receptor substrate 3 | 1.082318404 | 0.003768501 |
| ENSG00000176340 | cytochrome c oxidase subunit 8A | 1.081278648 | 0.000205755 |
| ENSG00000108107 | ribosomal protein L28 | 1.08080862 | 0.002444746 |
| ENSG00000131435 | PDZ and LIM domain 4 | 1.080191096 | 2.83E-07 |
| ENSG00000169189 | NSE1 homolog, SMC5-SMC6 complex component | 1.078767696 | 0.001347603 |
| ENSG00000167550 | Ras homolog enriched in brain like 1 | 1.077348037 | 0.006252537 |
| ENSG00000105255 | fibronectin type III and SPRY domain containing 1 | 1.076885012 | 0.00117062 |
| ENSG00000101246 | ADP ribosylation factor related protein 1 | 1.076084512 | 0.000291909 |
| ENSG00000177697 | CD151 molecule (Raph blood group) | 1.075912659 | 2.34E-06 |
| ENSG00000223573 | tissue differentiation-inducing non-protein coding RNA | 1.07546363 | 0.002370395 |
| ENSG00000173156 | ras homolog family member D | 1.075406537 | 2.25E-06 |
| ENSG00000119333 | WD repeat domain 34 | 1.073080328 | 0.001164842 |
| ENSG00000174791 | Ras and Rab interactor 1 | 1.072945194 | 0.002794988 |
| ENSG00000213465 | ADP ribosylation factor like GTPase 2 | 1.072432195 | 0.000741387 |
| ENSG00000160972 | protein phosphatase 1 regulatory subunit 16A | 1.07120268 | 0.000166171 |
| ENSG00000163739 | C-X-C motif chemokine ligand 1 | 1.070715221 | 0.005097663 |
| ENSG00000218891 | zinc finger protein 579 | 1.070609488 | 0.000196402 |
| ENSG00000023445 | baculoviral IAP repeat containing 3 | 1.069991322 | 0.001157547 |
| ENSG00000128965 | ChaC glutathione specific gamma-glutamylcyclotransferase 1 | 1.069673686 | 0.012916471 |
| ENSG00000248487 | abhydrolase domain containing 14A | 1.069166101 | 0.008418877 |
| ENSG00000126453 | BCL2 like 12 | 1.068495284 | 0.000119068 |
| ENSG00000101335 | myosin light chain 9 | 1.068296975 | 0.000773837 |
| ENSG00000090266 | NADH:ubiquinone oxidoreductase subunit B2 | 1.068221222 | 0.003108194 |
| ENSG00000104886 | pleckstrin homology domain containing J1 | 1.068145971 | 5.88E-06 |
| ENSG00000124104 | sorting nexin family member 21 | 1.066972041 | 0.00206619 |
| ENSG00000198276 | uridine-cytidine kinase 1 like 1 | 1.066292271 | 0.002212749 |
| ENSG00000213626 | limb bud and heart development | 1.064822278 | 0.031250617 |
| ENSG00000111678 | chromosome 12 open reading frame 57 | 1.064727137 | 0.001428453 |
| ENSG00000125089 | SH3 domain and tetratricopeptide repeats 1 | 1.064427123 | 6.45E-05 |
| ENSG00000127952 | serine/threonine/tyrosine interacting like 1 | 1.061837398 | 0.000619226 |
| ENSG00000102030 | N(alpha)-acetyltransferase 10, NatA catalytic subunit | 1.061128829 | 0.002021125 |
| ENSG00000197728 | ribosomal protein S26 | 1.060266238 | 0.003071985 |
| ENSG00000107338 | SH2 domain containing adaptor protein B | 1.057843464 | 0.004242016 |
| ENSG00000102125 | tafazzin | 1.057359693 | 0.003351227 |
| ENSG00000187193 | metallothionein 1X | 1.05706429 | 0.001711617 |
| ENSG00000164976 | KIAA1161 | 1.056815574 | 0.012888295 |
| ENSG00000131459 | glutamine-fructose-6-phosphate transaminase 2 | 1.05577778 | 0.00820905 |
| ENSG00000125458 | 5', 3'-nucleotidase, cytosolic | 1.055193674 | 0.001330632 |
| ENSG00000250254 | pituitary tumor-transforming 2 | 1.054282205 | 0.019396605 |
| ENSG00000173531 | macrophage stimulating 1 | 1.054210232 | 0.0199199 |
| ENSG00000120885 | clusterin | 1.053650536 | 0.003913492 |
| ENSG00000126267 | cytochrome c oxidase subunit 6B1 | 1.053229901 | 0.002353217 |
| ENSG00000127415 | iduronidase, alpha-L- | 1.052694769 | 0.017629128 |
| ENSG00000105655 | inositol-3-phosphate synthase 1 | 1.052267336 | 1.82E-05 |
| ENSG00000106077 | abhydrolase domain containing 11 | 1.051581519 | 6.63E-05 |
| ENSG00000167797 | cyclin dependent kinase 2 associated protein 2 | 1.051044023 | 0.000316367 |
| ENSG00000269858 | egl-9 family hypoxia inducible factor 2 | 1.05055037 | 0.00718299 |
| ENSG00000132535 | discs large MAGUK scaffold protein 4 | 1.050505599 | 0.009508901 |
| ENSG00000250571 | GLI family zinc finger 4 | 1.04999863 | 0.006912136 |
| ENSG00000185298 | coiled-coil domain containing 137 | 1.049065597 | 0.000130498 |
| ENSG00000108786 | hydroxysteroid 17-beta dehydrogenase 1 | 1.04771889 | 7.21E-06 |
| ENSG00000140691 | armadillo repeat containing 5 | 1.047374522 | 5.66E-05 |
| ENSG00000105379 | electron transfer flavoprotein beta subunit | 1.04720591 | 0.00512927 |
| ENSG00000154134 | roundabout guidance receptor 3 | 1.046884014 | 0.007481168 |
| ENSG00000117691 | neudesin neurotrophic factor | 1.046880658 | 0.00097901 |
| ENSG00000164713 | brain protein I3 | 1.046197235 | 9.15E-05 |
| ENSG00000185860 | coiled-coil domain containing 190 | 1.045846946 | 0.002470887 |
| ENSG00000049759 | neural precursor cell expressed, developmentally down-regulated 4-like, E3 ubiquitin protein ligase | 1.045244816 | 0.002965819 |
| ENSG00000025434 | nuclear receptor subfamily 1 group H member 3 | 1.044757024 | 0.00154758 |
| ENSG00000177854 | transmembrane protein 187 | 1.044070031 | 0.002882377 |
| ENSG00000135083 | cyclin J like | 1.043746636 | 0.001777361 |
| ENSG00000167771 | REST corepressor 2 | 1.043638956 | 0.010215438 |
| ENSG00000126768 | translocase of inner mitochondrial membrane 17 homolog B (yeast) | 1.043500437 | 0.000814788 |
| ENSG00000149809 | transmembrane 7 superfamily member 2 | 1.042761315 | 0.000760766 |
| ENSG00000213347 | MAX dimerization protein 3 | 1.040759397 | 0.004097433 |
| ENSG00000100162 | centromere protein M | 1.040232229 | 0.001700063 |
| ENSG00000197019 | SERTA domain containing 1 | 1.039151293 | 4.52E-05 |
| ENSG00000161677 | Josephin domain containing 2 | 1.038789812 | 0.001346185 |
| ENSG00000167930 | family with sequence similarity 234 member A | 1.036843866 | 0.00060784 |
| ENSG00000127252 | HRAS like suppressor | 1.036353128 | 0.000338819 |
| ENSG00000149798 | CDC42 effector protein 2 | 1.035717328 | 0.004238207 |
| ENSG00000075618 | fascin actin-bundling protein 1 | 1.035485053 | 0.023156921 |
| ENSG00000167468 | glutathione peroxidase 4 | 1.035144002 | 0.00051178 |
| ENSG00000109736 | major facilitator superfamily domain containing 10 | 1.034702867 | 0.000103194 |
| ENSG00000185262 | UBA like domain containing 2 | 1.034662736 | 0.011488235 |
| ENSG00000102901 | centromere protein T | 1.032638381 | 0.023437667 |
| ENSG00000189306 | ribosomal RNA processing 7 homolog A | 1.031663655 | 3.69E-05 |
| ENSG00000182272 | beta-1,4-N-acetyl-galactosaminyltransferase 4 | 1.030736598 | 0.000543548 |
| ENSG00000124172 | ATP synthase, H+ transporting, mitochondrial F1 complex, epsilon subunit | 1.029806232 | 0.003646594 |
| ENSG00000162755 | kelch domain containing 9 | 1.029487333 | 5.31E-05 |
| ENSG00000103154 | N-terminal EF-hand calcium binding protein 2 | 1.029080307 | 0.004179355 |
| ENSG00000123144 | chromosome 19 open reading frame 43 | 1.028963592 | 0.001237573 |
| ENSG00000077312 | small nuclear ribonucleoprotein polypeptide A | 1.02852957 | 0.003992912 |
| ENSG00000156509 | F-box protein 43 | 1.028470847 | 0.016648786 |
| ENSG00000167302 | TEPSIN, adaptor related protein complex 4 accessory protein | 1.027444138 | 0.008436595 |
| ENSG00000107833 | nucleophosmin/nucleoplasmin 3 | 1.026289772 | 2.75E-06 |
| ENSG00000173272 | mitotic spindle organizing protein 2A | 1.025868476 | 8.10E-05 |
| ENSG00000051128 | homer scaffolding protein 3 | 1.025772413 | 0.000203842 |
| ENSG00000167701 | glutamic--pyruvic transaminase | 1.025139468 | 0.0206368 |
| ENSG00000160072 | ATPase family, AAA domain containing 3B | 1.024324888 | 0.007768845 |
| ENSG00000092841 | myosin light chain 6 | 1.023947915 | 0.000814693 |
| ENSG00000235961 | paraneoplastic Ma antigen family member 6A | 1.023679775 | 0.006709086 |
| ENSG00000260001 | transforming growth factor beta receptor 3 like | 1.023043488 | 0.010245711 |
| ENSG00000173457 | protein phosphatase 1 regulatory inhibitor subunit 14B | 1.023027757 | 9.60E-05 |
| ENSG00000198546 | zinc finger protein 511 | 1.022796442 | 0.005840436 |
| ENSG00000174851 | Yip1 interacting factor homolog A, membrane trafficking protein | 1.022326658 | 1.84E-05 |
| ENSG00000184207 | phosphoglycolate phosphatase | 1.022045277 | 0.000116921 |
| ENSG00000159640 | angiotensin I converting enzyme | 1.021451674 | 0.030433184 |
| ENSG00000184897 | H1 histone family member X | 1.020893043 | 0.002006009 |
| ENSG00000105401 | cell division cycle 37 | 1.0203378 | 3.47E-05 |
| ENSG00000176476 | SAGA complex associated factor 29 | 1.020276404 | 0.002785232 |
| ENSG00000169692 | 1-acylglycerol-3-phosphate O-acyltransferase 2 | 1.019679342 | 0.000413761 |
| ENSG00000167525 | protein interacting with cyclin A1 | 1.019310853 | 0.027980194 |
| ENSG00000105258 | RNA polymerase II subunit I | 1.019049952 | 0.012357398 |
| ENSG00000168894 | ring finger protein 181 | 1.017360605 | 0.000463741 |
| ENSG00000175701 | long intergenic non-protein coding RNA 116 | 1.017183973 | 0.003637565 |
| ENSG00000130304 | solute carrier family 27 member 1 | 1.016732096 | 1.95E-05 |
| ENSG00000171858 | ribosomal protein S21 | 1.016207907 | 0.000494001 |
| ENSG00000221983 | ubiquitin A-52 residue ribosomal protein fusion product 1 | 1.01537396 | 0.000711456 |
| ENSG00000102981 | par-6 family cell polarity regulator alpha | 1.015217946 | 0.001086823 |
| ENSG00000114268 | 6-phosphofructo-2-kinase/fructose-2,6-biphosphatase 4 | 1.014760341 | 0.023822284 |
| ENSG00000105372 | ribosomal protein S19 | 1.014668472 | 0.00084786 |
| ENSG00000164877 | MICAL like 2 | 1.0141917 | 0.003860902 |
| ENSG00000167604 | NFKB inhibitor delta | 1.013498713 | 0.003355629 |
| ENSG00000006015 | chromosome 19 open reading frame 60 | 1.013457224 | 0.002655901 |
| ENSG00000142252 | gem nuclear organelle associated protein 7 | 1.013369142 | 0.000967784 |
| ENSG00000256053 | apoptogenic 1, mitochondrial | 1.013260327 | 0.001464107 |
| ENSG00000166595 | family with sequence similarity 96 member B | 1.011780696 | 0.000482498 |
| ENSG00000149016 | terminal uridylyl transferase 1, U6 snRNA-specific | 1.011284879 | 0.000699004 |
| ENSG00000138621 | phosphopantothenoylcysteine decarboxylase | 1.010044219 | 0.006184558 |
| ENSG00000132016 | chromosome 19 open reading frame 57 | 1.009817006 | 0.018041973 |
| ENSG00000065057 | nth like DNA glycosylase 1 | 1.009285519 | 0.001514859 |
| ENSG00000077463 | sirtuin 6 | 1.008834073 | 8.15E-05 |
| ENSG00000183648 | NADH:ubiquinone oxidoreductase subunit B1 | 1.008542767 | 0.006545073 |
| ENSG00000184990 | SIVA1 apoptosis inducing factor | 1.008041493 | 0.000273364 |
| ENSG00000186854 | TraB domain containing 2A | 1.007713003 | 0.004137884 |
| ENSG00000188997 | potassium channel tetramerization domain containing 21 | 1.007291623 | 0.000796347 |
| ENSG00000099992 | TBC1 domain family member 10A | 1.006786887 | 0.000654304 |
| ENSG00000179344 | major histocompatibility complex, class II, DQ beta 1 | 1.006429097 | 0.026825745 |
| ENSG00000126458 | related RAS viral (r-ras) oncogene homolog | 1.006300849 | 0.000327921 |
| ENSG00000140682 | transforming growth factor beta 1 induced transcript 1 | 1.005654386 | 2.55E-05 |
| ENSG00000237190 | CDKN2A interacting protein N-terminal like | 1.004735052 | 0.0015633 |
| ENSG00000124145 | syndecan 4 | 1.004692561 | 0.000211378 |
| ENSG00000122574 | WAS/WASL interacting protein family member 3 | 1.004614109 | 0.032346756 |
| ENSG00000168389 | major facilitator superfamily domain containing 2A | 1.003617968 | 0.001961296 |
| ENSG00000137474 | myosin VIIA | 1.00353941 | 0.00532019 |
| ENSG00000099795 | NADH:ubiquinone oxidoreductase subunit B7 | 1.002672272 | 0.002411061 |
| ENSG00000130066 | spermidine/spermine N1-acetyltransferase 1 | 1.002603997 | 0.000568424 |
| ENSG00000205138 | succinate dehydrogenase complex assembly factor 1 | 1.002310228 | 0.001159437 |
| ENSG00000109475 | ribosomal protein L34 | 1.002034986 | 0.002802207 |
| ENSG00000137288 | ubiquinol-cytochrome c reductase complex assembly factor 2 | 1.0014774 | 0.003137621 |
| ENSG00000103066 | phospholipase A2 group XV | 1.000740848 | 1.38E-05 |
| ENSG00000226174 | testis expressed 22 | 1.000154867 | 0.007982517 |
| ENSG00000166441 | ribosomal protein L27a | 1.000001893 | 0.001399142 |
| ENSG00000254685 | fucose-1-phosphate guanylyltransferase | -1.000976952 | 0.004317229 |
| ENSG00000084733 | RAB10, member RAS oncogene family | -1.001094259 | 0.000795258 |
| ENSG00000087338 | germ cell-less, spermatogenesis associated 1 | -1.001157542 | 0.001163223 |
| ENSG00000137822 | tubulin gamma complex associated protein 4 | -1.001162206 | 0.002621291 |
| ENSG00000164933 | solute carrier family 25 member 32 | -1.001876297 | 0.004587708 |
| ENSG00000120327 | protocadherin beta 14 | -1.002278024 | 0.025016207 |
| ENSG00000116641 | dedicator of cytokinesis 7 | -1.002471715 | 7.70E-07 |
| ENSG00000100412 | aconitase 2 | -1.002811577 | 0.009679147 |
| ENSG00000097096 | synapse defective Rho GTPase homolog 2 | -1.003263393 | 0.034519762 |
| ENSG00000153922 | chromodomain helicase DNA binding protein 1 | -1.003952995 | 0.000408184 |
| ENSG00000196437 | zinc finger protein 569 | -1.004043746 | 9.28E-05 |
| ENSG00000151726 | acyl-CoA synthetase long-chain family member 1 | -1.004371443 | 0.003973084 |
| ENSG00000134909 | Rho GTPase activating protein 32 | -1.004984603 | 0.000773815 |
| ENSG00000196636 | succinate dehydrogenase complex assembly factor 3 | -1.005887581 | 0.002053589 |
| ENSG00000170027 | tyrosine 3-monooxygenase/tryptophan 5-monooxygenase activation protein gamma | -1.006011256 | 0.001527935 |
| ENSG00000123268 | activating transcription factor 1 | -1.006112809 | 0.009483234 |
| ENSG00000168538 | trafficking protein particle complex 11 | -1.006643784 | 9.00E-05 |
| ENSG00000135002 | riboflavin kinase | -1.00734362 | 0.000834941 |
| ENSG00000169116 | prostate androgen-regulated mucin-like protein 1 | -1.007844817 | 0.004189232 |
| ENSG00000149084 | hydroxysteroid 17-beta dehydrogenase 12 | -1.007975828 | 0.006328063 |
| ENSG00000185728 | YTH N6-methyladenosine RNA binding protein 3 | -1.008442884 | 0.002097573 |
| ENSG00000163635 | ataxin 7 | -1.009448067 | 0.000286792 |
| ENSG00000205765 | chromosome 5 open reading frame 51 | -1.009557591 | 0.000570355 |
| ENSG00000114302 | protein kinase cAMP-dependent type II regulatory subunit alpha | -1.010530639 | 0.000111285 |
| ENSG00000162664 | zinc finger protein 326 | -1.010871123 | 0.00065985 |
| ENSG00000068885 | intraflagellar transport 80 | -1.012130235 | 0.008508085 |
| ENSG00000136156 | integral membrane protein 2B | -1.013285974 | 0.002833842 |
| ENSG00000061987 | MON2 homolog, regulator of endosome-to-Golgi trafficking | -1.013300478 | 0.000120737 |
| ENSG00000196247 | zinc finger protein 107 | -1.014428249 | 0.017483652 |
| ENSG00000088387 | dedicator of cytokinesis 9 | -1.014757292 | 0.028962586 |
| ENSG00000151422 | FER tyrosine kinase | -1.015085884 | 0.000562453 |
| ENSG00000151692 | ring finger protein 144A | -1.015486736 | 0.00084985 |
| ENSG00000153310 | family with sequence similarity 49 member B | -1.015530872 | 0.003245635 |
| ENSG00000111224 | poly(ADP-ribose) polymerase family member 11 | -1.016307237 | 1.84E-05 |
| ENSG00000109790 | kelch like family member 5 | -1.016340258 | 0.001600608 |
| ENSG00000117528 | ATP binding cassette subfamily D member 3 | -1.016439536 | 0.000344257 |
| ENSG00000261609 | gigaxonin | -1.016501519 | 4.02E-05 |
| ENSG00000152382 | transcriptional adaptor 1 | -1.01652097 | 0.001529547 |
| ENSG00000112294 | aldehyde dehydrogenase 5 family member A1 | -1.016613684 | 0.000182851 |
| ENSG00000168172 | hook microtubule tethering protein 3 | -1.017324123 | 0.0004052 |
| ENSG00000132842 | adaptor related protein complex 3 beta 1 subunit | -1.019534755 | 4.80E-05 |
| ENSG00000180537 | ring finger protein 182 | -1.019909015 | 0.0025779 |
| ENSG00000166479 | thioredoxin related transmembrane protein 3 | -1.020375728 | 8.80E-05 |
| ENSG00000164163 | ATP binding cassette subfamily E member 1 | -1.020514757 | 0.000636541 |
| ENSG00000168216 | LMBR1 domain containing 1 | -1.02057653 | 0.002278803 |
| ENSG00000068383 | inositol polyphosphate-5-phosphatase A | -1.02147958 | 0.002804549 |
| ENSG00000170921 | tetratricopeptide repeat, ankyrin repeat and coiled-coil containing 2 | -1.022097163 | 0.000141355 |
| ENSG00000166450 | protogenin | -1.022587383 | 4.02E-05 |
| ENSG00000112893 | mannosidase alpha class 2A member 1 | -1.022819645 | 0.001593944 |
| ENSG00000179021 | chromosome 3 open reading frame 38 | -1.023979539 | 0.001073868 |
| ENSG00000122042 | ubiquitin like 3 | -1.024138917 | 0.000347082 |
| ENSG00000053770 | adaptor related protein complex 5 mu 1 subunit | -1.025893055 | 0.002241257 |
| ENSG00000071967 | cytochrome b reductase 1 | -1.026099805 | 7.87E-05 |
| ENSG00000137968 | solute carrier family 44 member 5 | -1.026984842 | 0.028847319 |
| ENSG00000136603 | SKI like proto-oncogene | -1.027626498 | 0.001460965 |
| ENSG00000137812 | kinetochore scaffold 1 | -1.027848842 | 0.000586837 |
| ENSG00000183044 | 4-aminobutyrate aminotransferase | -1.028344346 | 0.003499259 |
| ENSG00000114439 | BBX, HMG-box containing | -1.028506489 | 0.008664368 |
| ENSG00000165410 | cofilin 2 | -1.029102877 | 0.005228964 |
| ENSG00000128881 | tau tubulin kinase 2 | -1.02937279 | 0.000581081 |
| ENSG00000147874 | HAUS augmin like complex subunit 6 | -1.029645457 | 0.000431664 |
| ENSG00000178691 | SUZ12 polycomb repressive complex 2 subunit | -1.030684227 | 0.000795899 |
| ENSG00000142556 | zinc finger protein 614 | -1.030733868 | 0.004294437 |
| ENSG00000114573 | ATPase H+ transporting V1 subunit A | -1.030957564 | 0.005591391 |
| ENSG00000111276 | cyclin dependent kinase inhibitor 1B | -1.032352814 | 0.00136573 |
| ENSG00000130940 | castor zinc finger 1 | -1.033015193 | 2.49E-05 |
| ENSG00000158691 | zinc finger and SCAN domain containing 12 | -1.033979087 | 0.002511276 |
| ENSG00000092531 | synaptosome associated protein 23 | -1.034389978 | 0.001886098 |
| ENSG00000185973 | trimethyllysine hydroxylase, epsilon | -1.034926735 | 0.00853168 |
| ENSG00000138801 | 3'-phosphoadenosine 5'-phosphosulfate synthase 1 | -1.035657698 | 0.009802818 |
| ENSG00000166225 | fibroblast growth factor receptor substrate 2 | -1.035682402 | 0.000178456 |
| ENSG00000075539 | FRY like transcription coactivator | -1.035686538 | 7.85E-05 |
| ENSG00000112539 | chromosome 6 open reading frame 118 | -1.036700223 | 0.000138063 |
| ENSG00000157540 | dual specificity tyrosine phosphorylation regulated kinase 1A | -1.037991696 | 0.000121919 |
| ENSG00000145780 | fem-1 homolog C | -1.038234808 | 2.10E-05 |
| ENSG00000174738 | nuclear receptor subfamily 1 group D member 2 | -1.038270829 | 0.001961578 |
| ENSG00000143375 | cingulin | -1.039825971 | 0.032392646 |
| ENSG00000187257 | round spermatid basic protein 1 like | -1.040134034 | 0.001037623 |
| ENSG00000146263 | MMS22 like, DNA repair protein | -1.041001034 | 0.000287875 |
| ENSG00000071794 | helicase like transcription factor | -1.041479356 | 0.005537823 |
| ENSG00000196865 | NHL repeat containing 2 | -1.041570467 | 0.000357686 |
| ENSG00000060237 | WNK lysine deficient protein kinase 1 | -1.041832034 | 0.000237391 |
| ENSG00000138376 | BRCA1 associated RING domain 1 | -1.042914527 | 0.001576138 |
| ENSG00000203668 | CHM like, Rab escort protein 2 | -1.043029321 | 0.006842197 |
| ENSG00000171132 | protein kinase C epsilon | -1.04358615 | 0.011912498 |
| ENSG00000118762 | polycystin 2, transient receptor potential cation channel | -1.044000075 | 0.010276843 |
| ENSG00000162694 | exostosin like glycosyltransferase 2 | -1.044848867 | 0.000660022 |
| ENSG00000172071 | eukaryotic translation initiation factor 2 alpha kinase 3 | -1.045606141 | 0.013905895 |
| ENSG00000126464 | proline rich 12 | -1.045720219 | 0.00194094 |
| ENSG00000047315 | RNA polymerase II subunit B | -1.045729368 | 0.007454281 |
| ENSG00000123636 | bromodomain adjacent to zinc finger domain 2B | -1.045887478 | 0.000317732 |
| ENSG00000217128 | folliculin interacting protein 1 | -1.046055952 | 0.00079624 |
| ENSG00000108506 | integrator complex subunit 2 | -1.046077743 | 0.002377718 |
| ENSG00000146376 | Rho GTPase activating protein 18 | -1.046378123 | 0.000253634 |
| ENSG00000114120 | solute carrier family 25 member 36 | -1.047274111 | 0.009667798 |
| ENSG00000076053 | RNA binding motif protein 7 | -1.047567668 | 4.36E-05 |
| ENSG00000178974 | F-box protein 34 | -1.047766381 | 0.006496786 |
| ENSG00000115295 | CAP-Gly domain containing linker protein family member 4 | -1.048415544 | 0.003783637 |
| ENSG00000124789 | nucleoporin 153 | -1.048820307 | 0.0012875 |
| ENSG00000116406 | ER degradation enhancing alpha-mannosidase like protein 3 | -1.048939114 | 0.006374941 |
| ENSG00000138594 | tropomodulin 3 | -1.049106414 | 0.000408882 |
| ENSG00000182504 | centrosomal protein 97 | -1.04962243 | 0.001562093 |
| ENSG00000186272 | zinc finger protein 17 | -1.049817498 | 0.002983917 |
| ENSG00000177707 | nectin cell adhesion molecule 3 | -1.049841506 | 0.00634772 |
| ENSG00000198160 | MIER1 transcriptional regulator | -1.050188508 | 2.16E-05 |
| ENSG00000134852 | clock circadian regulator | -1.050569776 | 0.00014858 |
| ENSG00000126602 | TNF receptor associated protein 1 | -1.051129977 | 0.014928872 |
| ENSG00000077713 | solute carrier family 25 member 43 | -1.051515588 | 0.001225737 |
| ENSG00000186908 | zinc finger DHHC-type containing 17 | -1.05253994 | 0.002520468 |
| ENSG00000131931 | THAP domain containing 1 | -1.052869175 | 0.004183253 |
| ENSG00000155846 | PPARG coactivator 1 beta | -1.053216558 | 0.020166908 |
| ENSG00000156735 | BCL2 associated athanogene 4 | -1.053270441 | 0.004967007 |
| ENSG00000170448 | nuclear transcription factor, X-box binding like 1 | -1.053718689 | 0.03178748 |
| ENSG00000120262 | coiled-coil domain containing 170 | -1.053881801 | 0.024282169 |
| ENSG00000149308 | nuclear protein, coactivator of histone transcription | -1.054863491 | 0.001486392 |
| ENSG00000156958 | galactokinase 2 | -1.055352157 | 0.023623755 |
| ENSG00000092853 | claspin | -1.057164905 | 0.00012219 |
| ENSG00000083937 | charged multivesicular body protein 2B | -1.057250766 | 0.00153708 |
| ENSG00000206560 | ankyrin repeat domain 28 | -1.058767368 | 1.25E-05 |
| ENSG00000110429 | F-box protein 3 | -1.059303471 | 0.001180377 |
| ENSG00000157106 | SMG1, nonsense mediated mRNA decay associated PI3K related kinase | -1.059946055 | 0.001312359 |
| ENSG00000172465 | transcription elongation factor A like 1 | -1.059964025 | 0.002278458 |
| ENSG00000121892 | PDS5 cohesin associated factor A | -1.060048575 | 3.41E-05 |
| ENSG00000119969 | helicase, lymphoid-specific | -1.060107379 | 0.001288254 |
| ENSG00000182150 | ERCC excision repair 6 like 2 | -1.06133568 | 0.000406803 |
| ENSG00000133103 | component of oligomeric golgi complex 6 | -1.061953161 | 0.00485078 |
| ENSG00000119541 | vacuolar protein sorting 4 homolog B | -1.062699575 | 0.000483074 |
| ENSG00000177119 | anoctamin 6 | -1.063303045 | 0.001504505 |
| ENSG00000119729 | ras homolog family member Q | -1.063640074 | 0.006717533 |
| ENSG00000214029 | zinc finger protein 891 | -1.064106413 | 0.006740863 |
| ENSG00000137575 | syndecan binding protein | -1.064875186 | 0.007075333 |
| ENSG00000134352 | interleukin 6 signal transducer | -1.065469528 | 0.004336664 |
| ENSG00000140199 | solute carrier family 12 member 6 | -1.06614521 | 0.000503424 |
| ENSG00000203880 | protein-L-isoaspartate (D-aspartate) O-methyltransferase domain containing 2 | -1.066515888 | 0.001850238 |
| ENSG00000177917 | ADP ribosylation factor like GTPase 6 interacting protein 6 | -1.066786816 | 0.006723001 |
| ENSG00000136636 | potassium channel tetramerization domain containing 3 | -1.067007575 | 0.001601971 |
| ENSG00000124613 | zinc finger protein 391 | -1.067100123 | 0.00043697 |
| ENSG00000165672 | peroxiredoxin 3 | -1.067126093 | 0.000945808 |
| ENSG00000253251 |  | -1.067670557 | 0.013526107 |
| ENSG00000078114 | nebulette | -1.067848961 | 0.007585705 |
| ENSG00000123983 | acyl-CoA synthetase long-chain family member 3 | -1.068598939 | 8.43E-05 |
| ENSG00000112249 | activating signal cointegrator 1 complex subunit 3 | -1.069603939 | 0.000452672 |
| ENSG00000168300 | protein-L-isoaspartate (D-aspartate) O-methyltransferase domain containing 1 | -1.071392909 | 0.000461392 |
| ENSG00000164506 | syntaxin binding protein 5 | -1.071543062 | 0.003854089 |
| ENSG00000212907 | mitochondrially encoded NADH:ubiquinone oxidoreductase core subunit 4L | -1.073134187 | 0.017711973 |
| ENSG00000163322 | family with sequence similarity 175 member A | -1.074176348 | 0.000680382 |
| ENSG00000083123 | branched chain keto acid dehydrogenase E1 subunit beta | -1.074776678 | 9.01E-06 |
| ENSG00000129317 | pseudouridylate synthase 7 like | -1.075353825 | 0.00014112 |
| ENSG00000184203 | protein phosphatase 1 regulatory inhibitor subunit 2 | -1.075660413 | 0.00258666 |
| ENSG00000113391 | family with sequence similarity 172 member A | -1.075837858 | 0.000193538 |
| ENSG00000198569 | solute carrier family 34 member 3 | -1.076062029 | 0.030604673 |
| ENSG00000196208 | growth regulation by estrogen in breast cancer 1 | -1.076285234 | 0.001615079 |
| ENSG00000198887 | structural maintenance of chromosomes 5 | -1.076332634 | 0.000239165 |
| ENSG00000268043 | neuroblastoma breakpoint family member 12 | -1.076620486 | 0.006516269 |
| ENSG00000136874 | syntaxin 17 | -1.076732754 | 0.003507599 |
| ENSG00000060749 | glutamine and serine rich 1 | -1.078574559 | 0.003141638 |
| ENSG00000172340 | succinate-CoA ligase GDP-forming beta subunit | -1.078707166 | 0.001793145 |
| ENSG00000135766 | egl-9 family hypoxia inducible factor 1 | -1.078818636 | 0.000456142 |
| ENSG00000113580 | nuclear receptor subfamily 3 group C member 1 | -1.07891475 | 2.69E-05 |
| ENSG00000197223 | C1D nuclear receptor corepressor | -1.079006276 | 0.00206593 |
| ENSG00000055332 | eukaryotic translation initiation factor 2 alpha kinase 2 | -1.079549735 | 1.62E-05 |
| ENSG00000164342 | toll like receptor 3 | -1.079879121 | 0.019572686 |
| ENSG00000124496 | transcriptional regulating factor 1 | -1.08011019 | 5.18E-07 |
| ENSG00000243716 | nuclear pore complex interacting protein family member B5 | -1.080404099 | 0.009093421 |
| ENSG00000172493 | AF4/FMR2 family member 1 | -1.08089247 | 0.000292498 |
| ENSG00000132485 | zinc finger RANBP2-type containing 2 | -1.081508881 | 0.005097458 |
| ENSG00000109756 | Rap guanine nucleotide exchange factor 2 | -1.082467786 | 0.001210444 |
| ENSG00000168502 | microtubule crosslinking factor 1 | -1.083611263 | 0.000166915 |
| ENSG00000116539 | ASH1 like histone lysine methyltransferase | -1.084127196 | 0.001054082 |
| ENSG00000180488 | mitoguardin 1 | -1.084132912 | 0.000183772 |
| ENSG00000170776 | A-kinase anchoring protein 13 | -1.085821627 | 0.002047844 |
| ENSG00000167202 | TBC1 domain family member 2B | -1.086346689 | 0.018325044 |
| ENSG00000175893 | zinc finger DHHC-type containing 21 | -1.087440799 | 0.000736081 |
| ENSG00000109171 | SLAIN motif family member 2 | -1.08751303 | 3.82E-05 |
| ENSG00000067955 | core-binding factor beta subunit | -1.088243262 | 0.000832686 |
| ENSG00000144909 | oxysterol binding protein like 11 | -1.089404879 | 0.001277629 |
| ENSG00000075711 | discs large MAGUK scaffold protein 1 | -1.089540672 | 3.03E-06 |
| ENSG00000143401 | acidic nuclear phosphoprotein 32 family member E | -1.089705761 | 9.59E-05 |
| ENSG00000121481 | ring finger protein 2 | -1.089745118 | 0.000492083 |
| ENSG00000198890 | protein arginine methyltransferase 6 | -1.090379992 | 0.000498273 |
| ENSG00000152409 | junction mediating and regulatory protein, p53 cofactor | -1.091122903 | 0.000170492 |
| ENSG00000169504 | chloride intracellular channel 4 | -1.091910431 | 0.026621334 |
| ENSG00000213639 | protein phosphatase 1 catalytic subunit beta | -1.092118078 | 0.000388805 |
| ENSG00000092140 | G2/M-phase specific E3 ubiquitin protein ligase | -1.093739026 | 0.002010441 |
| ENSG00000083535 | progesterone immunomodulatory binding factor 1 | -1.094262934 | 3.60E-05 |
| ENSG00000064393 | homeodomain interacting protein kinase 2 | -1.094452115 | 0.007208394 |
| ENSG00000062370 | zinc finger protein 112 | -1.094812816 | 0.001645324 |
| ENSG00000120008 | WD repeat domain 11 | -1.094958474 | 0.000475183 |
| ENSG00000140157 | non imprinted in Prader-Willi/Angelman syndrome 2 | -1.097337996 | 0.021155249 |
| ENSG00000138346 | DNA replication helicase/nuclease 2 | -1.097416441 | 0.000854745 |
| ENSG00000187801 | ZFP69 zinc finger protein B | -1.098772868 | 0.024800597 |
| ENSG00000135272 | MyoD family inhibitor domain containing | -1.099009819 | 0.011118214 |
| ENSG00000136158 | sprouty RTK signaling antagonist 2 | -1.099414366 | 0.000994323 |
| ENSG00000143324 | xenotropic and polytropic retrovirus receptor 1 | -1.099636399 | 1.89E-06 |
| ENSG00000147592 | lactamase beta 2 | -1.099746294 | 0.029076282 |
| ENSG00000123106 | coiled-coil domain containing 91 | -1.102330747 | 0.000341916 |
| ENSG00000117906 | reticulocalbin 2 | -1.102484562 | 0.004252777 |
| ENSG00000187514 | prothymosin, alpha | -1.10253321 | 0.000248252 |
| ENSG00000114796 | kelch like family member 24 | -1.102943688 | 0.002278211 |
| ENSG00000133835 | hydroxysteroid 17-beta dehydrogenase 4 | -1.103524581 | 0.000837446 |
| ENSG00000124275 | 5-methyltetrahydrofolate-homocysteine methyltransferase reductase | -1.104559047 | 0.000433451 |
| ENSG00000120784 | ZFP30 zinc finger protein | -1.104778792 | 0.002372726 |
| ENSG00000095002 | mutS homolog 2 | -1.106104482 | 1.30E-05 |
| ENSG00000134138 | Meis homeobox 2 | -1.106203955 | 3.84E-06 |
| ENSG00000047346 | family with sequence similarity 214 member A | -1.106503732 | 0.000594228 |
| ENSG00000075568 | transmembrane protein 131 | -1.107040974 | 0.000118089 |
| ENSG00000163249 | cyclin Y like 1 | -1.107864673 | 0.004200869 |
| ENSG00000085433 | WD repeat domain 47 | -1.107936753 | 0.00452995 |
| ENSG00000106290 | TATA-box binding protein associated factor 6 | -1.108109048 | 0.024819635 |
| ENSG00000167447 | SMG8, nonsense mediated mRNA decay factor | -1.108629492 | 0.022186972 |
| ENSG00000162688 | amylo-alpha-1, 6-glucosidase, 4-alpha-glucanotransferase | -1.109070983 | 0.003980893 |
| ENSG00000180694 | transmembrane protein 64 | -1.109113296 | 0.005192785 |
| ENSG00000123219 | centromere protein K | -1.110137408 | 0.012591917 |
| ENSG00000120696 | kelch repeat and BTB domain containing 7 | -1.110719179 | 0.000997327 |
| ENSG00000165813 | coiled-coil domain containing 186 | -1.110737244 | 0.001393795 |
| ENSG00000146243 | interleukin 1 receptor associated kinase 1 binding protein 1 | -1.111043086 | 0.000368483 |
| ENSG00000140992 | 3-phosphoinositide dependent protein kinase 1 | -1.11141441 | 0.006023897 |
| ENSG00000008282 | synaptophysin like 1 | -1.111549147 | 0.000219405 |
| ENSG00000169155 | zinc finger and BTB domain containing 43 | -1.111597349 | 0.002315291 |
| ENSG00000213551 | DnaJ heat shock protein family (Hsp40) member C9 | -1.111731638 | 0.011804082 |
| ENSG00000174405 | DNA ligase 4 | -1.111776182 | 0.027274151 |
| ENSG00000146350 | TBC1 domain family member 32 | -1.112329989 | 0.029731494 |
| ENSG00000275052 | protein phosphatase 4 regulatory subunit 3B | -1.112625638 | 5.25E-06 |
| ENSG00000162105 | SH3 and multiple ankyrin repeat domains 2 | -1.112905642 | 0.011081712 |
| ENSG00000069956 | mitogen-activated protein kinase 6 | -1.113413813 | 0.00046883 |
| ENSG00000158711 | ELK4, ETS transcription factor | -1.113724568 | 1.49E-05 |
| ENSG00000113231 | phosphodiesterase 8B | -1.113958086 | 1.49E-05 |
| ENSG00000215114 | UBX domain protein 2B | -1.114625893 | 1.24E-06 |
| ENSG00000197535 | myosin VA | -1.115037986 | 7.70E-05 |
| ENSG00000157578 | LCA5L, lebercilin like | -1.115228862 | 0.000237532 |
| ENSG00000188419 | CHM, Rab escort protein 1 | -1.115236983 | 6.74E-05 |
| ENSG00000141198 | target of myb1 like 1 membrane trafficking protein | -1.116079269 | 0.004563246 |
| ENSG00000113441 | leucyl and cystinyl aminopeptidase | -1.117174936 | 0.000127821 |
| ENSG00000187240 | dynein cytoplasmic 2 heavy chain 1 | -1.118071579 | 0.000505343 |
| ENSG00000108946 | protein kinase cAMP-dependent type I regulatory subunit alpha | -1.118831696 | 0.000356276 |
| ENSG00000176105 | YES proto-oncogene 1, Src family tyrosine kinase | -1.119000358 | 0.000437832 |
| ENSG00000000460 | chromosome 1 open reading frame 112 | -1.119204215 | 0.001013746 |
| ENSG00000180233 | zinc and ring finger 2 | -1.119219868 | 0.000463129 |
| ENSG00000038219 | biorientation of chromosomes in cell division 1 like 1 | -1.119465956 | 0.006447801 |
| ENSG00000115355 | coiled-coil domain containing 88A | -1.119774672 | 2.18E-05 |
| ENSG00000146476 | acidic residue methyltransferase 1 | -1.121171618 | 0.008380488 |
| ENSG00000139517 | ligand of numb-protein X 2 | -1.121329285 | 0.009212979 |
| ENSG00000187097 | ectonucleoside triphosphate diphosphohydrolase 5 | -1.122647213 | 0.001963924 |
| ENSG00000164329 | poly(A) RNA polymerase D4, non-canonical | -1.123116948 | 0.004865667 |
| ENSG00000055147 | family with sequence similarity 114 member A2 | -1.123253434 | 2.54E-05 |
| ENSG00000122591 | family with sequence similarity 126 member A | -1.123355668 | 3.63E-05 |
| ENSG00000129493 | HEAT repeat containing 5A | -1.124485304 | 0.001093968 |
| ENSG00000140367 | ubiquitin conjugating enzyme E2 Q2 | -1.125875535 | 0.000584106 |
| ENSG00000203965 | EF-hand calcium binding domain 7 | -1.126397739 | 0.000421038 |
| ENSG00000166444 | suppression of tumorigenicity 5 | -1.127407791 | 0.034430555 |
| ENSG00000215301 | DEAD-box helicase 3, X-linked | -1.128414775 | 0.005781627 |
| ENSG00000164300 | serine incorporator 5 | -1.128612123 | 0.002115637 |
| ENSG00000128346 | chromosome 22 open reading frame 23 | -1.129087905 | 0.011544589 |
| ENSG00000146278 | proline rich nuclear receptor coactivator 1 | -1.12912745 | 8.80E-05 |
| ENSG00000146247 | pleckstrin homology domain interacting protein | -1.130013807 | 0.001598263 |
| ENSG00000185238 | protein arginine methyltransferase 3 | -1.130128474 | 0.011338712 |
| ENSG00000144674 | golgin A4 | -1.130445823 | 0.001512618 |
| ENSG00000168438 | cell division cycle 40 | -1.130557627 | 0.005520494 |
| ENSG00000099250 | neuropilin 1 | -1.131543638 | 0.001714318 |
| ENSG00000107679 | pleckstrin homology domain containing A1 | -1.131621802 | 0.013141062 |
| ENSG00000198162 | mannosidase alpha class 1A member 2 | -1.132037794 | 3.87E-08 |
| ENSG00000197969 | vacuolar protein sorting 13 homolog A | -1.132109388 | 0.004090406 |
| ENSG00000185697 | MYB proto-oncogene like 1 | -1.132630157 | 0.001552907 |
| ENSG00000175073 | valosin containing protein interacting protein 1 | -1.132842738 | 2.02E-05 |
| ENSG00000095574 | IKAROS family zinc finger 5 | -1.133964593 | 0.000461665 |
| ENSG00000115159 | glycerol-3-phosphate dehydrogenase 2 | -1.135697438 | 0.00092327 |
| ENSG00000183354 | KIAA2026 | -1.136149381 | 0.000109427 |
| ENSG00000109572 | chloride voltage-gated channel 3 | -1.136741269 | 1.19E-05 |
| ENSG00000083642 | PDS5 cohesin associated factor B | -1.137294428 | 3.41E-06 |
| ENSG00000266173 | STE20-related kinase adaptor alpha | -1.138137322 | 0.013074529 |
| ENSG00000116473 | RAP1A, member of RAS oncogene family | -1.138364542 | 8.91E-05 |
| ENSG00000169252 | adrenoceptor beta 2 | -1.138931819 | 0.009164991 |
| ENSG00000234444 | zinc finger protein 736 | -1.139687588 | 0.003329882 |
| ENSG00000048405 | zinc finger protein 800 | -1.140729752 | 0.00061548 |
| ENSG00000198900 | topoisomerase (DNA) I | -1.141181106 | 0.003538659 |
| ENSG00000111911 | histidine triad nucleotide binding protein 3 | -1.141522777 | 0.003843912 |
| ENSG00000113615 | SEC24 homolog A, COPII coat complex component | -1.142103223 | 0.010128309 |
| ENSG00000145241 | centromere protein C | -1.142388246 | 0.000276641 |
| ENSG00000172007 | RAB33B, member RAS oncogene family | -1.142395132 | 0.005004409 |
| ENSG00000185305 | ADP ribosylation factor like GTPase 15 | -1.142799349 | 0.000138982 |
| ENSG00000173068 | basonuclin 2 | -1.142902558 | 0.014649269 |
| ENSG00000142453 | coactivator associated arginine methyltransferase 1 | -1.143293655 | 0.017432889 |
| ENSG00000197619 | zinc finger protein 615 | -1.144324174 | 0.00042801 |
| ENSG00000115419 | glutaminase | -1.145141254 | 0.004947298 |
| ENSG00000177463 | nuclear receptor subfamily 2 group C member 2 | -1.146751688 | 0.01821624 |
| ENSG00000096654 | zinc finger protein 184 | -1.146922183 | 0.000737821 |
| ENSG00000005810 | MYC binding protein 2, E3 ubiquitin protein ligase | -1.149003337 | 0.000267833 |
| ENSG00000134371 | cell division cycle 73 | -1.149471882 | 0.000460952 |
| ENSG00000165997 | ADP ribosylation factor like GTPase 5B | -1.150484036 | 8.36E-05 |
| ENSG00000126778 | SIX homeobox 1 | -1.151375462 | 1.88E-08 |
| ENSG00000175322 | zinc finger protein 519 | -1.151953001 | 0.00568029 |
| ENSG00000105835 | nicotinamide phosphoribosyltransferase | -1.152456047 | 0.001282209 |
| ENSG00000139436 | GIT ArfGAP 2 | -1.152724462 | 0.000260252 |
| ENSG00000144228 | speckle type BTB/POZ protein like | -1.153761996 | 0.00091972 |
| ENSG00000125257 | ATP binding cassette subfamily C member 4 | -1.153933476 | 0.001022369 |
| ENSG00000164031 | DnaJ heat shock protein family (Hsp40) member B14 | -1.15396853 | 0.000645099 |
| ENSG00000166860 | zinc finger and BTB domain containing 39 | -1.154598822 | 0.000383701 |
| ENSG00000162959 | mediator of cell motility 1 | -1.15516615 | 0.035425459 |
| ENSG00000083312 | transportin 1 | -1.155325181 | 2.08E-05 |
| ENSG00000198589 | LPS responsive beige-like anchor protein | -1.155627864 | 0.000443496 |
| ENSG00000001631 | KRIT1, ankyrin repeat containing | -1.155684193 | 0.001210546 |
| ENSG00000166734 | cancer susceptibility candidate 4 | -1.155873822 | 2.82E-05 |
| ENSG00000030066 | nucleoporin 160 | -1.156107514 | 0.001666028 |
| ENSG00000111731 | C2 calcium dependent domain containing 5 | -1.15718525 | 0.001800531 |
| ENSG00000138764 | cyclin G2 | -1.157209808 | 0.000531461 |
| ENSG00000174514 | major facilitator superfamily domain containing 4A | -1.158294184 | 0.016179443 |
| ENSG00000123124 | WW domain containing E3 ubiquitin protein ligase 1 | -1.158368477 | 0.000910806 |
| ENSG00000226650 | kinesin family member 4B | -1.158714498 | 0.028604746 |
| ENSG00000167548 | lysine methyltransferase 2D | -1.158898378 | 0.006186103 |
| ENSG00000234602 | multiciliate differentiation and DNA synthesis associated cell cycle protein | -1.15900706 | 0.012231328 |
| ENSG00000127081 | zinc finger protein 484 | -1.159313953 | 0.004268382 |
| ENSG00000076554 | tumor protein D52 | -1.160767112 | 1.30E-05 |
| ENSG00000109738 | glycine receptor beta | -1.160910942 | 0.006874738 |
| ENSG00000146757 | zinc finger protein 92 | -1.161700516 | 0.010540851 |
| ENSG00000012174 | membrane bound transcription factor peptidase, site 2 | -1.162486296 | 0.001162813 |
| ENSG00000150995 | inositol 1,4,5-trisphosphate receptor type 1 | -1.162637915 | 0.00631202 |
| ENSG00000085382 | HECT domain and ankyrin repeat containing E3 ubiquitin protein ligase 1 | -1.163171398 | 0.008260621 |
| ENSG00000109680 | TBC1 domain family member 19 | -1.163701619 | 0.004712771 |
| ENSG00000043093 | defective in cullin neddylation 1 domain containing 1 | -1.163724042 | 0.000979713 |
| ENSG00000124882 | epiregulin | -1.16395749 | 0.003354764 |
| ENSG00000163625 | WD repeat and FYVE domain containing 3 | -1.1642639 | 0.000653107 |
| ENSG00000089123 | taspase 1 | -1.164638955 | 0.018289635 |
| ENSG00000172977 | lysine acetyltransferase 5 | -1.165623888 | 0.008552726 |
| ENSG00000155330 | chromosome 16 open reading frame 87 | -1.166196374 | 0.000388331 |
| ENSG00000171016 | pygopus family PHD finger 1 | -1.167342656 | 0.007355338 |
| ENSG00000146411 | solute carrier family 2 member 12 | -1.167402043 | 0.00353202 |
| ENSG00000135378 | proline rich and Gla domain 4 | -1.167543762 | 0.010819909 |
| ENSG00000139117 | copine 8 | -1.167617599 | 0.015387767 |
| ENSG00000109787 | Kruppel like factor 3 | -1.168525832 | 4.71E-05 |
| ENSG00000130962 | proline rich and Gla domain 1 | -1.168689738 | 0.024081834 |
| ENSG00000119636 | basal body orientation factor 1 | -1.169143327 | 0.011733888 |
| ENSG00000115866 | aspartyl-tRNA synthetase | -1.169196625 | 0.003904013 |
| ENSG00000135776 | ATP binding cassette subfamily B member 10 | -1.169857874 | 0.005358619 |
| ENSG00000196792 | striatin 3 | -1.171508161 | 0.004054806 |
| ENSG00000197603 | chromosome 5 open reading frame 42 | -1.171656605 | 3.44E-05 |
| ENSG00000129515 | sorting nexin 6 | -1.17169743 | 0.004729077 |
| ENSG00000105856 | HMG-box transcription factor 1 | -1.171935434 | 0.00019104 |
| ENSG00000011258 | mbt domain containing 1 | -1.17364368 | 0.002196243 |
| ENSG00000111877 | minichromosome maintenance 9 homologous recombination repair factor | -1.173719031 | 0.000131461 |
| ENSG00000171103 | tRNA methyltransferase 61B | -1.173740182 | 0.020466545 |
| ENSG00000152270 | phosphodiesterase 3B | -1.17489691 | 0.028114532 |
| ENSG00000139350 | neural precursor cell expressed, developmentally down-regulated 1 | -1.175142653 | 0.000170948 |
| ENSG00000164463 | CREB3 regulatory factor | -1.175346258 | 0.00015601 |
| ENSG00000108239 | TBC1 domain family member 12 | -1.176184033 | 0.001478708 |
| ENSG00000172262 | zinc finger protein 131 | -1.176268395 | 0.000728448 |
| ENSG00000120256 | LDL receptor related protein 11 | -1.176834616 | 0.001295371 |
| ENSG00000166200 | COP9 signalosome subunit 2 | -1.177273404 | 0.000579173 |
| ENSG00000054267 | AT-rich interaction domain 4B | -1.177361424 | 0.000291528 |
| ENSG00000005483 | lysine methyltransferase 2E | -1.177945143 | 0.00030675 |
| ENSG00000204179 | protein tyrosine phosphatase, non-receptor type 20 | -1.178417306 | 0.004873309 |
| ENSG00000156011 | pleckstrin and Sec7 domain containing 3 | -1.179516799 | 0.009118851 |
| ENSG00000145715 | RAS p21 protein activator 1 | -1.180497356 | 0.0080213 |
| ENSG00000165525 | nuclear export mediator factor | -1.182217187 | 1.34E-05 |
| ENSG00000163961 | ring finger protein 168 | -1.182375626 | 0.000197871 |
| ENSG00000197372 | zinc finger protein 675 | -1.183409456 | 0.003477551 |
| ENSG00000143970 | additional sex combs like 2, transcriptional regulator | -1.18362736 | 9.35E-05 |
| ENSG00000211455 | serine/threonine kinase 38 like | -1.183901912 | 8.14E-05 |
| ENSG00000065615 | cytochrome b5 reductase 4 | -1.18447578 | 0.000374304 |
| ENSG00000250312 | zinc finger protein 718 | -1.185165935 | 0.003877839 |
| ENSG00000112742 | TTK protein kinase | -1.18522009 | 9.64E-05 |
| ENSG00000180447 | growth arrest specific 1 | -1.187821137 | 0.001683286 |
| ENSG00000172766 | N(alpha)-acetyltransferase 16, NatA auxiliary subunit | -1.188304812 | 0.001180123 |
| ENSG00000186260 | MKL1/myocardin like 2 | -1.188518884 | 0.000103682 |
| ENSG00000164068 | ring finger protein 123 | -1.189101146 | 0.019637345 |
| ENSG00000106723 | spindlin 1 | -1.189726674 | 9.49E-05 |
| ENSG00000157212 | PAX interacting protein 1 | -1.190257973 | 0.000396556 |
| ENSG00000169193 | coiled-coil domain containing 126 | -1.190271004 | 0.025900732 |
| ENSG00000173926 | membrane associated ring-CH-type finger 3 | -1.190344059 | 0.00660038 |
| ENSG00000165322 | Rho GTPase activating protein 12 | -1.192560424 | 1.68E-05 |
| ENSG00000007923 | DnaJ heat shock protein family (Hsp40) member C11 | -1.193313304 | 0.009080769 |
| ENSG00000151338 | mirror-image polydactyly 1 | -1.194091095 | 0.026520554 |
| ENSG00000001084 | glutamate-cysteine ligase catalytic subunit | -1.194133228 | 0.000162564 |
| ENSG00000119778 | ATPase family, AAA domain containing 2B | -1.194883935 | 2.51E-05 |
| ENSG00000065613 | STE20 like kinase | -1.196059276 | 1.94E-06 |
| ENSG00000126945 | heterogeneous nuclear ribonucleoprotein H2 (H') | -1.196702705 | 0.000716129 |
| ENSG00000158987 | Rap guanine nucleotide exchange factor 6 | -1.196805755 | 0.001758258 |
| ENSG00000151881 | transmembrane protein 267 | -1.19680982 | 0.005816544 |
| ENSG00000165732 | DExD-box helicase 21 | -1.197122263 | 0.00819391 |
| ENSG00000153339 | trafficking protein particle complex 8 | -1.197203297 | 0.000264248 |
| ENSG00000176244 | acyl-CoA binding domain containing 7 | -1.19736193 | 0.027774735 |
| ENSG00000254598 | casein kinase 2 alpha 3 | -1.197610886 | 0.031343022 |
| ENSG00000114541 | FERM domain containing 4B | -1.197619535 | 0.023631002 |
| ENSG00000149313 | aminoadipate-semialdehyde dehydrogenase-phosphopantetheinyl transferase | -1.197936477 | 0.012599357 |
| ENSG00000115966 | activating transcription factor 2 | -1.198276227 | 0.001676485 |
| ENSG00000165730 | storkhead box 1 | -1.198799864 | 0.00157153 |
| ENSG00000164144 | ADP ribosylation factor interacting protein 1 | -1.200224973 | 0.00028565 |
| ENSG00000139826 | abhydrolase domain containing 13 | -1.200660658 | 0.00034649 |
| ENSG00000123575 | family with sequence similarity 199, X-linked | -1.201240158 | 4.43E-05 |
| ENSG00000169047 | insulin receptor substrate 1 | -1.201302357 | 0.000384095 |
| ENSG00000136143 | succinate-CoA ligase ADP-forming beta subunit | -1.201315901 | 0.001630014 |
| ENSG00000122008 | DNA polymerase kappa | -1.202508307 | 0.000377495 |
| ENSG00000113282 | clathrin interactor 1 | -1.202843067 | 0.005594112 |
| ENSG00000102081 | fragile X mental retardation 1 | -1.203388401 | 0.000134589 |
| ENSG00000177034 | metaxin 3 | -1.204439707 | 1.08E-05 |
| ENSG00000165097 | lysine demethylase 1B | -1.205421125 | 4.65E-05 |
| ENSG00000106701 | fibronectin type III and SPRY domain containing 1 like | -1.205429981 | 0.006999736 |
| ENSG00000061676 | NCK associated protein 1 | -1.205779813 | 0.000515395 |
| ENSG00000145375 | spermatogenesis associated 5 | -1.205968819 | 0.000141416 |
| ENSG00000160218 | trafficking protein particle complex 10 | -1.206008932 | 0.010432502 |
| ENSG00000118276 | beta-1,4-galactosyltransferase 6 | -1.206396522 | 0.001082836 |
| ENSG00000196418 | zinc finger protein 124 | -1.208981055 | 0.003593716 |
| ENSG00000122970 | intraflagellar transport 81 | -1.209525217 | 0.003787683 |
| ENSG00000089177 | kinesin family member 16B | -1.210315461 | 0.001306338 |
| ENSG00000166483 | WEE1 G2 checkpoint kinase | -1.210894202 | 0.004221688 |
| ENSG00000174891 | arginine and serine rich coiled-coil 1 | -1.210901472 | 0.001129551 |
| ENSG00000144224 | UBX domain protein 4 | -1.21215424 | 6.96E-05 |
| ENSG00000204217 | bone morphogenetic protein receptor type 2 | -1.212299662 | 8.16E-06 |
| ENSG00000112290 | WAS protein family member 1 | -1.213357477 | 0.000675198 |
| ENSG00000171100 | myotubularin 1 | -1.213405744 | 0.001607706 |
| ENSG00000089048 | ESF1 nucleolar pre-rRNA processing protein homolog | -1.214430821 | 0.001250533 |
| ENSG00000173889 | polyhomeotic homolog 3 | -1.214561967 | 0.003322901 |
| ENSG00000051825 | M-phase phosphoprotein 9 | -1.215101718 | 7.12E-05 |
| ENSG00000103540 | centriolar coiled-coil protein 110 | -1.215108079 | 0.000393584 |
| ENSG00000138380 | calcium responsive transcription factor | -1.215975026 | 0.01001217 |
| ENSG00000162616 | DnaJ heat shock protein family (Hsp40) member B4 | -1.216262657 | 0.000228199 |
| ENSG00000092439 | transient receptor potential cation channel subfamily M member 7 | -1.217919261 | 0.003222285 |
| ENSG00000169019 | COMM domain containing 8 | -1.217934738 | 0.000903122 |
| ENSG00000131470 | PSMC3 interacting protein | -1.218015738 | 0.00274212 |
| ENSG00000131375 | calpain 7 | -1.218713808 | 0.000163832 |
| ENSG00000124198 | ADP ribosylation factor guanine nucleotide exchange factor 2 | -1.220471046 | 0.007783652 |
| ENSG00000178074 | chromosome 2 open reading frame 69 | -1.221498512 | 0.001038042 |
| ENSG00000198945 | l(3)mbt-like 3 (Drosophila) | -1.223427568 | 0.003362154 |
| ENSG00000121957 | G-protein signaling modulator 2 | -1.223576735 | 1.46E-05 |
| ENSG00000137075 | ring finger protein 38 | -1.223675887 | 7.93E-07 |
| ENSG00000172869 | Dmx like 1 | -1.223966862 | 4.22E-06 |
| ENSG00000107669 | arginyltransferase 1 | -1.224420298 | 8.91E-05 |
| ENSG00000136108 | cytoskeleton associated protein 2 | -1.224668628 | 0.000529512 |
| ENSG00000004766 | VPS50, EARP/GARPII complex subunit | -1.225258859 | 8.70E-05 |
| ENSG00000116574 | ras homolog family member U | -1.225614867 | 0.011620783 |
| ENSG00000115183 | tetratricopeptide repeat, ankyrin repeat and coiled-coil containing 1 | -1.226855179 | 0.002530586 |
| ENSG00000113384 | golgi phosphoprotein 3 | -1.227117063 | 0.000748577 |
| ENSG00000116106 | EPH receptor A4 | -1.227499499 | 0.000171142 |
| ENSG00000121931 | ligand dependent nuclear receptor interacting factor 1 | -1.22767133 | 0.002646293 |
| ENSG00000157500 | adaptor protein, phosphotyrosine interacting with PH domain and leucine zipper 1 | -1.228656531 | 0.000249713 |
| ENSG00000153317 | ArfGAP with SH3 domain, ankyrin repeat and PH domain 1 | -1.229133545 | 0.001906712 |
| ENSG00000196535 | myosin XVIIIA | -1.229213827 | 0.005171098 |
| ENSG00000130150 | motile sperm domain containing 2 | -1.230083043 | 0.004586604 |
| ENSG00000126804 | zinc finger and BTB domain containing 1 | -1.230165294 | 0.003676074 |
| ENSG00000056097 | zinc finger RNA binding protein | -1.230782902 | 7.89E-05 |
| ENSG00000104442 | armadillo repeat containing 1 | -1.231152966 | 0.001747046 |
| ENSG00000214194 | long intergenic non-protein coding RNA 998 | -1.231284461 | 6.16E-05 |
| ENSG00000205476 | coiled-coil domain containing 85C | -1.23173853 | 0.015539523 |
| ENSG00000198142 | sosondowah ankyrin repeat domain family member C | -1.231810046 | 0.000914501 |
| ENSG00000153130 | short coiled-coil protein | -1.233078114 | 0.00012105 |
| ENSG00000005020 | src kinase associated phosphoprotein 2 | -1.233628961 | 6.44E-05 |
| ENSG00000140937 | cadherin 11 | -1.233691434 | 0.013644833 |
| ENSG00000177189 | ribosomal protein S6 kinase A3 | -1.233987904 | 0.007986675 |
| ENSG00000107581 | eukaryotic translation initiation factor 3 subunit A | -1.234086852 | 0.001541555 |
| ENSG00000112851 | erbb2 interacting protein | -1.234154272 | 2.53E-06 |
| ENSG00000123094 | Ras association domain family member 8 | -1.235102837 | 1.03E-07 |
| ENSG00000181904 | chromosome 5 open reading frame 24 | -1.235528963 | 0.000365595 |
| ENSG00000197329 | pellino E3 ubiquitin protein ligase 1 | -1.236063214 | 7.96E-05 |
| ENSG00000116747 | TROVE domain family member 2 | -1.237274201 | 0.000269447 |
| ENSG00000131845 | zinc finger protein 304 | -1.237314464 | 0.001527837 |
| ENSG00000133111 | regulatory factor X associated protein | -1.238307006 | 0.0060644 |
| ENSG00000198265 | helicase with zinc finger | -1.23966161 | 4.61E-05 |
| ENSG00000005812 | F-box and leucine rich repeat protein 3 | -1.241644102 | 0.001282927 |
| ENSG00000138587 | meiosis specific nuclear structural 1 | -1.241972254 | 0.021705843 |
| ENSG00000082269 | family with sequence similarity 135 member A | -1.242190691 | 0.010431604 |
| ENSG00000175161 | cell adhesion molecule 2 | -1.244200964 | 0.00163683 |
| ENSG00000148158 | sorting nexin family member 30 | -1.244744601 | 0.000593973 |
| ENSG00000146574 | CCZ1 homolog B, vacuolar protein trafficking and biogenesis associated | -1.244912201 | 0.032323801 |
| ENSG00000198586 | tousled like kinase 1 | -1.245124254 | 3.59E-05 |
| ENSG00000103549 | ring finger protein 40 | -1.246919978 | 0.016361527 |
| ENSG00000077454 | leucine rich repeats and calponin homology domain containing 4 | -1.247055483 | 0.014104797 |
| ENSG00000131788 | protein inhibitor of activated STAT 3 | -1.24821913 | 0.007130164 |
| ENSG00000151458 | ankyrin repeat domain 50 | -1.248381612 | 0.000520566 |
| ENSG00000169826 | chondroitin sulfate N-acetylgalactosaminyltransferase 2 | -1.248876396 | 0.008748107 |
| ENSG00000176853 | family with sequence similarity 91 member A1 | -1.249727632 | 0.000143048 |
| ENSG00000189042 | zinc finger protein 567 | -1.25352078 | 0.008152685 |
| ENSG00000189144 | zinc finger protein 573 | -1.256941045 | 0.022118283 |
| ENSG00000158092 | NCK adaptor protein 1 | -1.257486295 | 0.007891605 |
| ENSG00000105357 | myosin heavy chain 14 | -1.258403067 | 0.019588409 |
| ENSG00000146859 | transmembrane protein 140 | -1.260352611 | 0.006336771 |
| ENSG00000065833 | malic enzyme 1 | -1.261454712 | 0.001126502 |
| ENSG00000119630 | placental growth factor | -1.262522516 | 0.007027925 |
| ENSG00000177853 | zinc finger protein 518A | -1.26356761 | 0.000401653 |
| ENSG00000041353 | RAB27B, member RAS oncogene family | -1.263884743 | 0.000390563 |
| ENSG00000177200 | chromodomain helicase DNA binding protein 9 | -1.264590637 | 8.75E-06 |
| ENSG00000181827 | regulatory factor X7 | -1.265731611 | 0.002712744 |
| ENSG00000111647 | UHRF1 binding protein 1 like | -1.265841062 | 1.47E-05 |
| ENSG00000177409 | sterile alpha motif domain containing 9 like | -1.265918496 | 0.003960537 |
| ENSG00000068366 | acyl-CoA synthetase long-chain family member 4 | -1.266866755 | 0.002572289 |
| ENSG00000168016 | tetratricopeptide repeat and ankyrin repeat containing 1 | -1.267202054 | 0.001198284 |
| ENSG00000163281 | glucosamine-6-phosphate deaminase 2 | -1.267409807 | 0.001087328 |
| ENSG00000170264 | family with sequence similarity 161 member A | -1.268404062 | 0.013749344 |
| ENSG00000170185 | ubiquitin specific peptidase 38 | -1.270256426 | 0.000181237 |
| ENSG00000066422 | zinc finger and BTB domain containing 11 | -1.270754495 | 0.006499307 |
| ENSG00000082212 | malic enzyme 2 | -1.272050782 | 5.80E-05 |
| ENSG00000161654 | LSM12 homolog | -1.272449958 | 0.008672946 |
| ENSG00000114127 | 5'-3' exoribonuclease 1 | -1.272564307 | 3.04E-06 |
| ENSG00000056277 | zinc finger protein 280C | -1.272769874 | 0.00199903 |
| ENSG00000213160 | kelch like family member 23 | -1.272902285 | 0.001164869 |
| ENSG00000102699 | poly(ADP-ribose) polymerase family member 4 | -1.273500581 | 0.00065655 |
| ENSG00000115084 | solute carrier family 35 member F5 | -1.273802826 | 0.003012378 |
| ENSG00000138081 | F-box protein 11 | -1.274007104 | 0.000685864 |
| ENSG00000182621 | phospholipase C beta 1 | -1.27474404 | 0.004897403 |
| ENSG00000196950 | solute carrier family 39 member 10 | -1.274786279 | 0.017705136 |
| ENSG00000101972 | stromal antigen 2 | -1.276244532 | 4.21E-05 |
| ENSG00000122482 | zinc finger protein 644 | -1.277159684 | 6.36E-06 |
| ENSG00000135535 | CD164 molecule | -1.277474479 | 0.000665753 |
| ENSG00000118997 | dynein axonemal heavy chain 7 | -1.278097769 | 0.002793684 |
| ENSG00000166974 | microtubule associated protein RP/EB family member 2 | -1.278943143 | 0.006069083 |
| ENSG00000111554 | Mdm1 nuclear protein | -1.279027197 | 0.003024671 |
| ENSG00000139163 | ethanolamine kinase 1 | -1.279382972 | 0.000139039 |
| ENSG00000154727 | GA binding protein transcription factor alpha subunit | -1.279526478 | 2.13E-05 |
| ENSG00000133657 | ATPase 13A3 | -1.280066576 | 0.001365957 |
| ENSG00000131389 | solute carrier family 6 member 6 | -1.28028514 | 0.000792487 |
| ENSG00000163743 | ring finger and CHY zinc finger domain containing 1 | -1.280654462 | 0.002706174 |
| ENSG00000138119 | myoferlin | -1.282353072 | 7.46E-05 |
| ENSG00000067113 | phospholipid phosphatase 1 | -1.283796099 | 0.000311176 |
| ENSG00000124333 | vesicle associated membrane protein 7 | -1.284842631 | 0.001195656 |
| ENSG00000196914 | Rho guanine nucleotide exchange factor 12 | -1.28544461 | 0.000281178 |
| ENSG00000176624 | mex-3 RNA binding family member C | -1.286273423 | 0.000962987 |
| ENSG00000156273 | BTB domain and CNC homolog 1 | -1.286645842 | 0.0002591 |
| ENSG00000155744 | family with sequence similarity 126 member B | -1.287503436 | 0.000240808 |
| ENSG00000101745 | ankyrin repeat domain 12 | -1.288504882 | 0.000109278 |
| ENSG00000187189 | TSPY like 4 | -1.28962649 | 0.000975075 |
| ENSG00000136783 | nipsnap homolog 3A | -1.289815991 | 8.32E-05 |
| ENSG00000144306 | secernin 3 | -1.292881367 | 1.39E-06 |
| ENSG00000196313 | POM121 transmembrane nucleoporin | -1.29511396 | 0.010112438 |
| ENSG00000164796 | CUB and Sushi multiple domains 3 | -1.295124494 | 0.007295934 |
| ENSG00000178177 | ligand dependent nuclear receptor corepressor like | -1.298025542 | 3.00E-05 |
| ENSG00000140262 | transcription factor 12 | -1.298191385 | 0.000260862 |
| ENSG00000143418 | ceramide synthase 2 | -1.29844772 | 0.018269282 |
| ENSG00000122545 | septin 7 | -1.298871568 | 9.28E-05 |
| ENSG00000100784 | ribosomal protein S6 kinase A5 | -1.299068199 | 4.14E-06 |
| ENSG00000157625 | TGF-beta activated kinase 1/MAP3K7 binding protein 3 | -1.299476884 | 7.97E-05 |
| ENSG00000151575 | testis expressed 9 | -1.300456625 | 0.000718546 |
| ENSG00000115415 | signal transducer and activator of transcription 1 | -1.303545287 | 1.97E-05 |
| ENSG00000116171 | sterol carrier protein 2 | -1.305496571 | 1.26E-05 |
| ENSG00000115594 | interleukin 1 receptor type 1 | -1.305527287 | 7.07E-06 |
| ENSG00000114480 | 1,4-alpha-glucan branching enzyme 1 | -1.305624094 | 0.000374318 |
| ENSG00000113658 | SMAD family member 5 | -1.306450199 | 0.000218642 |
| ENSG00000164244 | proline rich coiled-coil 1 | -1.307015317 | 1.92E-05 |
| ENSG00000096717 | sirtuin 1 | -1.307683716 | 0.000469135 |
| ENSG00000153561 | required for meiotic nuclear division 5 homolog A | -1.308530909 | 0.000311927 |
| ENSG00000031691 | centromere protein Q | -1.308733466 | 0.000621885 |
| ENSG00000163611 | spindle and centriole associated protein 1 | -1.309702008 | 0.000830565 |
| ENSG00000082996 | ring finger protein 13 | -1.311080042 | 0.002380945 |
| ENSG00000171757 | leucine rich repeat containing 34 | -1.312282247 | 0.024753658 |
| ENSG00000106688 | solute carrier family 1 member 1 | -1.312430547 | 0.000347758 |
| ENSG00000150760 | dedicator of cytokinesis 1 | -1.313928455 | 0.001779164 |
| ENSG00000166411 | isocitrate dehydrogenase 3 (NAD(+)) alpha | -1.314640267 | 0.008390708 |
| ENSG00000149311 | ATM serine/threonine kinase | -1.315724821 | 0.00043578 |
| ENSG00000092098 | ring finger protein 31 | -1.319912358 | 0.022839246 |
| ENSG00000114166 | lysine acetyltransferase 2B | -1.319938743 | 1.85E-05 |
| ENSG00000015479 | matrin 3 | -1.320125746 | 0.02419635 |
| ENSG00000205269 | transmembrane protein 170B | -1.320851623 | 0.000389105 |
| ENSG00000146842 | transmembrane protein 209 | -1.320912723 | 1.57E-06 |
| ENSG00000255302 | EP300 interacting inhibitor of differentiation 1 | -1.321324473 | 0.000543159 |
| ENSG00000251247 | zinc finger protein 345 | -1.322122629 | 0.005714039 |
| ENSG00000180776 | zinc finger DHHC-type containing 20 | -1.323326057 | 0.003403903 |
| ENSG00000083097 | dopey family member 1 | -1.323959072 | 0.002874622 |
| ENSG00000081377 | cell division cycle 14B | -1.324797631 | 0.000778934 |
| ENSG00000136051 | KIAA1033 | -1.326438723 | 0.000151906 |
| ENSG00000001629 | ankyrin repeat and IBR domain containing 1 | -1.327196086 | 1.41E-05 |
| ENSG00000126821 | sphingosine-1-phosphate phosphatase 1 | -1.32721635 | 0.003557444 |
| ENSG00000153214 | transmembrane protein 87B | -1.327354051 | 6.07E-05 |
| ENSG00000169564 | poly(rC) binding protein 1 | -1.327788126 | 0.020414032 |
| ENSG00000067208 | ecotropic viral integration site 5 | -1.32834189 | 0.000166479 |
| ENSG00000145246 | ATPase phospholipid transporting 10D (putative) | -1.330411152 | 6.66E-05 |
| ENSG00000156030 | ELM2 and Myb/SANT domain containing 1 | -1.330917484 | 0.000450671 |
| ENSG00000156136 | deoxycytidine kinase | -1.331978407 | 0.000648126 |
| ENSG00000100764 | proteasome 26S subunit, ATPase 1 | -1.332152014 | 0.015393621 |
| ENSG00000178996 | sorting nexin 18 | -1.332161633 | 0.000147254 |
| ENSG00000108055 | structural maintenance of chromosomes 3 | -1.332872223 | 0.000266603 |
| ENSG00000106780 | multiple EGF like domains 9 | -1.334666829 | 8.15E-05 |
| ENSG00000081019 | round spermatid basic protein 1 | -1.336590243 | 4.67E-06 |
| ENSG00000175105 | zinc finger protein 654 | -1.336768251 | 0.00037607 |
| ENSG00000134758 | ring finger protein 138 | -1.336861224 | 0.00038528 |
| ENSG00000204519 | zinc finger protein 551 | -1.337722787 | 0.009720394 |
| ENSG00000143751 | SDE2 telomere maintenance homolog | -1.338459408 | 0.002924493 |
| ENSG00000181381 | DEAD-box helicase 60-like | -1.338895012 | 0.000158413 |
| ENSG00000130779 | CAP-Gly domain containing linker protein 1 | -1.339152278 | 3.85E-05 |
| ENSG00000120690 | E74 like ETS transcription factor 1 | -1.340009029 | 0.005135779 |
| ENSG00000156875 | major facilitator superfamily domain containing 14A | -1.340291874 | 0.000115259 |
| ENSG00000151718 | WW and C2 domain containing 2 | -1.340567112 | 0.003785368 |
| ENSG00000138641 | HECT and RLD domain containing E3 ubiquitin protein ligase 3 | -1.341364256 | 0.005699636 |
| ENSG00000203667 | COX20, cytochrome c oxidase assembly factor | -1.341525678 | 0.014160666 |
| ENSG00000072364 | AF4/FMR2 family member 4 | -1.34172852 | 0.000123818 |
| ENSG00000138821 | solute carrier family 39 member 8 | -1.34213558 | 2.91E-06 |
| ENSG00000120063 | G protein subunit alpha 13 | -1.343682959 | 0.000510578 |
| ENSG00000133393 | FGFR1OP N-terminal like | -1.344180752 | 0.013393061 |
| ENSG00000134602 | serine/threonine protein kinase 26 | -1.346294165 | 0.002071565 |
| ENSG00000166068 | sprouty related EVH1 domain containing 1 | -1.346691921 | 0.000250978 |
| ENSG00000179630 | laccase domain containing 1 | -1.34753824 | 0.003856752 |
| ENSG00000072401 | ubiquitin conjugating enzyme E2 D1 | -1.348895375 | 0.004648319 |
| ENSG00000163349 | homeodomain interacting protein kinase 1 | -1.350607528 | 4.85E-05 |
| ENSG00000021574 | spastin | -1.350711169 | 0.000607424 |
| ENSG00000006530 | acylglycerol kinase | -1.351484903 | 0.020225305 |
| ENSG00000150764 | DIX domain containing 1 | -1.352627442 | 0.000737186 |
| ENSG00000152558 | transmembrane protein 123 | -1.353503533 | 0.001655386 |
| ENSG00000112972 | 3-hydroxy-3-methylglutaryl-CoA synthase 1 | -1.355283689 | 0.001394111 |
| ENSG00000179981 | teashirt zinc finger homeobox 1 | -1.355827449 | 6.19E-05 |
| ENSG00000038358 | enhancer of mRNA decapping 4 | -1.356317161 | 0.010910037 |
| ENSG00000108932 | solute carrier family 16 member 6 | -1.356691596 | 0.024266998 |
| ENSG00000121879 | phosphatidylinositol-4,5-bisphosphate 3-kinase catalytic subunit alpha | -1.357369307 | 0.0008919 |
| ENSG00000130856 | zinc finger protein 236 | -1.358951008 | 0.000485785 |
| ENSG00000161298 | zinc finger protein 382 | -1.359441486 | 0.000627161 |
| ENSG00000188994 | zinc finger protein 292 | -1.360722795 | 7.03E-06 |
| ENSG00000107897 | acyl-CoA binding domain containing 5 | -1.361595608 | 1.45E-06 |
| ENSG00000123066 | mediator complex subunit 13 like | -1.364048113 | 8.73E-05 |
| ENSG00000139154 | AE binding protein 2 | -1.36626232 | 2.21E-05 |
| ENSG00000162601 | Myb like, SWIRM and MPN domains 1 | -1.366982959 | 0.001240338 |
| ENSG00000079335 | cell division cycle 14A | -1.367146681 | 0.003914168 |
| ENSG00000107164 | far upstream element binding protein 3 | -1.367663441 | 0.000931857 |
| ENSG00000102763 | von Willebrand factor A domain containing 8 | -1.369758315 | 0.000749641 |
| ENSG00000168769 | tet methylcytosine dioxygenase 2 | -1.370183147 | 0.000388483 |
| ENSG00000138071 | ARP2 actin related protein 2 homolog | -1.370650125 | 0.000255105 |
| ENSG00000138078 | prolyl endopeptidase-like | -1.371542109 | 2.19E-09 |
| ENSG00000086300 | sorting nexin 10 | -1.371551447 | 0.00266305 |
| ENSG00000072736 | nuclear factor of activated T-cells 3 | -1.372279589 | 0.000961039 |
| ENSG00000198707 | centrosomal protein 290 | -1.372778993 | 0.000242267 |
| ENSG00000134982 | APC, WNT signaling pathway regulator | -1.37382936 | 0.000412392 |
| ENSG00000150961 | SEC24 homolog D, COPII coat complex component | -1.375617345 | 0.00159386 |
| ENSG00000170456 | DENN domain containing 5B | -1.376597796 | 5.69E-10 |
| ENSG00000163848 | zinc finger protein 148 | -1.377303825 | 3.98E-05 |
| ENSG00000135250 | SRSF protein kinase 2 | -1.37788069 | 0.00049694 |
| ENSG00000144320 | lunapark, ER junction formation factor | -1.378275325 | 2.23E-05 |
| ENSG00000126581 | beclin 1 | -1.379173677 | 0.017753921 |
| ENSG00000122068 | forty-two-three domain containing 1 | -1.379384824 | 0.00035522 |
| ENSG00000164941 | integrator complex subunit 8 | -1.380227024 | 3.29E-07 |
| ENSG00000165115 | kinesin family member 27 | -1.380590308 | 0.000263448 |
| ENSG00000116791 | crystallin zeta | -1.382385286 | 3.26E-06 |
| ENSG00000178966 | RecQ mediated genome instability 1 | -1.383266412 | 0.003582366 |
| ENSG00000084093 | RE1 silencing transcription factor | -1.383408598 | 0.000535211 |
| ENSG00000152443 | zinc finger protein 776 | -1.383527538 | 0.000792006 |
| ENSG00000128791 | twisted gastrulation BMP signaling modulator 1 | -1.384273393 | 0.000134864 |
| ENSG00000175305 | cyclin E2 | -1.384656144 | 0.01388457 |
| ENSG00000144357 | ubiquitin protein ligase E3 component n-recognin 3 (putative) | -1.385199163 | 2.89E-05 |
| ENSG00000047188 | YTH domain containing 2 | -1.386232483 | 0.000145508 |
| ENSG00000076321 | kelch like family member 20 | -1.386378365 | 0.0025568 |
| ENSG00000158966 | cache domain containing 1 | -1.386600949 | 0.00025196 |
| ENSG00000173890 | G protein-coupled receptor 160 | -1.388367545 | 0.002840294 |
| ENSG00000140416 | tropomyosin 1 (alpha) | -1.388448091 | 0.026960262 |
| ENSG00000102753 | karyopherin subunit alpha 3 | -1.389335818 | 0.000906915 |
| ENSG00000176018 | LysM domain containing 3 | -1.389457704 | 0.000533179 |
| ENSG00000165029 | ATP binding cassette subfamily A member 1 | -1.39212519 | 0.006255876 |
| ENSG00000145743 | F-box and leucine rich repeat protein 17 | -1.393025191 | 0.000190577 |
| ENSG00000164754 | RAD21 cohesin complex component | -1.394268027 | 0.000531148 |
| ENSG00000169239 | carbonic anhydrase 5B | -1.394697466 | 0.000126815 |
| ENSG00000164180 | transmembrane protein 161B | -1.395596752 | 0.006331514 |
| ENSG00000155903 | RAS p21 protein activator 2 | -1.39734087 | 0.001496581 |
| ENSG00000100354 | trinucleotide repeat containing 6B | -1.401961476 | 5.72E-05 |
| ENSG00000156162 | dpy-19 like 4 (C. elegans) | -1.402396656 | 1.61E-05 |
| ENSG00000132549 | vacuolar protein sorting 13 homolog B | -1.403729329 | 0.000118078 |
| ENSG00000185722 | ankyrin repeat and FYVE domain containing 1 | -1.404170072 | 0.000285153 |
| ENSG00000171428 | N-acetyltransferase 1 | -1.405121396 | 0.003948787 |
| ENSG00000184384 | mastermind like transcriptional coactivator 2 | -1.405366295 | 0.002011223 |
| ENSG00000085224 | ATRX, chromatin remodeler | -1.40567012 | 5.99E-05 |
| ENSG00000085491 | solute carrier family 25 member 24 | -1.406140181 | 0.012222047 |
| ENSG00000183808 | RNA binding motif protein 12B | -1.408879547 | 0.004788653 |
| ENSG00000129173 | E2F transcription factor 8 | -1.409099717 | 0.007190702 |
| ENSG00000145012 | LIM domain containing preferred translocation partner in lipoma | -1.409772489 | 4.12E-05 |
| ENSG00000133731 | inositol monophosphatase 1 | -1.410284025 | 0.000214043 |
| ENSG00000162980 | ADP ribosylation factor like GTPase 5A | -1.410579006 | 0.000210094 |
| ENSG00000168970 | JMJD7-PLA2G4B readthrough | -1.412199185 | 0.012455346 |
| ENSG00000120159 | caspase activity and apoptosis inhibitor 1 | -1.412625182 | 0.000340096 |
| ENSG00000138780 | glutathione S-transferase C-terminal domain containing | -1.413373541 | 0.000853313 |
| ENSG00000151304 | serum response factor binding protein 1 | -1.413535561 | 0.000495142 |
| ENSG00000152332 | U2AF homology motif kinase 1 | -1.414134663 | 0.000214427 |
| ENSG00000116254 | chromodomain helicase DNA binding protein 5 | -1.416076599 | 0.013298032 |
| ENSG00000006576 | putative homeodomain transcription factor 2 | -1.416180957 | 4.79E-05 |
| ENSG00000155100 | OTU domain containing 6B | -1.418681016 | 2.60E-05 |
| ENSG00000151414 | NIMA related kinase 7 | -1.418975074 | 0.007446424 |
| ENSG00000162944 | raftlin family member 2 | -1.419146155 | 0.001688341 |
| ENSG00000023287 | RB1 inducible coiled-coil 1 | -1.420289761 | 6.84E-06 |
| ENSG00000197121 | post-GPI attachment to proteins 1 | -1.421818205 | 3.36E-05 |
| ENSG00000168779 | short stature homeobox 2 | -1.422836242 | 9.91E-05 |
| ENSG00000052795 | folliculin interacting protein 2 | -1.423160967 | 0.001338808 |
| ENSG00000102218 | retinitis pigmentosa 2 (X-linked recessive) | -1.423358858 | 0.002104066 |
| ENSG00000112029 | F-box protein 5 | -1.424424454 | 0.000278002 |
| ENSG00000177311 | zinc finger and BTB domain containing 38 | -1.424701073 | 0.004329287 |
| ENSG00000064313 | TATA-box binding protein associated factor 2 | -1.425477584 | 0.0031341 |
| ENSG00000131725 | WD repeat domain 44 | -1.425769479 | 0.002931294 |
| ENSG00000162607 | ubiquitin specific peptidase 1 | -1.426252959 | 1.92E-06 |
| ENSG00000135913 | ubiquitin specific peptidase 37 | -1.42831765 | 0.007605138 |
| ENSG00000006468 | ETS variant 1 | -1.429502839 | 4.08E-07 |
| ENSG00000125107 | CCR4-NOT transcription complex subunit 1 | -1.430555503 | 0.00106777 |
| ENSG00000105866 | Sp4 transcription factor | -1.431275913 | 0.003775549 |
| ENSG00000164930 | frizzled class receptor 6 | -1.433026381 | 0.00453042 |
| ENSG00000136021 | SCY1 like pseudokinase 2 | -1.434384554 | 1.01E-05 |
| ENSG00000164209 | solute carrier family 25 member 46 | -1.436272986 | 6.26E-05 |
| ENSG00000101752 | mindbomb E3 ubiquitin protein ligase 1 | -1.438373277 | 1.39E-05 |
| ENSG00000112419 | phosphatase and actin regulator 2 | -1.438954082 | 0.00067108 |
| ENSG00000168283 | BMI1 proto-oncogene, polycomb ring finger | -1.440184148 | 0.000554992 |
| ENSG00000127914 | A-kinase anchoring protein 9 | -1.441540787 | 3.47E-05 |
| ENSG00000186130 | zinc finger and BTB domain containing 6 | -1.442329125 | 0.00016731 |
| ENSG00000171853 | trafficking protein particle complex 12 | -1.445135514 | 0.010690755 |
| ENSG00000141384 | TATA-box binding protein associated factor 4b | -1.445956449 | 0.00161709 |
| ENSG00000134255 | choline/ethanolamine phosphotransferase 1 | -1.44888573 | 0.011214135 |
| ENSG00000163755 | HPS3, biogenesis of lysosomal organelles complex 2 subunit 1 | -1.452759082 | 0.002244593 |
| ENSG00000068650 | ATPase phospholipid transporting 11A | -1.452914981 | 0.001083406 |
| ENSG00000114520 | sorting nexin 4 | -1.453222675 | 0.000139315 |
| ENSG00000113595 | tripartite motif containing 23 | -1.453869748 | 6.40E-05 |
| ENSG00000129534 | MIS18 binding protein 1 | -1.456304088 | 0.001184603 |
| ENSG00000135968 | GRIP and coiled-coil domain containing 2 | -1.45821724 | 2.99E-05 |
| ENSG00000164542 | KIAA0895 | -1.460974545 | 0.000256911 |
| ENSG00000164070 | heat shock protein family A (Hsp70) member 4 like | -1.465483536 | 2.84E-09 |
| ENSG00000038274 | methionine adenosyltransferase 2B | -1.46709111 | 1.43E-05 |
| ENSG00000171940 | zinc finger protein 217 | -1.46760266 | 0.000555477 |
| ENSG00000112144 | intestinal cell kinase | -1.467684654 | 0.00042089 |
| ENSG00000172845 | Sp3 transcription factor | -1.467924326 | 0.000224608 |
| ENSG00000162643 | WD repeat domain 63 | -1.469800237 | 0.014830154 |
| ENSG00000164327 | RPTOR independent companion of MTOR complex 2 | -1.470504635 | 2.50E-05 |
| ENSG00000198964 | sphingomyelin synthase 1 | -1.474758423 | 0.00141692 |
| ENSG00000138757 | G3BP stress granule assembly factor 2 | -1.475015467 | 0.000158127 |
| ENSG00000113369 | arrestin domain containing 3 | -1.475597376 | 0.001842417 |
| ENSG00000009413 | REV3 like, DNA directed polymerase zeta catalytic subunit | -1.47695813 | 0.000410342 |
| ENSG00000104497 | sorting nexin 16 | -1.477391255 | 7.56E-05 |
| ENSG00000151023 | enkurin, TRPC channel interacting protein | -1.477508638 | 0.008397544 |
| ENSG00000168679 | solute carrier family 16 member 4 | -1.483022764 | 0.005236365 |
| ENSG00000011426 | anillin actin binding protein | -1.484092281 | 0.000124897 |
| ENSG00000080298 | regulatory factor X3 | -1.485306586 | 3.78E-08 |
| ENSG00000164187 | LMBR1 domain containing 2 | -1.486552119 | 2.33E-05 |
| ENSG00000009724 | mannan binding lectin serine peptidase 2 | -1.488009628 | 0.000741304 |
| ENSG00000162670 | BMP/retinoic acid inducible neural specific 3 | -1.488127574 | 1.75E-08 |
| ENSG00000145781 | COMM domain containing 10 | -1.490345051 | 0.000308863 |
| ENSG00000150938 | cysteine rich transmembrane BMP regulator 1 | -1.491771646 | 0.008414819 |
| ENSG00000104320 | nibrin | -1.493498406 | 2.12E-05 |
| ENSG00000137965 | interferon induced protein 44 | -1.494556599 | 0.02674154 |
| ENSG00000120137 | pantothenate kinase 3 | -1.495640088 | 1.83E-05 |
| ENSG00000112531 | QKI, KH domain containing RNA binding | -1.498818364 | 1.68E-05 |
| ENSG00000095951 | human immunodeficiency virus type I enhancer binding protein 1 | -1.499247516 | 4.32E-05 |
| ENSG00000113448 | phosphodiesterase 4D | -1.499563107 | 0.023673857 |
| ENSG00000169989 | tigger transposable element derived 4 | -1.501706739 | 0.028569138 |
| ENSG00000171150 | suppressor of cytokine signaling 5 | -1.50596838 | 0.000382996 |
| ENSG00000134313 | kinase D-interacting substrate 220kDa | -1.508242702 | 0.000365906 |
| ENSG00000136122 | bora, aurora kinase A activator | -1.508864822 | 0.00786088 |
| ENSG00000133812 | SET binding factor 2 | -1.509110047 | 0.000398027 |
| ENSG00000103479 | RB transcriptional corepressor like 2 | -1.50921677 | 0.001702744 |
| ENSG00000123104 | inositol 1,4,5-trisphosphate receptor type 2 | -1.510594356 | 4.18E-06 |
| ENSG00000163291 | progestin and adipoQ receptor family member 3 | -1.510769085 | 0.000949331 |
| ENSG00000171243 | sclerostin domain containing 1 | -1.515450308 | 0.000634468 |
| ENSG00000204920 | zinc finger protein 155 | -1.516899529 | 0.034247424 |
| ENSG00000172469 | mannosidase endo-alpha | -1.516943558 | 0.000440178 |
| ENSG00000160201 | U2 small nuclear RNA auxiliary factor 1 | -1.519481498 | 0.007419748 |
| ENSG00000076685 | 5'-nucleotidase, cytosolic II | -1.521378218 | 0.008777223 |
| ENSG00000165288 | bromodomain and WD repeat domain containing 3 | -1.521624273 | 2.10E-06 |
| ENSG00000036257 | cullin 3 | -1.522751053 | 0.000653708 |
| ENSG00000114346 | epithelial cell transforming 2 | -1.523193569 | 0.001263122 |
| ENSG00000196369 | SLIT-ROBO Rho GTPase activating protein 2B | -1.523871079 | 0.029731464 |
| ENSG00000066933 | myosin IXA | -1.524568569 | 5.79E-05 |
| ENSG00000164323 | cilia and flagella associated protein 97 | -1.525925137 | 6.25E-05 |
| ENSG00000179295 | protein tyrosine phosphatase, non-receptor type 11 | -1.527739978 | 0.004002827 |
| ENSG00000107854 | tankyrase 2 | -1.527886476 | 0.000334815 |
| ENSG00000198862 | listerin E3 ubiquitin protein ligase 1 | -1.528804605 | 0.000936139 |
| ENSG00000127870 | ring finger protein 6 | -1.530422946 | 0.000132698 |
| ENSG00000171823 | F-box and leucine rich repeat protein 14 | -1.533340366 | 0.010129458 |
| ENSG00000144063 | mal, T-cell differentiation protein like | -1.534054952 | 0.004733098 |
| ENSG00000075303 | solute carrier family 25 member 40 | -1.534503922 | 0.000127231 |
| ENSG00000067369 | tumor protein p53 binding protein 1 | -1.535592778 | 0.00674374 |
| ENSG00000138688 | KIAA1109 | -1.536563121 | 8.75E-05 |
| ENSG00000014123 | UFM1 specific ligase 1 | -1.536852562 | 1.75E-05 |
| ENSG00000176542 | upstream transcription factor family member 3 | -1.543275459 | 8.07E-05 |
| ENSG00000183337 | BCL6 corepressor | -1.543828487 | 0.000265709 |
| ENSG00000117226 | guanylate binding protein 3 | -1.545432191 | 2.31E-05 |
| ENSG00000112186 | CAP, adenylate cyclase-associated protein, 2 (yeast) | -1.545439798 | 8.94E-06 |
| ENSG00000159082 | synaptojanin 1 | -1.548246999 | 1.57E-05 |
| ENSG00000155313 | ubiquitin specific peptidase 25 | -1.549115806 | 3.60E-05 |
| ENSG00000128923 | family with sequence similarity 63 member B | -1.549541726 | 4.30E-05 |
| ENSG00000131023 | large tumor suppressor kinase 1 | -1.550331934 | 9.09E-05 |
| ENSG00000037749 | microfibrillar associated protein 3 | -1.55250293 | 0.00411075 |
| ENSG00000126746 | zinc finger protein 384 | -1.553271132 | 0.001288261 |
| ENSG00000102984 | zinc finger protein 821 | -1.553350919 | 0.001600355 |
| ENSG00000174125 | toll like receptor 1 | -1.553695238 | 0.000346957 |
| ENSG00000154240 | centrosomal protein 112 | -1.557250558 | 4.45E-07 |
| ENSG00000114331 | ArfGAP with coiled-coil, ankyrin repeat and PH domains 2 | -1.557295917 | 0.000113525 |
| ENSG00000112183 | RNA binding motif protein 24 | -1.558888485 | 2.69E-05 |
| ENSG00000166263 | syntaxin binding protein 4 | -1.559393897 | 1.20E-07 |
| ENSG00000165832 | TruB pseudouridine synthase family member 1 | -1.560319851 | 0.001015079 |
| ENSG00000173905 | golgi integral membrane protein 4 | -1.560478113 | 0.007064861 |
| ENSG00000155304 | heat shock protein family A (Hsp70) member 13 | -1.561550067 | 0.000663 |
| ENSG00000169851 | protocadherin 7 | -1.562109129 | 0.020754841 |
| ENSG00000213079 | SR-related CTD associated factor 8 | -1.56219782 | 0.000107862 |
| ENSG00000104154 | solute carrier family 30 member 4 | -1.563135692 | 8.09E-07 |
| ENSG00000122512 | PMS1 homolog 2, mismatch repair system component | -1.563956971 | 0.001868661 |
| ENSG00000070366 | SMG6, nonsense mediated mRNA decay factor | -1.566059564 | 0.018355359 |
| ENSG00000116095 | pleckstrin homology domain containing A3 | -1.566151231 | 6.34E-05 |
| ENSG00000100644 | hypoxia inducible factor 1 alpha subunit | -1.566382416 | 0.003270828 |
| ENSG00000176055 | metallo-beta-lactamase domain containing 2 | -1.569798118 | 0.000207205 |
| ENSG00000135720 | dynein cytoplasmic 1 light intermediate chain 2 | -1.569987119 | 0.007700719 |
| ENSG00000054654 | spectrin repeat containing nuclear envelope protein 2 | -1.57072131 | 6.58E-05 |
| ENSG00000119285 | HEAT repeat containing 1 | -1.574423474 | 0.001958743 |
| ENSG00000101974 | ATPase phospholipid transporting 11C | -1.575186672 | 6.54E-06 |
| ENSG00000165156 | zinc fingers and homeoboxes 1 | -1.576790067 | 2.40E-07 |
| ENSG00000104093 | Dmx like 2 | -1.577686773 | 9.22E-06 |
| ENSG00000185532 | protein kinase, cGMP-dependent, type I | -1.579007189 | 0.035014394 |
| ENSG00000159023 | erythrocyte membrane protein band 4.1 | -1.580613708 | 0.000185889 |
| ENSG00000128731 | HECT and RLD domain containing E3 ubiquitin protein ligase 2 | -1.581233134 | 0.009311698 |
| ENSG00000118496 | F-box protein 30 | -1.586377942 | 2.31E-05 |
| ENSG00000205730 | inositol 1,4,5-trisphosphate receptor interacting protein like 2 | -1.586881473 | 0.007493756 |
| ENSG00000115760 | baculoviral IAP repeat containing 6 | -1.588475908 | 0.000109108 |
| ENSG00000155008 | apolipoprotein O like | -1.588543418 | 9.59E-05 |
| ENSG00000198301 | SDA1 domain containing 1 | -1.589716915 | 0.003063891 |
| ENSG00000179941 | Bardet-Biedl syndrome 10 | -1.593875545 | 3.30E-05 |
| ENSG00000171988 | jumonji domain containing 1C | -1.597862013 | 3.87E-05 |
| ENSG00000067900 | Rho associated coiled-coil containing protein kinase 1 | -1.599539094 | 1.15E-05 |
| ENSG00000102531 | fibronectin type III domain containing 3A | -1.5996358 | 5.59E-05 |
| ENSG00000187775 | dynein axonemal heavy chain 17 | -1.6014973 | 0.023267938 |
| ENSG00000184979 | ubiquitin specific peptidase 18 | -1.601774258 | 0.01308543 |
| ENSG00000115020 | phosphoinositide kinase, FYVE-type zinc finger containing | -1.603812087 | 6.36E-05 |
| ENSG00000164164 | OTU deubiquitinase 4 | -1.605884211 | 0.000269842 |
| ENSG00000123416 | tubulin alpha 1b | -1.605908802 | 0.033079499 |
| ENSG00000151693 | ArfGAP with SH3 domain, ankyrin repeat and PH domain 2 | -1.60628523 | 0.014044623 |
| ENSG00000055609 | lysine methyltransferase 2C | -1.610842839 | 1.84E-05 |
| ENSG00000198087 | CD2 associated protein | -1.61203786 | 5.96E-06 |
| ENSG00000139687 | RB transcriptional corepressor 1 | -1.615780827 | 0.000154234 |
| ENSG00000132274 | tripartite motif containing 22 | -1.617244043 | 0.00162331 |
| ENSG00000184226 | protocadherin 9 | -1.618716531 | 0.005283997 |
| ENSG00000123091 | ring finger protein 11 | -1.626681407 | 0.000107717 |
| ENSG00000274276 | cystathionine-beta-synthase like | -1.627791552 | 0.016016518 |
| ENSG00000171126 | potassium voltage-gated channel modifier subfamily G member 3 | -1.629638166 | 0.013056604 |
| ENSG00000113638 | tetratricopeptide repeat domain 33 | -1.630937752 | 0.00051965 |
| ENSG00000019995 | zinc finger RANBP2-type containing 1 | -1.631461087 | 0.008440451 |
| ENSG00000139613 | SWI/SNF related, matrix associated, actin dependent regulator of chromatin subfamily c member 2 | -1.631780499 | 0.002736074 |
| ENSG00000100934 | Sec23 homolog A, coat complex II component | -1.632689833 | 0.000909674 |
| ENSG00000023516 | A-kinase anchoring protein 11 | -1.635695665 | 1.58E-05 |
| ENSG00000114805 | phospholipase C eta 1 | -1.637189551 | 0.021196789 |
| ENSG00000170677 | suppressor of cytokine signaling 6 | -1.639156399 | 2.03E-08 |
| ENSG00000127995 | CAS1 domain containing 1 | -1.639505182 | 0.001769636 |
| ENSG00000228253 | mitochondrially encoded ATP synthase 8 | -1.640902677 | 0.028648709 |
| ENSG00000166046 | t-complex 11 like 2 | -1.641298918 | 0.011371366 |
| ENSG00000174628 | IQ motif containing K | -1.642283434 | 0.001690815 |
| ENSG00000096968 | Janus kinase 2 | -1.64296533 | 3.06E-05 |
| ENSG00000134970 | transmembrane p24 trafficking protein 7 | -1.645734674 | 0.000337767 |
| ENSG00000170632 | armadillo repeat containing 10 | -1.648739543 | 0.000625397 |
| ENSG00000182890 | glutamate dehydrogenase 2 | -1.652913677 | 0.022918184 |
| ENSG00000197579 | TOP1 binding arginine/serine rich protein | -1.658370392 | 0.000246171 |
| ENSG00000198961 | praja ring finger ubiquitin ligase 2 | -1.660541186 | 2.67E-05 |
| ENSG00000165244 | zinc finger protein 367 | -1.661758459 | 0.000108979 |
| ENSG00000172915 | neurobeachin | -1.662875618 | 2.31E-05 |
| ENSG00000005249 | protein kinase cAMP-dependent type II regulatory subunit beta | -1.666425874 | 0.001274402 |
| ENSG00000141232 | transducer of ERBB2, 1 | -1.668093734 | 0.000394622 |
| ENSG00000059758 | cyclin dependent kinase 17 | -1.670891242 | 3.49E-05 |
| ENSG00000075223 | semaphorin 3C | -1.672645039 | 6.79E-05 |
| ENSG00000177888 | zinc finger and BTB domain containing 41 | -1.676875869 | 0.000632444 |
| ENSG00000138468 | SUMO1/sentrin specific peptidase 7 | -1.678224695 | 2.57E-07 |
| ENSG00000101596 | structural maintenance of chromosomes flexible hinge domain containing 1 | -1.680194906 | 2.80E-06 |
| ENSG00000164649 | cell division cycle associated 7 like | -1.682893599 | 0.003112141 |
| ENSG00000226763 | serine/arginine repetitive matrix 5 | -1.685362143 | 0.032186848 |
| ENSG00000120992 | lysophospholipase I | -1.685487933 | 0.000227576 |
| ENSG00000168386 | filamin A interacting protein 1 like | -1.686788882 | 0.022014554 |
| ENSG00000148516 | zinc finger E-box binding homeobox 1 | -1.689650347 | 3.39E-06 |
| ENSG00000178385 | pleckstrin homology domain containing M3 | -1.697168821 | 0.000293695 |
| ENSG00000205413 | sterile alpha motif domain containing 9 | -1.699367429 | 1.30E-08 |
| ENSG00000151151 | inositol polyphosphate multikinase | -1.700251693 | 5.12E-06 |
| ENSG00000145725 | diphosphoinositol pentakisphosphate kinase 2 | -1.701548298 | 3.43E-05 |
| ENSG00000087301 | thioredoxin domain containing 16 | -1.701831251 | 0.000288018 |
| ENSG00000157107 | FCH domain only 2 | -1.702034314 | 4.51E-05 |
| ENSG00000120519 | solute carrier family 10 member 7 | -1.702636244 | 0.003354887 |
| ENSG00000140548 | zinc finger protein 710 | -1.704323151 | 0.002443645 |
| ENSG00000164236 | ankyrin repeat domain 33B | -1.7048245 | 0.000145501 |
| ENSG00000109654 | tripartite motif containing 2 | -1.704966658 | 0.000451105 |
| ENSG00000132294 | EFR3 homolog A | -1.705561054 | 3.21E-07 |
| ENSG00000138593 | SECIS binding protein 2 like | -1.70686366 | 1.27E-05 |
| ENSG00000169246 | nuclear pore complex interacting protein family member B3 | -1.708854172 | 0.022357054 |
| ENSG00000117228 | guanylate binding protein 1 | -1.711319058 | 1.23E-07 |
| ENSG00000118007 | stromal antigen 1 | -1.714064701 | 0.000940719 |
| ENSG00000186522 | septin 10 | -1.714144282 | 9.53E-05 |
| ENSG00000156531 | PHD finger protein 6 | -1.714772308 | 1.68E-05 |
| ENSG00000184898 | RNA binding motif protein 43 | -1.716848126 | 3.23E-05 |
| ENSG00000114904 | NIMA related kinase 4 | -1.719662372 | 0.002469033 |
| ENSG00000004700 | RecQ like helicase | -1.720157237 | 0.008465117 |
| ENSG00000188641 | dihydropyrimidine dehydrogenase | -1.7230577 | 5.73E-10 |
| ENSG00000168702 | LDL receptor related protein 1B | -1.727608965 | 0.000151473 |
| ENSG00000205707 | electron transfer flavoprotein regulatory factor 1 | -1.733448696 | 9.63E-05 |
| ENSG00000269028 | MT-RNR2-like 12 | -1.736050136 | 0.013304879 |
| ENSG00000142794 | neuroblastoma breakpoint family member 3 | -1.73711542 | 0.003872991 |
| ENSG00000118412 | caspase 8 associated protein 2 | -1.739239676 | 0.000271731 |
| ENSG00000065809 | family with sequence similarity 107 member B | -1.739657034 | 2.79E-11 |
| ENSG00000108021 | family with sequence similarity 208 member B | -1.746228236 | 7.67E-05 |
| ENSG00000174776 | WD repeat domain 49 | -1.74745043 | 0.009440677 |
| ENSG00000117054 | acyl-CoA dehydrogenase, C-4 to C-12 straight chain | -1.751665951 | 4.40E-06 |
| ENSG00000100129 | eukaryotic translation initiation factor 3 subunit L | -1.761006584 | 0.019201146 |
| ENSG00000100852 | Rho GTPase activating protein 5 | -1.761872239 | 0.000244546 |
| ENSG00000164830 | oxidation resistance 1 | -1.76241337 | 0.004662393 |
| ENSG00000105855 | integrin subunit beta 8 | -1.763804004 | 4.68E-05 |
| ENSG00000165985 | complement C1q like 3 | -1.764174361 | 0.009051944 |
| ENSG00000121743 | gap junction protein alpha 3 | -1.76624327 | 0.000259549 |
| ENSG00000116266 | syntaxin binding protein 3 | -1.772940972 | 1.66E-05 |
| ENSG00000159256 | MORC family CW-type zinc finger 3 | -1.774072871 | 0.000529875 |
| ENSG00000166004 | centrosomal protein 295 | -1.775608308 | 0.01574744 |
| ENSG00000198718 | family with sequence similarity 179 member B | -1.776771058 | 0.000612476 |
| ENSG00000179454 | kelch like family member 28 | -1.777125515 | 0.009501397 |
| ENSG00000185567 | AHNAK nucleoprotein 2 | -1.777688911 | 8.93E-05 |
| ENSG00000102189 | early endosome antigen 1 | -1.779453929 | 0.000172774 |
| ENSG00000065243 | protein kinase N2 | -1.788963591 | 2.56E-05 |
| ENSG00000139209 | solute carrier family 38 member 4 | -1.795913609 | 9.77E-06 |
| ENSG00000132530 | XIAP associated factor 1 | -1.797849053 | 0.000513804 |
| ENSG00000136144 | RCC1 and BTB domain containing protein 1 | -1.802964915 | 6.42E-05 |
| ENSG00000166439 | ring finger protein 169 | -1.803585956 | 0.001238256 |
| ENSG00000106460 | transmembrane protein 106B | -1.810212981 | 1.57E-05 |
| ENSG00000164023 | sphingomyelin synthase 2 | -1.815107451 | 7.88E-05 |
| ENSG00000145687 | single stranded DNA binding protein 2 | -1.820112604 | 6.37E-15 |
| ENSG00000140382 | high mobility group 20A | -1.825097618 | 0.006288391 |
| ENSG00000111816 | fyn related Src family tyrosine kinase | -1.826030938 | 0.004910113 |
| ENSG00000129003 | vacuolar protein sorting 13 homolog C | -1.829436107 | 5.77E-06 |
| ENSG00000011405 | phosphatidylinositol-4-phosphate 3-kinase catalytic subunit type 2 alpha | -1.830534978 | 1.42E-06 |
| ENSG00000198677 | tetratricopeptide repeat domain 37 | -1.835906793 | 4.82E-06 |
| ENSG00000182568 | SATB homeobox 1 | -1.840968585 | 3.02E-07 |
| ENSG00000197479 | protocadherin beta 11 | -1.843893366 | 0.000285675 |
| ENSG00000066557 | leucine rich repeat containing 40 | -1.852435937 | 0.000132331 |
| ENSG00000033122 | leucine rich repeat containing 7 | -1.854392911 | 0.004266232 |
| ENSG00000219545 | UBAP1-MVB12-associated (UMA) domain containing 1 | -1.860602773 | 5.37E-05 |
| ENSG00000057657 | PR/SET domain 1 | -1.864849784 | 0.000440813 |
| ENSG00000140396 | nuclear receptor coactivator 2 | -1.866018412 | 0.000136598 |
| ENSG00000144857 | BOC cell adhesion associated, oncogene regulated | -1.866438522 | 0.001499093 |
| ENSG00000276966 | histone cluster 1 H4 family member e | -1.877589844 | 0.013269516 |
| ENSG00000138660 | adaptor related protein complex 1 associated regulatory protein | -1.882359203 | 8.52E-05 |
| ENSG00000008086 | cyclin dependent kinase like 5 | -1.896927391 | 7.21E-06 |
| ENSG00000112210 | RAB23, member RAS oncogene family | -1.897918892 | 0.010654762 |
| ENSG00000137145 | DENN domain containing 4C | -1.909498671 | 3.42E-06 |
| ENSG00000132854 | KN motif and ankyrin repeat domains 4 | -1.91220558 | 3.46E-05 |
| ENSG00000151694 | ADAM metallopeptidase domain 17 | -1.912532047 | 0.02716709 |
| ENSG00000107864 | cytoplasmic polyadenylation element binding protein 3 | -1.924578349 | 0.008645733 |
| ENSG00000174808 | betacellulin | -1.935208888 | 7.33E-05 |
| ENSG00000113810 | structural maintenance of chromosomes 4 | -1.937253162 | 0.000681321 |
| ENSG00000162669 | HFM1, ATP dependent DNA helicase homolog | -1.943740051 | 0.015141437 |
| ENSG00000156675 | RAB11 family interacting protein 1 | -1.944366111 | 0.009258351 |
| ENSG00000166689 | pleckstrin homology domain containing A7 | -1.945373829 | 2.30E-07 |
| ENSG00000111325 | 2-oxoglutarate and iron dependent oxygenase domain containing 2 | -1.946053769 | 0.035470276 |
| ENSG00000151835 | sacsin molecular chaperone | -1.951831213 | 6.17E-06 |
| ENSG00000174953 | DEAH-box helicase 36 | -1.952287967 | 0.032055007 |
| ENSG00000102908 | nuclear factor of activated T-cells 5 | -1.954301366 | 0.000390646 |
| ENSG00000263001 | general transcription factor IIi | -1.956337222 | 1.26E-05 |
| ENSG00000186767 | spindlin family member 4 | -1.962820063 | 0.000205279 |
| ENSG00000278272 | histone cluster 1 H3 family member c | -1.965642867 | 0.018638019 |
| ENSG00000166398 | KIAA0355 | -1.966617618 | 0.002902236 |
| ENSG00000136231 | insulin like growth factor 2 mRNA binding protein 3 | -1.969645705 | 0.000380303 |
| ENSG00000116678 | leptin receptor | -1.983591704 | 0.014532055 |
| ENSG00000028116 | vaccinia related kinase 2 | -1.98865152 | 0.000393193 |
| ENSG00000167193 | CRK proto-oncogene, adaptor protein | -1.996144087 | 0.024344524 |
| ENSG00000091039 | oxysterol binding protein like 8 | -1.997300623 | 1.57E-07 |
| ENSG00000099617 | ephrin A2 | -2.001874761 | 0.030362062 |
| ENSG00000278705 | histone cluster 1 H4 family member b | -2.012581548 | 0.030589969 |
| ENSG00000169282 | potassium voltage-gated channel subfamily A member regulatory beta subunit 1 | -2.020227492 | 0.016692579 |
| ENSG00000187372 | protocadherin beta 13 | -2.042285207 | 0.007439302 |
| ENSG00000198146 | zinc finger protein 770 | -2.042834338 | 0.001444438 |
| ENSG00000146414 | SNF2 histone linker PHD RING helicase | -2.050725139 | 0.003039999 |
| ENSG00000278463 | histone cluster 1 H2A family member b | -2.055581165 | 0.027418948 |
| ENSG00000138336 | tet methylcytosine dioxygenase 1 | -2.062546404 | 1.82E-05 |
| ENSG00000179071 | coiled-coil domain containing 89 | -2.081680953 | 0.007254192 |
| ENSG00000198743 | solute carrier family 5 member 3 | -2.081699671 | 2.86E-05 |
| ENSG00000185630 | PBX homeobox 1 | -2.091259863 | 0.014249506 |
| ENSG00000263513 | family with sequence similarity 72 member C | -2.094840529 | 0.03367491 |
| ENSG00000174501 | ankyrin repeat domain 36C | -2.096041861 | 0.010108049 |
| ENSG00000131019 | UL16 binding protein 3 | -2.105812367 | 0.018570677 |
| ENSG00000011566 | mitogen-activated protein kinase kinase kinase kinase 3 | -2.109794123 | 5.45E-05 |
| ENSG00000078177 | NEDD4 binding protein 2 | -2.127948512 | 0.000800767 |
| ENSG00000168916 | zinc finger protein 608 | -2.145504897 | 7.03E-06 |
| ENSG00000074527 | netrin 4 | -2.15189056 | 0.012540495 |
| ENSG00000162924 | REL proto-oncogene, NF-kB subunit | -2.162182912 | 0.000383788 |
| ENSG00000161847 | ribonucleoprotein, PTB binding 1 | -2.167007012 | 0.022425456 |
| ENSG00000140284 | solute carrier family 27 member 2 | -2.170360351 | 0.01779434 |
| ENSG00000153707 | protein tyrosine phosphatase, receptor type D | -2.173231551 | 9.29E-05 |
| ENSG00000135976 | ankyrin repeat domain 36 | -2.183928217 | 0.001493288 |
| ENSG00000283297 | Uncharacterized protein ENSP00000372125 | -2.19028604 | 0.03336054 |
| ENSG00000020181 | adhesion G protein-coupled receptor A2 | -2.191756903 | 0.003921457 |
| ENSG00000170160 | coiled-coil domain containing 144A | -2.204458975 | 0.012726456 |
| ENSG00000174945 | archaelysin family metallopeptidase 1 | -2.206161638 | 0.034372611 |
| ENSG00000119138 | Kruppel like factor 9 | -2.207501285 | 2.25E-07 |
| ENSG00000152936 | lamin tail domain containing 1 | -2.21163328 | 9.83E-05 |
| ENSG00000137628 | DExD/H-box helicase 60 | -2.212820039 | 4.28E-08 |
| ENSG00000196912 | ankyrin repeat domain 36B | -2.213569828 | 0.012401865 |
| ENSG00000188211 | natural killer cell cytotoxicity receptor 3 ligand 1 | -2.230838513 | 0.004637748 |
| ENSG00000196747 | histone cluster 1 H2A family member i | -2.233601337 | 0.008166851 |
| ENSG00000178502 | kelch like family member 11 | -2.243449919 | 0.033881337 |
| ENSG00000007237 | growth arrest specific 7 | -2.251024398 | 0.023408761 |
| ENSG00000131849 | zinc finger protein 132 | -2.256914152 | 0.000660537 |
| ENSG00000273703 | histone cluster 1 H2B family member m | -2.257718629 | 0.024876143 |
| ENSG00000147257 | glypican 3 | -2.261062368 | 0.024874638 |
| ENSG00000274267 | histone cluster 1 H3 family member b | -2.287112209 | 0.016278821 |
| ENSG00000123500 | collagen type X alpha 1 chain | -2.29866368 | 0.003104881 |
| ENSG00000197153 | histone cluster 1 H3 family member j | -2.313364108 | 0.021235675 |
| ENSG00000132405 | TBC1 domain family member 14 | -2.315028277 | 0.00144796 |
| ENSG00000115464 | ubiquitin specific peptidase 34 | -2.318971388 | 0.00892746 |
| ENSG00000151239 | twinfilin actin binding protein 1 | -2.322703975 | 2.18E-05 |
| ENSG00000180336 | meiosis specific with coiled-coil domain | -2.332686232 | 0.004104881 |
| ENSG00000163513 | transforming growth factor beta receptor 2 | -2.369368206 | 0.015099905 |
| ENSG00000134765 | desmocollin 1 | -2.378264132 | 0.026008403 |
| ENSG00000168298 | histone cluster 1 H1 family member e | -2.378348039 | 0.003923786 |
| ENSG00000089505 | CKLF like MARVEL transmembrane domain containing 1 | -2.396032967 | 0.016980799 |
| ENSG00000185261 | KIAA0825 | -2.411486395 | 0.015137591 |
| ENSG00000274997 | histone cluster 1 H2A family member h | -2.42740248 | 0.007153736 |
| ENSG00000139155 | solute carrier organic anion transporter family member 1C1 | -2.444853079 | 0.027433104 |
| ENSG00000204967 | protocadherin alpha 4 | -2.457474293 | 0.000586093 |
| ENSG00000135333 | EPH receptor A7 | -2.472003179 | 2.57E-07 |
| ENSG00000180530 | nuclear receptor interacting protein 1 | -2.48983597 | 0.008433334 |
| ENSG00000274070 | GATS protein like 2 | -2.543390001 | 0.011529653 |
| ENSG00000106560 | GTPase, IMAP family member 2 | -2.556946487 | 2.85E-07 |
| ENSG00000260007 |  | -2.573868786 | 0.004353114 |
| ENSG00000197061 | histone cluster 1 H4 family member c | -2.597198139 | 0.011828402 |
| ENSG00000166415 | WD repeat domain 72 | -2.611893723 | 0.012056099 |
| ENSG00000124575 | histone cluster 1 H1 family member d | -2.660202216 | 0.019316543 |
| ENSG00000165181 | chromosome 9 open reading frame 84 | -2.667358426 | 0.005981925 |
| ENSG00000184357 | histone cluster 1 H1 family member b | -2.710750291 | 0.019511801 |
| ENSG00000090061 | cyclin K | -2.729834518 | 0.001252833 |
| ENSG00000254221 | protocadherin gamma subfamily B, 1 | -2.786847745 | 0.005001898 |
| ENSG00000233087 | RAB6C-like | -2.881208751 | 0.003996778 |
| ENSG00000173114 | leucine rich repeat neuronal 3 | -2.899557841 | 0.012021497 |
| ENSG00000106819 | asporin | -2.955871767 | 0.005770581 |
| ENSG00000205086 | chromosome 2 open reading frame 91 | -3.116536344 | 0.00123698 |
| ENSG00000126861 | oligodendrocyte myelin glycoprotein | -3.124325028 | 0.012031864 |
| ENSG00000112309 | beta-1,3-glucuronyltransferase 2 | -3.191748075 | 0.006050953 |
| ENSG00000171401 | keratin 13 | -3.264053017 | 0.001452013 |
| ENSG00000275895 | U2 small nuclear RNA auxiliary factor 1 like 5 | -3.297958516 | 0.000984828 |
| ENSG00000143632 | actin, alpha 1, skeletal muscle | -3.467746807 | 0.03231394 |
| ENSG00000185013 | 5'-nucleotidase, cytosolic IB | -3.469724606 | 0.012476806 |
| ENSG00000175084 | desmin | -3.487374056 | 0.009325227 |
| ENSG00000102931 | ADP ribosylation factor like GTPase 2 binding protein | -3.497797691 | 0.003987852 |
| ENSG00000146006 | leucine rich repeat transmembrane neuronal 2 | -3.602620255 | 0.004208546 |
| ENSG00000018625 | ATPase Na+/K+ transporting subunit alpha 2 | -3.618461545 | 0.000930139 |
| ENSG00000143341 | hemicentin 1 | -3.797551281 | 0.013445461 |
| ENSG00000159251 | actin, alpha, cardiac muscle 1 | -4.259960397 | 0.001090774 |
| ENSG00000187537 | POTE ankyrin domain family member G | -4.496220098 | 0.002367904 |
| ENSG00000145423 | secreted frizzled related protein 2 | -4.504219695 | 0.022899694 |
| ENSG00000155657 | titin | -4.668892526 | 0.02974202 |
| ENSG00000169926 | Kruppel like factor 13 | -5.26581859 | 0.000261576 |
| ENSG00000133020 | myosin heavy chain 8 | -21.32889923 | 5.28E-07 |
